# Deep insight into cytokine storm: from pathogenesis to treatment

**DOI:** 10.1038/s41392-025-02178-y

**Published:** 2025-04-16

**Authors:** Jiali Nie, Ling Zhou, Weiwei Tian, Xiansheng Liu, Liping Yang, Xingcheng Yang, Yicheng Zhang, Shuang Wei, Dao Wen Wang, Jia Wei

**Affiliations:** 1 Division of Cardiology, Department of Internal Medicine, Tongji Hospital, Tongji Medical College, Huazhong University of Science and Technology, Wuhan, China; 2 Hubei Key Laboratory of Genetics and Molecular Mechanisms of Cardiological Disorders, Wuhan, China; 3 Department of Respiratory and Critical Care Medicine, National Health Commission (NHC) Key Laboratory of Respiratory Disease, Tongji Hospital, Tongji Medical College, Huazhong University of Science and Technology, Wuhan, China; 4 Hubei Branch of National Clinical Research Center for Infectious Diseases, Wuhan Pulmonary Hospital (Wuhan Tuberculosis Prevention and Control Institute), Wuhan, China; 5 Department of Hematology, Shanxi Bethune Hospital, Shanxi Academy of Medical Sciences, Tongji Shanxi Hospital, Third Hospital of Shanxi Medical University, Taiyuan, China; 6 Sino-German Joint Oncological Research Laboratory, Shanxi Bethune Hospital, Shanxi Academy of Medical Sciences, Taiyuan, China; 7 Department of Hematology, Tongji Hospital, Tongji Medical College, Huazhong University of Science and Technology, Wuhan, China; 8 Immunotherapy Research Center for Hematologic Diseases of Hubei Province, Tongji Hospital, Tongji Medical College, Huazhong University of Science and Technology, Wuhan, China

**Keywords:** Inflammation, Immunological disorders

## Abstract

Cytokine storm (CS) is a severe systemic inflammatory syndrome characterized by the excessive activation of immune cells and a significant increase in circulating levels of cytokines. This pathological process is implicated in the development of life-threatening conditions such as fulminant myocarditis (FM), acute respiratory distress syndrome (ARDS), primary or secondary hemophagocytic lymphohistiocytosis (HLH), cytokine release syndrome (CRS) associated with chimeric antigen receptor-modified T (CAR-T) therapy, and grade III to IV acute graft-versus-host disease following allogeneic hematopoietic stem cell transplantation. The significant involvement of the JAK-STAT pathway, Toll-like receptors, neutrophil extracellular traps, NLRP3 inflammasome, and other signaling pathways has been recognized in the pathogenesis of CS. Therapies targeting these pathways have been developed or are currently being investigated. While novel drugs have demonstrated promising therapeutic efficacy in mitigating CS, the overall mortality rate of CS resulting from underlying diseases remains high. In the clinical setting, the management of CS typically necessitates a multidisciplinary team strategy encompassing the removal of abnormal inflammatory or immune system activation, the preservation of vital organ function, the treatment of the underlying disease, and the provision of life supportive therapy. This review provides a comprehensive overview of the key signaling pathways and associated cytokines implicated in CS, elucidates the impact of dysregulated immune cell activation, and delineates the resultant organ injury associated with CS. In addition, we offer insights and current literature on the management of CS in cases of FM, ARDS, systemic inflammatory response syndrome, treatment-induced CRS, HLH, and other related conditions.

## Introduction

**Fig. 1:**
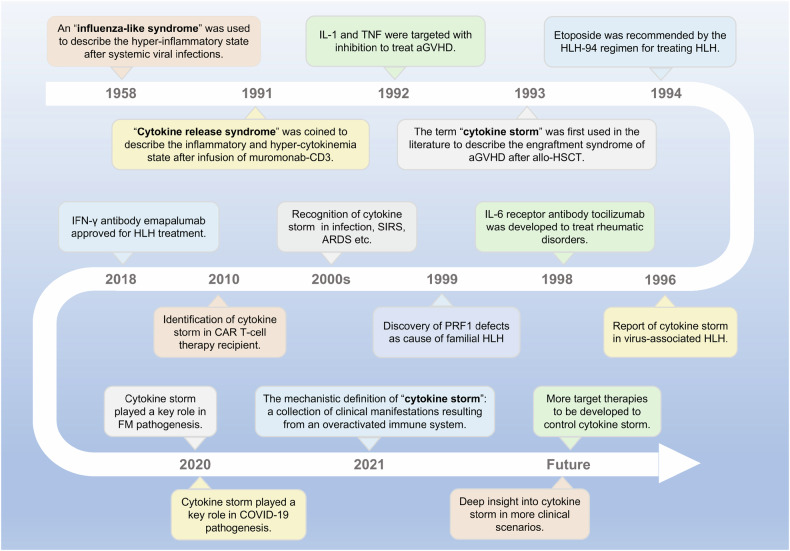
Timeline of insight into cytokine storm. The figure was created with the assistance of Powerpoint

The cytokine storm (CS) is a life-threatening systemic inflammatory syndrome characterized by hyperactivation of immune cells and elevated levels of circulating cytokines.^1–5^ The clinical presentation includes acute systemic inflammatory symptoms, organ dysfunction, and mortality. Although the term “CS” was first coined in 1993, recognition of this hyperinflammatory state can be traced back to earlier literature (Fig. 1), with references to an “influenza-like syndrome” in 1958 to describe the exaggerated immune response following systemic viral infections.^1,6^ In 1991, the term “cytokine release syndrome” (CRS) was coined to characterize the inflammatory and hypercytokinemia state following muromonab-CD3 infusion, highlighting the significant role of cytokines in the pathogenesis of the condition.^7^ Subsequently, the targeting of interleukin-1 (IL-1) and tumor necrosis factors (TNF) with inhibition was explored as a treatment approach for acute graft-versus-host disease (aGVHD).^8,9^ By 1993, the term “CS” was first utilized to describe the engraftment syndrome associated with aGVHD following allogeneic hematopoietic stem cell transplantation (allo-HSCT).^10^ Subsequent to this, a greater understanding of CS has been achieved through the examination of various clinical contexts,^11^ including immunotherapies,^12,13^ pathogens, cancers,^14^ autoimmune diseases, and monogenic diseases.^15,16^ However, the precise mechanism of initiation remains incompletely elucidated. CS entails intricate interactions among various immune cells, cytokines (Table 1), and chemokines (Table 2). Due to the deleterious effects of CS, extensive research efforts have been undertaken to elucidate the pathophysiology of CS in various diseases and to investigate potential therapeutic strategies for its management. The presence of CS has been documented in various infectious contexts, including cytomegalovirus, Epstein-Barr virus (EBV), influenza virus, variola virus, and severe acute respiratory syndrome coronavirus (SARS-CoV), as well as in non-infectious conditions such as aGVHD, hemophagocytic lymphohistiocytosis (HLH), acute respiratory distress syndrome (ARDS), and rheumatic disorders.^11^ Efforts have been made to create predictive models for the early detection of CS. The HScore^17^ and MS score^18^ are commonly utilized in the evaluation of CS associated with HLH, while the Common Terminology Criteria for Adverse Events grading system is more commonly employed for the assessment of CS in various contexts. Additionally, numerous cytokine antibodies and inhibitors have been developed to target and inhibit cytokine cascades in diseases, such as antibodies targeting IL-1, IL-6, IL-18, TNF, interferon (IFN)-γ, as well as Janus kinase (JAK) inhibitors, caspase inhibitors and calcineurin inhibitors.^11,19^

**Table 1:** Common cytokines and their features

Cytokines	Main cell type	Major Function
IL-1	Macrophages, epithelial cells, pyroptic cells	Pro-inflammatory; pyrogenic; macrophage activation; Th17 cells differentiation.^563^
IL-2	T cells	Autoimmunity regulation; T cell proliferation and differentiation; Teff generation; Treg maintenance.^564^
IL-4	Mast cells, Th2 cells, eosinophils, and basophils	Anti-inflammatory; IgE production; Th2 differentiation; M2 macrophage polarization^155^
IL-6	Macrophages, T cells, fibroblasts, endothelial cells	Pro-inflammatory; acute phase response; pyrogenic; angiogenic; T cell differentiation; enhanced antibody production, increased vascular permeability.^565^
IL-9	Th9 cells, type 2 innate lymphoid cells, Tc9 cells, Vδ2 T cells, mast cells	Pleiotropic; anti-tumor; T cells and B cells regulation; mast cells activation.^566^
IL-10	Th2 cells, Treg cells, CD8^+^ T cells, B cells	Anti-inflammatory; suppression of immune response; inhibition of macrophage activation; inhibition of Th1 cells; Treg response.^567^
IL-12	DCs, macrophages	Th1 cell differentiation; activation of T and NK cells; inhibition of immunosuppressive cells; induction of IFN-γ production; action in synergy with IL-18.^568^
IL-13	Th2 cells	Anti-inflammatory; B cell proliferation; activation of eosinophils, basophils, and mast cells.^569^
IL-17	Th17 cells, Tc17 cells, NK cells, γδ T cells, type 3 innate lymphoid cells	Pro-inflammatory; bacterial elimination; induction of cytokines and chemokines; immune cell recruitment.^570^
IL-18	Macrophages, DCs	Pro-inflammatory; activation of Th1 cells; action in synergy with IL-12.^571^
IL-21	Tfh cells	Pro-inflammatory; B cell activation; CD8^+^ T differentiation and activation.^572^
IL-22	Th1, Th17, Th22, CD8^+^ T cells, γδ T cells, NK cells, neutrophils	Regulation of host defense and epithelial homeostasis; antimicrobial.^573^
IL-31	Th2 cells, macrophages, DCs, eosinophils, mast cells, fibroblasts and keratinocytes	Pro-inflammatory; cell mediated immunity; itch mediator.^574^
IL-33	Endothelial cells, epithelial cells, macrophages, DCs, mast cells	Pro-inflammatory; activation of Th1, Th2, NK cells, CD8^+^ T cells; allergic inflammation.^575^
IL-37	Macrophages, DCs, epithelial cells, Treg cells	Anti-inflammatory; suppression of innate inflammatory and immune responses.^576^
Type I IFN	Virtually all body cells	Antimicrobial activity; modulation of innate immune responses; activate the adaptive immune system.^577^
Type II IFN	NK cells, Th1 cells, cytotoxic T cells	Proinflammatory; antiviral immunity, regulation of innate and adaptive immune responses.^578^
TGF-β	Almost every tissue and cell type	Immunosuppressive; oncogenic; regulation of cell proliferation, embryonic development, wound healing, and immune response.^579^
TNF	T cells, NK cells, macrophages, mast cells	Pyrogenic; increasing vascular permeability.^580^

*DCs* dendritic cells, *IL* interleukin, *Th* *cells* helper T cells, *Teff* effector T cells, *Treg* regulatory T cells, *NK* natural killer T cells, *Tfh* follicular helper T cells, *TGF* tumor growth factor, *TNF* tumor necrosis factor

**Table 2:** Common chemokines and their features

Chemokines	Main cell type	Major Function
MCP-1 (CCL2)	Macrophages, epithelial cells, endothelial cells, smooth muscle cells, fibroblasts	Pro-inflammatory; recruitment of macrophages, Th1 cells, and NK cells; induction of cytokines.^581^
MIP-1α (CCL3)	Macrophages, neutrophils, lymphocytes, NK cells, epithelial cells, fibroblasts	Immune surveillance and tolerance; Recruitment of macrophages, NK cells, Th1 cells, and DCs.^582^
MIP-1β (CCL4)	Macrophages, CD8^+^ T cells, NK cells, B cells, neutrophils	Oncogenic; recruitment of macrophages, lymphocytes, NK cells, and neutrophils.^583^
IL-8 (CXCL8)	Macrophages, epithelial cells, endothelial cells	Neutrophil chemotaxis.^584^
MIG (CXCL9)	Monocytes, endothelial cells, keratinocytes	IFN-inducible chemokine; recruitment of Th1 cells, NK cells, and plasmacytoid DCs.^585^
IP-10 (CXCL10)	Macrophages, DCs, T cells, NK cells	Pathogenesis of autoimmunity; recruitment and activation of Th1 cells, macrophages, and NK cells.^585^
BLC (CXCL13)	B cells, DCs	Recruitment of B cells, Th1 cells, macrophages, and DCs.^586^

*CCL* chemokine ligand, *CXCL* CXC-motif chemokine ligand, *MCP-1* monocyte chemoattractant protein 1, *MIP* macrophage inflammatory protein, *MIG* monokine induced by interferon-gamma, *IP-10* interferon-induced protein 10, *BLC* B lymphocyte chemoattractant

Unregulated inflammatory processes and extensive cytokine cascades are intricately linked to a range of critical clinical conditions, including fulminant myocarditis (FM),^20,21^ ARDS,^22^ systemic inflammatory response syndrome (SIRS),^23,24^ HLH,^25^ aGVHD,^26^ and CRS associated with chimeric antigen receptor-modified T (CAR-T) therapy.^27,28^ Our team, along with other researchers, has concentrated on preclinical and clinical interventions aimed at decreasing mortality associated with CS.^20,29–35^ In numerous scenarios, CS serves as a prevalent and deleterious mechanism. Contemporary approaches to managing CS underscore the importance of multidisciplinary collaboration.^36^ Treatment strategies, such as immunomodulation and organ function support, are generally consistent across various conditions.^37^ However, distinct offender signaling pathways and cytokines vary among different diseases,^38^ leading to tailored treatment approaches. Drawing upon recent advancements in the field globally and our own research, we present a thorough review that examines the role and potential therapeutic interventions of CS in acute and critical illnesses. This review will go through the classic signaling pathways, key immune cells and targeted organ damage associated with CS. It will then delve into the characteristics and management of CS in several critical internal diseases, including FM, ARDS, SIRS, HLH, aGVHD and CAR-T related CRS. Finally, we will discuss potential future directions for improving the management of CS.

## The roles of key signaling pathways and related cytokines involved in CS

### JAK/STAT pathway

**Fig. 2:**
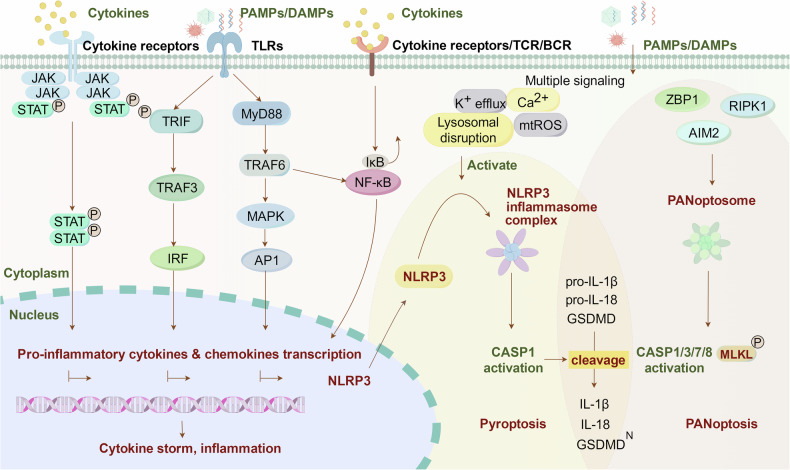
Cytokine signaling pathways. JAK-STAT pathway: cytokines activate the JAK/STAT pathway and trigger the secretion of a variety of pro-inflammatory mediators. TLRs pathways: Stimulated by the PAMPs or DAMPs, TLRs promote cytokine storm mainly via two signaling pathways: the canonical TLRs-MyD88-MAPK pathway and the noncanonical TLRs-TRIF-IRF3 pathway. The TLRs could also regulate the transcription of NF-κB and cause cytokine production. TCR/BCR/NF-κB pathway: cytokines bind to receptors on immune cells and induces NF-κB pathway activation and induce the activation of multiple cytokines. NLRP3 pathway: the activation of NLRP3 requires two signals: The first signal was the activation of inflammatory transcription factor NF-κB, thereby upregulating pro-IL-1β, pro-IL-18, NLRP3, and caspase-1. The second signaling process was NLRP3 induces the formation of super-molecule signaling inflammasome complex by recruiting ASC, leading to IL-1β maturation and secretion of IL-18, as well as to gasdermin D-mediated pyroptosis. PANoptosome pathway: PANoptosome inflammasome complex was assembled and activated by immune disturbance, promoting caspase-dependent and MLKL-dependent PANoptotosis. Abbreviations: JAK Janus Kinase, STAT signal transducer and activator of transcription, TLRs Toll-like receptors, DAMPs damage-associated molecular patterns, PAMPs pathogen-associated molecular patterns, TRIF TIR domain-containing adapter inducing IFN-β, TRAF tumor necrosis factor receptor-associated factor, IRF3 interferon regulatory factor 3, MyD88 myeloid differentiation primary response 88, MAPK mitogen-activated protein kinase, AP-1 activating protein-1, TCR T-cell receptor, BCR B-cell receptor, NF-κB Nuclear Factor kappa B, IκB inhibitor of NF-κB, NLRP3 the NLR family pyrin domain containing 3, NEK7 NIMA-related kinase 7, ASC apoptosis related spot like protein, GSDMD gasdermin D, ZBP1 Z-DNA binding protein 1, AMI2 absent in melanoma 2, MLKL mixed-lineage kinase domain-like pseudokinase. The figure was created with the assistance of FIGDRAW

The JAKs and signal transducers and activators of transcription (STATs) are integral components of a highly conserved signaling pathway that plays a significant role in CS (Fig. 2).^39–43^ This pathway consists of three main structural components: transmembrane receptors, receptor-associated JAKs, and STATs. The JAK family includes four subtypes: JAK1, JAK2, JAK3, and TYK2,^44,45^ while the STAT family consists of seven subtypes: STAT1, STAT2, STAT3, STAT4, STAT5A, STAT5B, and STAT6.^46,47^ Numerous cytokines, including ILs, IFNs, and growth factors, have been demonstrated to participate in JAK/STAT signaling, contributing to essential physiological processes such as cell differentiation, metabolism, hematopoiesis, homeostasis, and immunomodulation.^48,49^ Specifically, IL-6, a multifunctional cytokine, triggers the JAK/STAT3 pathway through classical cis-signaling, trans-signaling, and trans-presentation mechanisms.^50–52^ IL-6 has the ability to interact with the membrane-bound IL-6 receptor (mIL-6R) present on immune cells, as well as with the soluble form of the IL-6 receptor (sIL-6R), forming a complex that triggers the activation of gp130 and subsequently initiates the JAK/STAT3 signaling pathway.^53–55^ This activation cascade of IL-6/IL-6R/JAK/STAT3 results in a systemic hyperinflammatory response, leading to the secretion of various mediators, including IL‑1β, IL‑8, chemokine ligand 2 (CCL2), CCL3, CCL5, granulocyte-macrophage colony-stimulating factor (GM-CSF), and VEGF.^56–60^ Additionally, TNF and IFN-γ are two important pro-inflammatory cytokines that can activate kinases of the JAK family, particularly JAK1. This activation leads to the phosphorylation and activation of STAT proteins, which in turn promotes the expression of inflammation-related genes. This process plays a crucial role in the pathophysiology of CRS.^1,61,62^ The overactivation of the JAK/STAT pathway has been identified as a key factor in the induction of cytokine release and inflammatory disturbances in a variety of diseases, such as HLH,^63–65^ aGVHD,^66^ CAR-T,^67,68^ COVID-19,^69^ and FM-associated CS.^70^

Elevated levels of various cytokines, including IL-1, IL-2, IL-6, IL-10, IL-12, IL-18, TNF, IFN-γ, and GM-CSF, have been detected in the serum of HLH patients. These cytokines primarily activate the JAK/STAT pathways, leading to the excessive production of proinflammatory cytokines and often serving as a negative prognostic indicator.^71,72^ Significantly, IL-2 and IL-12 are pivotal cytokines that induce activation of STAT5 in CD8 T cells.^65^ The JAK/STAT pathway is known to play a critical role in the pathogenesis of aGVHD.^73,74^ It mediates the pro-GVHD effects of natural killer (NK) cells.^75^ Specifically, STAT1 and STAT3 are essential in the regulation of cytokine production, activation, expansion, and the fate of regulatory T cells (Tregs) in aGVHD.^76^

The JAK/STAT pathway plays a significant role in the pathogenesis of CRS associated with CAR-T therapy, with appropriate activation enhancing the antitumor activity of CAR T-cells and overactivation contributing to CRS.^77^ Inhibition of JAK1 has been shown to reduce CRS associated with CAR-T therapy.^67^ In the context of COVID-19, CS is implicated in ARDS and multi-organ failure.^32^ The inhibition JAK demonstrates promising efficacy in the treatment of COVID-19.^78^ The JAK/ STAT pathway plays a significant role in the initiation of viral myocarditis and influences myocardial hypertrophy and heart failure.^79,80^ STAT3 indirectly modulates hypertrophic remodeling and the progression of heart failure.^81,82^ Excessive activation of STAT3 worsens outcomes following myocardial infarction in murine models.^83^ Additionally, STAT3 is crucial for the differentiation of Th17 cells, which has a substantial impact on the development and advancement of myocarditis.^84,85^ These findings emphasize the significant involvement of different cytokines and the JAK/STAT pathway in the pathogenesis of CRS, indicating the potential efficacy of targeting the JAK/STAT pathway as a therapeutic approach for CRS.^69^

### TLRs

Toll-like receptors (TLRs) represent a primitive category of pattern recognition receptors (PRRs) that recognize pathogen-associated molecular patterns (PAMPs). These receptors are present on a variety of immune cells and tissue cells, such as monocytes, macrophages, and dendritic cells (DCs), which serve as detectors of pathogen incursion. The activation of TLRs plays a critical role in the development of infectious diseases and the progression of CS.^86–88^ Upon recognition of PAMPs, TLRs initiate the release of pro-inflammatory cytokines and orchestrate appropriate immune responses to safeguard cells from harm.^89,90^ Activation of TLRs leads to the production of antiviral cytokines such as type I IFNs, IL-1ß, and IL-6, which directly impede viral replication.

Nevertheless, the release of pro-inflammatory factors and cytokines by TLRs may also have deleterious effects (Fig. 2). The excessive production of pro-inflammatory mediators can lead to tissue damage and organ dysfunction. TLRs play a key role in promoting CS through two distinct signaling pathways: the canonical TLRs-MyD88-MAPK pathway, which triggers the transcription of pro-inflammatory factors and cytokines such as TNF, IL-1β, and IL-6; and the noncanonical TLRs-TRIF-IRF3 pathway, which induces the production of type I IFNs (IFN-α and β).

The activation of TLRs plays a significant role in numerous inflammatory diseases and is closely linked to clinical outcomes. Research has demonstrated that TLRs, including TLR3, TLR4, TLR7, and TLR8, among others, contribute to the immune dysregulation observed in cases of COVID-19.^87,91^ A clinical study has indicated the interaction between the spike protein of SARS-CoV-2 and cell surface TLRs, particularly TLR4.^92^ Analysis of samples from endotracheal aspirates, whole blood, and plasma has shown heightened activation of TLR3, TLR4, TLR7, and TLR9 in critically ill COVID-19 patients.^93^ The utilization of nucleic acid-binding microfibers in treatment has shown potential in mitigating the over-activation of TLRs and the subsequent nuclear factor kappa B (NF-κB) pathway by removing damage-associated molecular patterns (DAMPs)/PAMPs in affected patients.^93^

TLRs have been implicated in the pathogenesis of viral myocarditis, with genetic variations in TLRs influencing susceptibility to the condition. In a cohort of patients with biopsy-proven enteroviral myocarditis, the presence of a single nucleotide polymorphism (SNP) in the TLR3 gene was observed in 52.63% of cases, with 21.05% being homozygous for the SNP. In contrast, the homozygosity rate of this SNP was found to be only 4% in the control population.^94^ Furthermore, there is evidence suggesting that susceptibility to myocardial inflammation varies depending on TLR4 SNPs.^95^ In the classical mouse model of coxsackievirus B3 (CVB3)-induced viral myocarditis, CVB3 infection leads to the upregulation of all TLRs,^96^ which in turn triggers cytokine production and immune cell recruitment to the myocardium. The cardiac infiltrating immune cells, in conjunction with damaged cardiomyocytes, secrete a significant amount of cytokines and chemokines, such as IL-1, IL-6, TNF, and IFNs, which contribute to additional tissue injury and cytokine release, establishing a detrimental positive feedback loop.^97–100^ Improper activation of TLRs may lead to the development of autoimmune reactions. For instance, due to structural similarities with specific PAMPs, exposure of cardiac myosin can directly stimulate TLR2 and TLR8, initiating downstream signaling pathways.^101,102^

### NETs

Neutrophil extracellular traps (NETs) are extracellular reticular structures formed by neutrophils. NETs contain various components such as neutrophil elastase, myeloperoxidase, cathepsin G, histone, and DNA. They have a strong capacity to capture pathogens and limit the dissemination of infection. Inflammatory stimulation may induce the production of NETs through a process of reticular proliferation by neutrophils, referred to as NETosis (a new programmed cell death). The contents of NETs could enhance the pro-inflammatory activity of neutrophils by promoting the release of IL-8. Additionally, NETs facilitate the activation of CD4^+^ T cells and the phagocytic function of macrophages through inflammasome signaling.^103^ In the context of atherosclerosis, NETs increase macrophage expression of IL-6 and pro-IL-1β via the TLR2 and TLR4 pathways. The elevation of pro-inflammatory cytokines facilitates the differentiation of Th17 cells and the recruitment of myeloid cells.^104^

NETs potentially exacerbate SARS-CoV-2-induced CS and macrophage activation syndrome (MAS).^105^ In the context of COVID-19, the excessive formation of NETs is linked to the onset of acute lung injury (ALI), ARDS, and an increased risk of immunothrombosis.^106^ NETs exacerbate the progression of viral myocarditis. The inhibition of NETs improved the outcome of experimental autoimmune myocarditis. One identified mechanism that governs the release of NETs and NETosis is the midkine–low-density lipoprotein receptor related protein 1 axis.^107^

### NLRP3 inflammasome

The NLR family pyrin domain containing 3 (NLRP3) inflammasome is a complex of multimeric cytosolic proteins that forms in response to cellular stimuli. Activation of the NLRP3 inflammasome leads to the activation of caspase-1, which in turn promotes the maturation of IL-1β, IL-18, and gasdermin D (GSDMD, Fig. 2).^108,109^ Activation of the NLRP3 inflammasome requires two signals, the first of which involves the activation of NF-κB through PRRs. NF-κB translocates to the cell nucleus to initiate the transcription of caspase-1, NLRP3, pro-IL-1β, and pro-IL-18. Subsequently, cellular stress signals stimulate the assembly and activation of the NLRP3 inflammasome complex. The NLRP3 inflammasome then facilitates the dimerization and activation of caspase-1, leading to the cleavage of pro-IL-1β, pro-IL-18, and GSDMD into their active forms.^110^ IL-1β facilitates the recruitment of neutrophils and T cells to the site of infection, resulting in the release of secondary wave cytokines such as IL-6 and TNF by epithelial and endothelial cells. Elevated levels of IL-18 stimulate the production of IFN-γ by T cells and NK cells.^111^ Additionally, GSDMD serves as a pro-inflammatory mediator that triggers pyroptotic cell death. Furthermore, the binding of IL-1β to IL-1 receptor 1 (IL-1R1) and IL-18 to IL-18 receptor (IL-18R) activates the NF-κB signaling pathway, creating a positive feedback loop that amplifies the inflammatory response.^112^

Continuous activation of the NLRP3 inflammasome is associated with the pathogenesis of several inflammatory disorders, including Alzheimer’s disease, asthma and allergic airway inflammation, diabetes, inflammatory bowel disease, atherosclerosis, gouty arthritis and so on.^113^ In addition, gain of function mutation of NLRP3 leads to a group of autoinflammatory disorders known as cryopyrin associated periodic syndromes (CAPS), including neonatal-onset multisystem inflammatory disease, and familial cold autoinflammatory syndrome and Muckle-Wells syndrome.^114^ There have been more than 200 mutations in the NLRP3 gene to be reported in association with CAPS in the INFEVER database.^115^ These mutations lead to spontaneous inflammasome formation and IL-1β, IL-18 production together with cell pyroptosis in the absence of the stress signal. The CAPS patients are characterized by fever, blood neutrophilia and tissue specific inflammation in the skin, joints and conjunctiva.^114^ And mouse with CAPS­ associated NLRP3 variants display systemic, lethal inflammation.^116^

The activation of the NLRP3 inflammasome has been associated with various inflammatory diseases. In the context of viral myocarditis, CVB3 infection has been shown to trigger inflammasome activation both in vivo and in vitro.^117,118^ Similarly, in viral pneumonia caused by SARS-CoV and MERS-CoV infections, the NLRP3 inflammasome plays a critical role in the hyperinflammatory immune responses.^119,120^ Furthermore, in the case of COVID-19, activation of the NLRP3 inflammasome not only contributes to severe respiratory complications but also leads to the development of neurological syndromes.^121^

## The role of cell death and immune cell activation in CS

### Cell death in CS

CS is a pathological state caused by the excessive response of the immune system and the release of large amounts of cytokines, this state can lead to a variety of cell death pathways, including but not limited to necroptosis, apoptosis, pyroptosis and PANoptosis. Necroptosis is a type of programmed necrosis involved in immune response to viral infections, and severe inflammatory injury.^122^ Caspase-8 inhibition was found to be sufficient to decrease necroptosis and release the anti-inflammatory cytokine IL-10, which is involved in the immunosuppressive stage of sepsis.^123^ Apoptosis plays a pivotal role in pathogen elimination and maintaining homeostasis. The SARS-CoV-2 infection triggered caspase-8-dependent apoptosis and lead to the lung damage in the COVID-19 patients.^124^ Pyroptosis is a proinflammatory form of programmed cell death, acting as a host defense mechanism against infections.^125,126^ SARS-CoV-2-encoded coronavirus products act to modulate various key components in the pyroptosis pathways, including inflammasomes, caspases and gasdermins.^127^

**Fig. 3:**
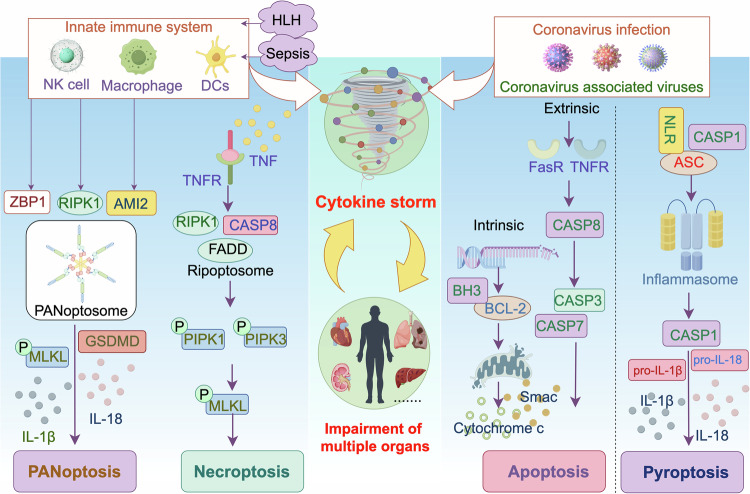
Cell death in cytokine storm. Robust release of cytokines has been suggested to correlate with lung injury and multiple organ failure. This state can activate a variety of cell death pathways, including but not limited to PANoptosis, necroptosis, apoptosis, and pyroptosis. Macrophages infected in conditions such as sepsis and HLH can trigger cytokine storm, during which the synergistic stimulation by inflammatory factors TNF and IFN-γ induces PANoptosis in macrophages. Multiple inflammatory cytokines are produced during β-coronavirus infection, HLH, and sepsis. ZBP1, AIM2, and RIPK1 are common triggers of PANoptosome. Cytokines and caspases, including caspase-8, were involved in the immunoregulation stage of sepsis. The coronavirus infection triggered caspase-8-dependent apoptosis and lead to lung damage. SARS-CoV-2-encoded coronavirus products could modulate various key components in the pyroptosis pathways and leading to cytokine storm syndrome. Abbreviations: HLH hemophagocytic lymphohistiocytosis, NK cell natural killer cell, DC dendritic cell, TNFR tumor necrosis factor receptor, ISGs interferon-stimulated genes, IFN interferon, FADD Fas-associated death domain, NLR NOD-like receptor, ASC apoptosis related spot like protein, GSDMD gasdermin D, RIPK receptor interacting protein kinases, MLKL mixed-lineage kinase domain-like pseudokinase, ZBP1 Z-DNA binding protein 1, AMI2 Absent in Melanoma 2, CASP caspase, BCL-2 B-cell lymphoma-2. The figure was created with the assistance of FIGDRAW

PANoptosis is a distinct innate immune inflammatory regulated cell death (RCD) pathway that is governed by the PANoptosome complex, which incorporates elements from other RCD pathways.^128^ The occurrence of PANoptosis is related to many diseases, such as infectious diseases, cancer, cardiovascular diseases and autoimmune diseases. Different signals activate specific sensory proteins, initiating the assembly of distinct PANoptosome complexes. ZBP1, AIM2, and RIPK1 are common PANoptosome triggers, which can be activated by different pathogens or stimuli and trigger a series of biological responses. These reactions include apoptosis, CS, etc. For example, ZBP1 plays an important role in diseases such as influenza virus infection and CoV infection during IFN treatment; AIM2 is involved in the pathological processes of diseases such as herpes virus type 1 infection and bacterial infection. IFN signaling plays multiple roles during viral infections.^129^ Among the various inflammatory cytokines produced by innate immune cells in response to SARS-CoV-2 infection, it has been identified that the co-production of TNF and IFN-γ uniquely triggers PANoptosis.^62^ During SARS-CoV-2 infection, Karki et al. found that only the combination of TNF and IFN-γ induced a form of inflammatory cell death characterized by PANoptosis. Treatment with neutralizing antibodies against TNF and IFN-γ protected mice from mortality associated with SARS-CoV-2 infection, sepsis, HLH, and cytokine shock.^62^ A strong release of cytokines has been proposed to be associated with lung injury and dysfunction of multiple organs (Fig. 3).^130^

### T cells

T cell activation is an important part of inflammatory response and CS induction. T cells can be classified into two main subtypes: CD4^+^ T helper cells (Th) and CD8^+^ cytotoxic T lymphocytes (CTL).^131^ While CD4^+^ T cells are mainly engaged in immune modulation, CD8^+^ CTLs are direct effector cells of proinflammatory factor production and tissue damage.^132,133^

CD8^+^ CTLs selectively target infected or malignant cells, leading to their demise through the secretion of pro-inflammatory cytokines, interaction with the Fas ligand receptor, and release of cytolytic granules.^134,135^ CTL dysfunction could lead to CRS. One prominent factor is perforin-mediated cytolysis.^136^ Perforin is contained in cytolytic granules released by CTLs. It forms a channel for cytotoxic mediators to enter the targeted cells and lead to cytolysis.^137^ Several gene products are engaged in the process of granule formation and perforin fusing to targeted cells. Genetic defects in these genes result in CTL inability to kill the targeted cells and sustained DAMPs/PAMPs presence.^136^ The prolonged crosstalk between CTLs and antigen-presenting cells (APCs) resulted in substantial proinflammatory cytokines production, which is the key mechanism of CS in primary and secondary HLH.^138,139^

The cell-targeting and cytotoxic capabilities of CD8^+^ T cells have been extensively studied for potential therapeutic applications. One notable example is adoptive cell therapy, in which CD8^+^ T cells obtained from patients are expanded and activated ex vivo before being reintroduced into the patient.^140,141^ Significant advancements have been achieved in CAR-T therapy, a treatment modality in which genetically modified CD8^+^ T cells are reinfused into patients to specifically target and combat cancer cells.^142^

### NK cells

NK cells primarily exert anti-viral and anti-tumor responses and produce pro-inflammatory cytokines.^143,144^ However, dysfunction of NK cells lead to inability to eliminate the infected or malignant cells, lead to sustained immune activation and CS. One mechanism is mediated by perforin deficiency, similar as above described in CD8^+^ CTLs.^136^ Additionally, NK cells exhibit plasticity in cytokine production based on the surrounding inflammatory environment. Upon encountering tumor ligands and intracellular pathogens, NK cells secrete Th1-type cytokines like IFN-γ, TNF, and GM-CSF, which in turn stimulate the activation of T cells, macrophages, neutrophils, and DCs. Additionally, NK cells release chemokines such as MIP-1α, MIP-1β, CCL5, lymphotoxin, and IL-8 to attract myeloid cells and effector lymphocytes to the inflamed tissues.^145^ The cytokines IL-2 and IL-15 play crucial roles in activating NK cells, with recombinant IL-15 expanding NK cell populations and promoting tumor regression while reducing metastasis.^146,147^

### Macrophages

Overactivation of macrophages directly lead to inflammation amplification and CS, as represented by the MAS.^148^ Macrophages are commonly classified into M1 and M2 subtypes based on their activation patterns and inflammatory capabilities. M1 macrophages are primarily activated by T cell response, specifically CD4^+^ Th1 cells, and exhibit robust antigen-presenting and pro-inflammatory functions.^149^ These cells serve as the primary effector cells of the immune system for pathogen elimination, being activated by IFN-γ and TNF, and releasing inflammatory cytokines including IL-1β, IL-6, IL-12, IL-15, IL-23, TNF, and MIP-1. M1 macrophages have the ability to recruit granulocytes, NK cells, Th cells, and other macrophages to the site of infection through the secretion of inflammatory chemokines, including the monocyte chemoattractant protein 1 (MCP-1), CXCL10, CCL2, CCL5, CXCL8, and CXCL9. Additionally, they facilitate the differentiation of CD4^+^ T cells into Th1 and Th17 cells, which play a crucial role in eliminating invasive pathogens. These mechanisms collectively contribute to the potent anti-infective and inflammatory functions of M1 macrophages.

M2 macrophages, known for their anti-inflammatory properties, are the predominant macrophage subtype involved inflammation resolution and tissue fibrotic healing.^150,151^ The M2 macrophages can be further classified into M2a, M2b, M2c and M2d subsets.^152^ The M2a macrophages are most well-studied. They are regulated by IL-4 and IL-10 and have anti-inflammatory potential. At the infected or injured sites, the macrophages are motivated by sensing phosphatidylserine on the surface of apoptotic cells.^153^ And IL-4R and IL-13R on the macrophages are activated by type 2 cytokines IL-4 and IL-13.^152^ The collaboration of apoptotic cell sensing and IL-4/IL-13 signaling potentiate macrophages polarizing into M2a phenotype and resolve inflammation.^154^ Defects in IL-4 signaling inhibits the DNA repairing function of macrophage and renders macrophage into a proinflammatory state, promoting inflammation expansion and organismal aging.^155^ The M2b macrophages, also known as regulatory macrophages are activated by immune complexes and TLR ligands.^156^ They produce both pro- and anti-inflammatory cytokines to exert immune regulatory function. The M2c macrophages are activated by glucocorticoids or IL-10 and promote tissue regeneration.^157,158^ The M2d macrophages, also known as TAMs, are activated by TLR ligands and A2 adenosine receptor agonists, and promote tumor progression and metastasis.^159^

Key anti-inflammatory cytokines produced by M2 macrophages include IL-10 and transforming growth factor β (TGF-β), with IL-10 directly inhibiting APCs and interfering with the differentiation of Th1 and Th17 cells by suppressing IL-12 and IL-23 synthesis.^160^ Excessive polarization of M2 macrophages leads to impaired pathogen clearance and compromised T cell regulatory function. This impairment in T cell function subsequently diminishes the bactericidal capacity of macrophages and the production of antibodies by B cells. Moreover, the inability to effectively clear pathogens perpetuates their proliferation and sustains immune system stimulation, leading to uncontrolled secretion of inflammatory factors and ultimately triggering CS.^161–163^

### Neutrophils

Neutrophils are the first responders to sites of injury, infection, and inflammation through chemotaxis.^164,165^ Upon recognition of PAMPs or DAMPs, or in response to inflammatory signals, neutrophils initiate an immune response by recruiting and activating other leukocytes, as well as signaling the bone marrow to produce and mature more neutrophils.^166^ Neutrophils employ three main mechanisms to eliminate pathogens: phagocytosis, NETosis, and degranulation.^167,168^ Phagocytosis involves the engulfment, internalization, and degradation of pathogens by neutrophils, while NETosis is the extracellular trapping process of pathogens. Degranulation is the process by which neutrophils release various cytokines from their granules, including pro-inflammatory cytokines such as IL-1α, IL-1β, IL-6, IL-16, IL-18, and MIF.^169,170^ Neutrophils not only release inflammatory mediators but also play a role in regulating the function of other immune cells, particularly monocytes/macrophages.^171,172^ For instance, the interaction between macrophages and neutrophils involves the secretion of azurocidin by neutrophils, which in turn enhances phagocytic activity and the release of pro-inflammatory cytokines such as TNF and IFN-γ.^173^ Neutrophils also release CXCL10 to promote macrophage proliferation through CXCR3 signaling.^174^ Additionally, neutrophils are eliminated through macrophage-mediated efferocytosis in the later stages of inflammation.^175,176^

Neutrophils are essential in the pathogenesis of inflammatory diseases. In viral myocarditis, early neutrophil ablation resulted in reduced monocyte influx into the myocardium.^171,177^ Furthermore, inhibiting NETs formation has significantly reduced inflammation and maintained systolic function in mice with myocarditis.^107^ A significant presence of neutrophils was observed in the lungs under normal physiological conditions.^178^ During the initial phase of COVID-19, neutrophils become activated and migrate to the lungs to combat the SARS-CoV-2 virus.^179^ Nevertheless, excessive activation of neutrophils is linked to the development of severe CS in COVID-19 patients.^180^ Numerous clinical investigations have demonstrated a correlation between elevated levels of circulating neutrophils and impaired oxygenation in individuals with COVID-19.^181,182^ Additionally, low-density neutrophils, which are particularly prone to undergoing NETosis, are closely associated with the formation of microthrombi in blood vessels and the development of ARDS in COVID-19 patients.^183^

### B cells

The primary role of B cells is the production of antibodies, as well as involvement in antigen delivery and regulation of T cell activity. In the context of CAR T-cell therapy, a lack of antibody production by B cells leads to an inability to effectively clear pathogens, resulting in the repeated triggering of the inflammatory cascade by PAMPs and ultimately leading to CS. In addition, the antibodies generated by B cells against viral antigens may also cross-react with autoantigens, potentially leading to autoimmune responses.^184,185^

### Mast cells

Mast cells, recognized for their involvement in allergic reactions and parasitic infections, also play a significant role in inflammation. Studies have shown that mast cells are the primary source of TNF release in the myocardium, with cardiac mast cells secreting elevated levels of pro-inflammatory cytokines such as TNF, IL-1β, and IL-10 during CVB3 infection.^186–188^ The accumulation of mast cells has been linked to an increase in CCL2-mediated Ly6C^high^ macrophages infiltrating the heart, exacerbating cardiac dysfunction and fibrosis in cases of viral myocarditis.^186^ The early stem cell factor derived from resident cardiac fibroblasts stimulates mast cell accumulation and the secretion of pro-inflammatory cytokines. These cytokines, in turn, activate fibroblasts to express TGF-β, deposit collagen, and produce additional cytokines. Mast cells collectively contribute to the pathogenesis of viral myocarditis by exacerbating inflammation and fibrosis.

### Eosinophils

Eosinophils are essential for maintaining immune homeostasis through the synthesis of a variety of toxic granule proteins.^189^ When stimulated, eosinophils release these proteins, which include major basic proteins, peroxidase, neurotoxin, and cytokines.^190^ Eosinophils exhibit anti-infective properties against parasites, bacteria, and viruses, while also contributing to the pathogenesis of inflammatory diseases such as myocarditis, asthma, and hypereosinophilic syndromes. In hypereosinophilic diseases, the persistent activation of eosinophils results in the release of granule proteins and chemical mediators, leading to tissue damage.^191–193^ Eosinophil proliferation, maturation, and recruitment are regulated by cytokines such as IL-5, IL-4, and IL-13. Glucocorticoids have traditionally been utilized for the treatment of eosinophilic diseases by nonspecifically attenuating eosinophils. Recently, several novel biologic therapies have been approved for clinical use to specifically target factors involved in eosinophil maturation, including IL-5, the IL-5 receptor, or IL-4/IL-13.^194^

## The targeted organ damage involved in CS

CS often results in multi-organ failure, including heart, lung, and kidney failure/damage and so on. Though organ dysfunction is considered secondary damage rather than the underlying pathophysiology, they reflect the severity of CS and are the direct cause of death. Understanding how CS impact these organs help us recognize CS earlier and provide instant treatment.

### Vascular endothelium

In CS, the vascular system is primarily affected by increased vascular permeability and endothelial dysfunction. This phenomenon is primarily driven by the excessive release of cytokines such as IL-1, IL-6, and TNF. These cytokines activate and injure endothelial cells, leading to increased vascular permeability. As a result, this process leads to tissue edema and fluid accumulation.^1,195^ Additionally, the overproduction of cytokines may impair endothelial function, manifested by endothelial cell contraction and impaired vasodilation, which in turn causes hypotension and inadequate tissue perfusion.^196^ Cytokine-induced vasodilation can further reduce blood pressure, and in severe cases, may result in insufficient organ perfusion or even shock. Moreover, cytokines like IL-6, IL-1β, and TNF can activate the coagulation system, promoting platelet aggregation and the activation of coagulation factors, thus elevating the risk of thrombosis.^197^ The activation of the complement system and the formation of NETs can further exacerbate vascular damage and thrombus formation.^198^ Activation of the complement system contributes to endothelial injury and platelet activation, while NETs facilitate platelet aggregation and coagulation factor activation, promoting thrombosis.^199,200^

### Heart

Cardiac damage resulting from CRS primarily presents as fulminant myocarditis with rapid hemodynamic deterioration and severe arrhythmias. The cytokines induce increased capillary permeability, resulting in fluid leakage into the myocardial tissue. Echocardiography, MRI, and endomyocardial biopsy (EMB) demonstrate significant myocardial edema. Additionally, the cytokines directly impair cardiac contractility, leading to cardiogenic shock and potentially multiple organ dysfunction syndrome due to inadequate tissue perfusion. For instance, the pro-inflammatory cytokine IL-1 has been shown to exert a negative inotropic effect, directly reducing myocardial contractility.^201^ Blocking the IL-1 receptor with anakinra has been shown to effectively improve cardiac contractility and outcomes in patients with FM.^202–204^ Furthermore, cytokines have been found to inhibit mitochondrial function, leading to impaired energy production.^205^ This results in the production of excessive reactive oxygen species (ROS), causing oxidative stress and cell death in the myocardium.^206,207^ Additionally, the release of self-antigens from damaged cells can exacerbate inflammatory disturbances and further stimulate cytokine release. Hence, the cardiac contractile function is significantly diminished due to inadequate energy provision, tissue edema, and cellular damage.

In addition to contractility impairment, CS also disrupts the coordination of electrical conduction and synchronization of cardiac contractions, leading to arrhythmias. Atrial fibrillation, tachycardia, bradycardia, and refractory ventricular fibrillation are common arrhythmias observed in patients with CS. In the acute phase of FM, it is widely acknowledged that CS may induce or exacerbate arrhythmia through three primary mechanisms.^208,209^ Firstly, cytokines directly disrupt Ca2^+^ homeostasis, with various cytokines including TNF, IL-1β, and IL-6 impacting Ca2^+^ handling through alterations to ryanodine receptors and the L-type voltage-gated calcium channel Cav1.2.^210^ Given the critical role of Ca2^+^ in action potential generation and excitation-contraction coupling, disturbances in Ca2^+^ signaling are known to promote arrhythmia. Secondly, cytokines have been shown to cause direct damage to the plasma membrane of cardiomyocytes by inducing membrane lysis and reducing cell-to-cell junctions, resulting in electrical instability and impaired conduction. In murine myocarditis, CVB3 has been found to decrease the expression of connexins in the myocardium and disrupt gap junction function.^211^ Thirdly, the infiltration of inflammatory cells and tissue edema have been observed. Lower potential at focal sites with significant lymphocyte infiltration has been demonstrated by electroanatomic voltage mapping on endomyocardial biopsies.^212^ Arrhythmia is a prevalent clinical presentation of FM and is linked to a poor prognosis.^213^ In addition to FM, the cytokine cascades of various inflammatory conditions can also lead to cardiac damage. For instance, CS is closely correlated with cardiac injury and the development of cardiovascular events in both CAR-T therapy and COVID-19.^98,99^ Cardiovascular manifestations of CS encompass myocardial injury, myocarditis, arrhythmias, ischemic heart disease, and heart failure.^96,97^

### Lung

Numerous inflammatory cytokines have the potential to target the lungs, resulting in alveolar collapse, reduced lung compliance, heightened pulmonary vascular resistance, and disruptions in gas exchange. Lung injury is a prevalent occurrence in CS, stemming from various sources including underlying diseases like FM and respiratory infections, as well as treatment-related complications such as CAR-T cell therapy-induced inflammation and GVHD following HSCT. Additionally, pathogens can trigger ARDS by interacting with receptors on alveolar epithelial cells. The accumulation of cytokines in the lung parenchyma is influenced by the abundance of small blood vessels. These cytokines, which stimulate inflammatory responses, contribute to the structural and functional impairment of lung tissue.^214^ This inflammatory cascade results in hypoxia, diminished sodium pump activity in alveolar epithelial cells, disturbances in cellular metabolism, and ultimately exacerbates lung injury. The release of NF-κB further amplifies the inflammatory response, ultimately leading to the development of ARDS as lung function deteriorates.^215^

IL-6 is a critical factor in the pathogenesis of lung injury. IL-6 can induce immune cell accumulation in the lungs, trigger the release of free radicals and proteases from immune cells, leading to injury of lung epithelial and capillary endothelial cells. IL-6 also promote alveolar cell pyroptosis through synergistic interactions with inflammatory vesicle complexes.^216^ Additionally, IL-6 has been shown to decrease fibronectin production, resulting in weakened cell-cell connections.^217^ As alveolar and vascular epithelial cells undergo edema and pyroptosis, the permeability of the respiratory membrane is heightened. Furthermore, IL-6 plays a role in the differentiation and maturation of Th 17 cells, which in turn produce cytokines such as IL-17 and IL-22, thereby promoting the production of inflammatory cytokines by various cell types including fibroblasts, DCs, macrophages, and endothelial cells.^218^

### Bone marrow

In CS, excessive cytokine release leads to widespread disruption of bone marrow function. Cytokines can suppress bone marrow function, leading to decreased production of key blood cells such as erythrocytes, leukocytes, and platelets.^219,220^ This disruption can be divided into two main aspects: hematopoietic stem cell (HSC) dysfunction and impairment of the bone marrow microenvironment.

Inflammatory cytokines, such as TNF, IFN-γ, and IL-6, impair HSC self-renewal and differentiation, leading to accelerated aging and depletion of the HSC pool.^221,222^ Increased TNF induces IL-27Ra via the ERK-ETS1 pathway, promoting inflammation and further compromising HSC function.^223^ IFN-γ also negatively impacts HSC self-renewal.^224^ These persistent inflammatory exposure results in progressive and irreversible hematopoietic suppression, manifesting as anemia, leukopenia, and thrombocytopenia.^225^ Prolonged cytokine exposure exhausts the HSC pool, leading to bone marrow failure and heightened infection risk.^219,226^

Beyond directly affecting HSCs, cytokine-driven inflammation disrupts the bone marrow microenvironment,^227^ where stromal cells play a crucial role in supporting hematopoiesis. Increasing evidence identifies IL-1β as a central mediator of this microenvironmental inflammation, driving hematopoietic aging and altering the function of the supportive niche.^228^ Notably, these microenvironmental impairments are not reverted by systemic rejuvenation interventions, underscoring their irreversible nature. This inflammatory disruption is also evident in pathological conditions such as aGVHD, where overactivation of T cells and dysregulated cytokine production cause severe damage to the bone marrow niche, resulting in significant bone marrow suppression.^229–231^ Similarly, in HLH, activated macrophages driven by TNF and IFN-γ promote the phagocytosis of hematopoietic cells, further contributing to bone marrow failure.^232^

### Kidney

Renal damage resulting from CS primarily presents as acute renal dysfunction or injury leading to renal failure. Patients may exhibit symptoms including azotemia, oliguria, and anuria.^233^ The pathogenesis of this injury may involve immune cell recruitment, microthrombosis, and dysfunction of other organs. IL-6 plays a role in promoting the differentiation and maturation of Th17 cells, which can secrete IL-17 and TNF, working together to decrease vascular endothelial nitric oxide (NO) production and increase vasoconstriction. The regulatory role of IFN-γ in the production of renal localized angiotensinogen leads to overactivation of the AngII-renin-angiotensin aldosterone system, resulting in increased aldosterone production, water and sodium reabsorption, and ultimately hypertension.^234–238^ This cascade of events can cause damage to renal capillary endothelial cells and contribute to the development of renal atherosclerosis. Additionally, T cells attracted by cytokine chemotaxis deposit in renal capillaries, infiltrate the capillary outer membrane and peripheral fat, and generate ROS, ultimately leading to renal injury and fibrosis.

Severe hypercoagulability can lead to disseminated intravascular coagulation (DIC), with the formation of renal microthrombi in the capillary network contributing to the development of renal microfibrosis, acute tubular necrosis, and impairment of cortical function.^239^ In advanced stages of CS, decreased cardiac output due to cardiac insufficiency may result in renal hypoperfusion.^240^ Furthermore, hypoxia from lung injury and hepatorenal syndrome from hepatic insufficiency can also contribute to renal injury. CS is closely related to the complement system in kidney injury. CS related innate immunity dysregulated response is focused on IFN and complement dysfunction.^241^ Renal tubular epithelial cells, glomerular endothelial cells, and interstitial cells can all synthesize and secrete complement components and bind to local immune cells in the kidney and the activated complement receptor on the endothelial cell membrane. The complement dysfunction is involved in acute lesions such as glomerulonephritis, acute kidney injury and acute graft rejection, as well as chronic diseases such as diabetic nephropathy, nephrotic syndrome and chronic renal fibrosis.^242^

### Liver and gastrointestinal tract

Liver damage resulting from CS encompasses hepatomegaly, liver injury, and potentially fatal liver failure. Patients may exhibit symptoms including elevated aminotransferases, hyperbilirubinemia, hypoalbuminemia, and cholestasis.^233^ Gastrointestinal tract damage may manifest as nausea, vomiting, abdominal pain, diarrhea, ascites, and colitis. The cytokines IL-1, IL-6, TNF, and IFN are all implicated in contributing to liver damage. IL-6 interacts with sIL-6R to stimulate the production of acute phase proteins, including serum amyloid A, C-reactive protein (CRP), and fibronectin.^243^ The accumulation of amyloid may contribute to the development of hepatic amyloidosis, potentially leading to hepatic failure, as well as impacting renal and gastrointestinal function, ultimately resulting in multi-organ failure.^244^ Additionally, acute-phase proteins and fibronectin have the potential to activate the complement system and initiate the coagulation cascade, resulting in a sustained hypercoagulable state within the circulatory system. The interaction between immune cell infiltration, the complement system, and procoagulant pathways contributes to the development of microthrombosis.^245–247^ Hepatic dysfunction disrupts the balance between coagulation and anticoagulation, potentially leading to DIC in severe cases. Activation of inflammatory vesicles triggers the production of IL-1β by IL-1, leading to hepatocyte pyropoiesis and the activation of other cytokines in the liver. This positive feed-forward response exacerbates inflammatory damage, with inflammasomes and IL-1 playing significant roles in hepatocellular injury and liver failure.^248^ Moreover, the liver contains a high concentration of NK cells, which, when over-activated, can trigger the STAT1 signaling pathway in an IFN-γ-dependent manner, thereby hindering the proliferation of hepatocytes and impeding hepatic regeneration.^249^ Additionally, TNF plays a dual role in liver function, acting through the NF-κB pathway to prevent cell death and through the ROS-JNK pathway to induce apoptosis and necrosis in hepatocytes.^250^

### Central nervous system

CS in central nervous system (CNS) is a common occurrence and has been linked to neurologic dysfunction in various conditions such as sepsis-associated encephalopathy, cerebral malaria, and CNS infections.^251–253^ In patients undergoing treatment with cellular therapies and other immunotherapies for CNS tumors, CS can contribute to immune effector cell-associated neurotoxicity syndrome or tumor inflammation-associated neurotoxicity.^251,254^ Elevated levels of cytokines in the cerebrospinal fluid have been associated with a poorer prognosis.^255,256^ Patients with CNS involvement of CS may exhibit symptoms such as cerebral edema, cognitive impairment, dysarthria, headache, hallucinations, aphasia, hemiparesis, cranial nerve dysfunction, seizures, and lethargy.^233,251^ Brain CS could lead to vascular leakage, complement activation, and coagulation abnormalities, which would predispose patients to an increased risk of stroke and ischemic necrosis of brain tissue.^257,258^ Endothelial injury induced by elevated levels of IL-6 and TNF results in enhanced blood-brain barrier permeability, facilitating the entry of various cytokines from the bloodstream into brain parenchyma.^259,260^ Activation of microglial cells and astrocytes within the CNS has the potential to release a diverse array of inflammatory factors, leading to detrimental effects on neurons and glial cells, ultimately manifesting as neurological symptoms.^261,262^ The presence of IFN-γ and TNF may further intensify these effects and worsen the neurological manifestations.^263^ Moreover, viruses are more prone to infiltrate and directly damage brain tissue. While CAR-T cells generally do not directly inflict damage on brain tissue, an exception exists in B-cell maturation antigen (BCMA) associated CAR-T therapies, which have been specifically linked to Parkinsonian phenomena.^264^ The etiology of immune effector cell–associated neurotoxicity syndrome (ICANS) remains unclear.

IL-1 and the IL-1 receptors are expressed in the brain. IL-1 expression in the brain is low at baseline. However, under various pathological conditions IL-1 has been shown to exacerbate neurodegeneration associated with multisystem inflammatory disease.^265^ The IL-1 receptor antagonist (IL-1Ra, anakinra), with its CNS penetration capacity, exerts protective effects against CNS CS.^266^

## Overcoming strategies of CS in different diseases

There are numerous etiological factors contributing to CS, including underlying diseases such as FM, viral pneumonia, severe infection, and HLH. Additionally, CS can arise as a complication of certain treatments, such as CAR-T therapy and GVHD following allo-HSCT. Our research team has accumulated valuable preclinical data and clinical experience that contribute to efforts in reducing mortality associated with CS in these conditions.

### Fulminant myocarditis

Myocarditis, an inflammatory condition affecting the cardiac muscle, can be caused by various factors such as infections, immunotherapy toxicity, and autoimmune diseases, with viral infection being the predominant etiology.^209,267,268^ FM represents the most severe form of the disease, characterized by rapid clinical deterioration leading to hemodynamic instability, circulatory dysfunction, and potentially life-threatening arrhythmias.^209,267^ Despite its rarity, FM carries a high mortality rate and significant morbidity, particularly among younger individuals.^269^ In China, an estimated 30,000 to 50,000 cases of FM are reported annually.^270^

Owing to the nonspecific prodromal symptoms and its extremely rapid progressive nature, most of our knowledge about FM was obtained from postmortem examination.^271,272^ In recent years, with the help of improved treatment and EMB technique, deep insight into FM has been obtained.^273–278^ The detrimental role of CS in FM has been identified based on evidence from three aspects. First, multiple inflammatory markers and cytokines significantly increase in FM, including IL-1, IL-10, soluble suppression of tumorigenicity-2 (sST2), TNF, IFN-γ, MIP-1α, MIP-2, and so on.^34,279–281^ Second, massive pro-inflammatory immune cells infiltrated into the myocardium,^20,282–285^ accompanied by multiple organ dysfunction such as hepatic failure, renal failure, and respiratory failure.^272^ Third, immunomodulatory therapy including glucocorticoids and intravenous immunoglobulin (IVIG) or cytokine blockade is effective in treating FM.^202,204,286–288^ We have previously performed a comprehensive profiling of 122 inflammatory cytokines in an FM cohort. Significant alterations in 39 cytokines were detected in the FM samples compared to matched controls, supporting a state of CS.^34^ Additionally, analysis of EMB samples revealed substantial infiltration of immune cells in the degenerative or necrotic myocardium of individuals with FM, including T cells, macrophages, and eosinophils, despite the release of cytokines^283,289,290^ In a comprehensive multi-viral PCR test targeting 178 human viral genomes, including those associated with SARS-CoV and myocarditis-inducing viruses, no viral genome was detected in the FM specimens. This finding underscores the importance of the immune response in the pathogenesis of FM, rather than direct viral-induced damage to the heart.^290^

### Pathogenesis of CS in FM

Viral infection is identified as the primary etiology of FM.^291–293^ Innate immunity plays a predominant role in viral FM, with TLRs, NETs, and inflammasomes being pivotal in signaling pathways, immune cell activation, and cytokine secretion.^95,107,171,294–302^ Neutrophils are promptly recruited to the myocardium in acute myocarditis, serving as one of the initial immune cells to respond to the condition.^171,303^ Carai et al. further demonstrated significant neutrophil infiltration and the presence of NETs in the hearts of mice with acute CVB3 myocarditis.^171^ In our recent investigation of FM, we observed a distinct developmental trajectory of neutrophils upon their migration to the heart, where they continuously recruited peripheral neutrophils through the Cxcl2/Cxcl3-Cxcr2 axis, leading to acute neutrophil accumulation in the myocardium. Furthermore, these cardiac-differentiated neutrophils recruited and activated pro-inflammatory macrophages, exacerbating cardiac CS. The inhibition of the autoregulatory recruitment mechanism of neutrophils significantly mitigated FM in mice.^20^ The early depletion of neutrophils using anti-Ly6G antibodies reduced monocyte influx into the heart, inhibit pro-inflammatory macrophage differentiation, and prevent the upregulation of chemokines CXCL1 and CXCL2 (analogous to human IL-8), ultimately leading to improved cardiac necrosis in myocarditis. Furthermore, blocking NETs through genetic knockout of peptidylarginine deiminase 4 has also been found to alleviate myocardial inflammation and necrosis in CVB3-infected mice.^171,304^

Macrophages were identified as the predominant infiltrates in FM, which serve as scavengers, microbicidal effectors, and regulatory cells in cardiac inflammation.^151,305,306^ Inhibition of cardiac macrophage accumulation or their recruitment of other inflammatory cells has proven beneficial in the management of acute myocarditis. CCR2-deficient mice, which lack the ability to recruit macrophages, exhibit reduced production of pro-inflammatory IL-1 and IL-4, and increased levels of protective IFN-γ and IL-10. The cardiac function of the mice showed improvement following resolution of inflammation. IL-8, a chemokine secreted by macrophages, along with its murine counterpart MIP. Elevated levels of MIP-2 have been observed in m-2, exhibits potent chemoattractant properties for neutrophils and lymphocytes. Elevated levels of MIP-2have been observed in mice with myocarditis, and deletion of the MIP-2 receptor has been linked to a reduction in the severity of myocarditis.^307,308^

CD4^+^ T cells undergo activation and differentiation into four distinct subsets, namely Th1, Th2, Th17, and Tregs, in the context of viral myocarditis. While Th1 and Th17 cells release cytokines such as IL-17, IL-21, TNF, and IFN-γ, thereby exacerbating the progression of viral myocarditis, Th2 and Treg cells exhibit a protective effect against the disease. Numerous studies have shown a correlation between the proportion of CD4^+^ Th cells and the development of viral myocarditis.^309,310^ Additionally, CD8^+^ CTLs play a crucial role in combating viral myocarditis by selectively targeting and eliminating virus-infected cells.

Eosinophilic myocarditis (EM) represents an important form of myocarditis, by eosinophilic infiltration and frequently accompanied by eosinophilia.^268^ However, in some patients, peripheral eosinophilia is absent.^311^ Despite this, some patients may not exhibit peripheral eosinophilia. EMB is necessary for definitive diagnosis. EM typically presents as fulminant myocarditis with a high mortality rate. The etiology of EM remains incompletely understood, with reported associations with infections, hypersensitivity reactions, immune disorders, and malignancies.^312^ The final effectors of myocardial damage primarily consist of eosinophils and their toxic granules, while the pathogenesis is regulated by T cells. Double knockout of IL17A and IFN-γ results in a Th2-biased immune state in mice, rendering them susceptible to lethal eosinophilic myocarditis.^313^ Immune checkpoint inhibitors (ICIs) associated with FM have emerged as a significant ICI-associated toxicity.^285^ ICIs are monoclonal antibodies that target regulatory pathways on T-cells, such as PD-1, enhancing the cytotoxic capacity of T-cells against malignant cells and potentially saving lives. However, immune dysregulation induced by these agents can result in inflammation and dysfunction of multiple organs. FM is a serious adverse effect of ICI therapy. The pathogenesis of ICI-associated myocarditis is not as well elucidated as that of viral myocarditis. Current research suggests that T cell-mediated immunity plays a crucial role in the development of this condition.^314–318^ EMB analysis has shown lymphocytic infiltration in the hearts of patients with ICI myocarditis, supporting the involvement of T cell immunity. Both human and animal studies have observed a pronounced lymphocytic infiltration of CD8^+^ T cells compared to CD4^+^ T cells in the hearts of patients with ICI-associated myocarditis.^314–316,319^ A recent study has demonstrated a significant increase of CD8^+^ cytotoxic effector cells in the peripheral blood of patients with ICI myocarditis, mirroring the rise of effector cytotoxic CD8^+^ T cells in the blood and hearts of PD-1 deficient mice with myocarditis.^314^ These proliferative effector CD8^+^ T cells exhibit distinct transcriptional characteristics, including the upregulation of myocardial-tropic chemokines CCL5, CCL4, and CCL4L2. A previous investigation demonstrated an increase in the expression of CCL3, CCL4, and CCL5, as well as their corresponding chemokine receptors, in the cardiac tissue of mice with autoimmune myocarditis, suggesting a potential pathway for T cell infiltration into the myocardium.^317^

Vasospastic angina (VSA) is now being acknowledged as a distinct manifestation of myocardial inflammation, presenting as episodes of angina at rest that are alleviated by short-acting nitrates and caused by coronary artery vasospasm. The spectrum of symptoms associated with VSA ranges from asymptomatic cases and angina episodes to severe cardiovascular events, including myocardial infarction, arrhythmias, and cardiac arrest.^320,321^ An autopsy study has revealed the presence of inflammatory cell infiltration, particularly mast cells and eosinophils, in the myocardium of VSA patients.^322^ Recent research has shown a marked increase in inflammatory cytokines and chemokines in both the plasma and myocardium of VSA patients, indicative of a state of myocarditis.^323^ The levels of IL-6, IL-12p70, IL-15, IL-13, IL-10, PD-L1, MIP-1α, and MIP-1β were found to be elevated in patients with VSA compared to both normal participants and patients with acute myocardial infarction. Our observations revealed the occurrence of coronary spasm induced by mild myocarditis and FM, as indicated by EMB demonstrating inflammatory cell infiltration and coronary angiography showing the disappearance of coronary stenosis upon administration of nitrate esters. Furthermore, patients with myocarditis-induced VSA showed positive responses to glucocorticoid therapy, suggesting a significant role of corticosteroids and inflammation dysregulation in VSA.^323^

### Rescuing and treatment strategies in FM

**Fig. 4:**
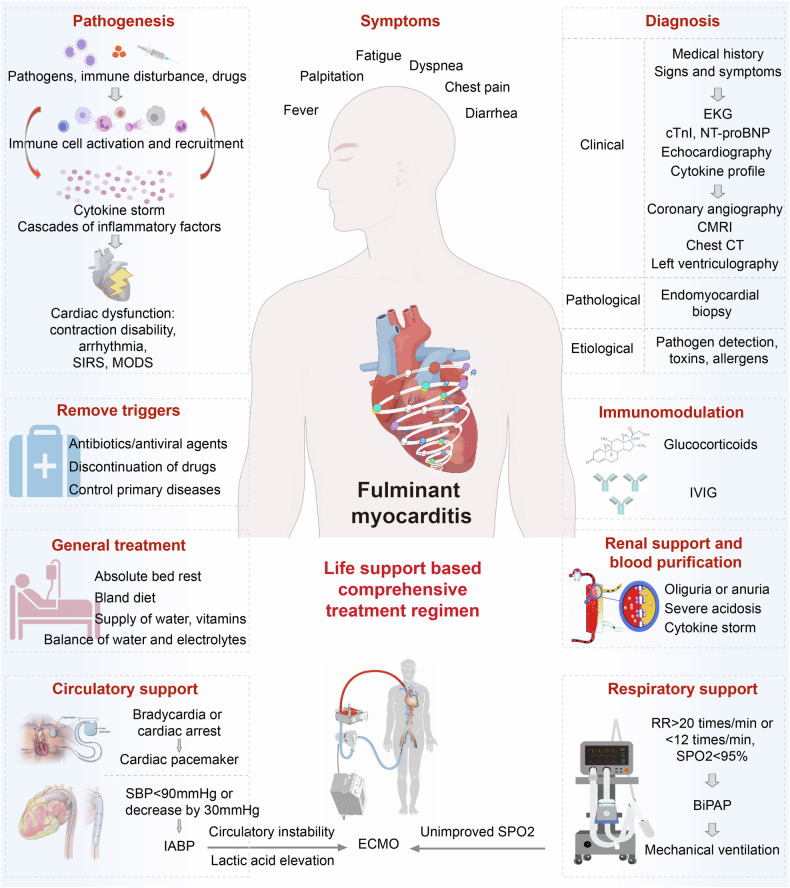
Diagnosis and treatment of fulminant myocarditis. Pathogenesis: pathogens, immune checkpoint inhibitor drugs and allergens activate and recruit immune cells to the myocardium and induce cytokine storm. The cytokine storm threatens cardiac function, causes cardiac contraction disability and arrhythmia, and even multiple organ failure. The symptoms of patients are nonspecific. Diagnosis: the diagnosis of FM includes clinical, pathological and etiological diagnosis. When a patient presents with typical medical history and symptoms, with dramatic progressive circulatory instability or fatal arrhythmia, FM diagnostic procedure should be started. Life support based comprehensive treatment regimen for FM: if a patient is diagnosed with FM, immediate and comprehensive medical care should be initiated. An important aspect of this regimen is the idea of life support. When the patient is suffering circulatory instability, respiratory failure, severe acidosis, mechanical life support such as IABP, ECMO, cardiac pacemaker, ventilation and CRRT should be applied timely. Abbreviations: SBP systolic blood pressure, IABP intra-aortic balloon pump, ECMO extracorporeal membrane oxygenation, SPO2 oxygen saturation, IVIG intravenous immunoglobulin, RR respiratory rate, BiPAP biphasic positive airway pressure. The figure was created with the assistance of Adobe Illustrator

FM is life threatening. However, patients who successfully navigate this critical period and experience complete recovery of cardiac function within 1 month typically have a favorable long-term prognosis.^273^ Therefore, managing the acute phase that is overwhelmed by CS poses the greatest challenge in the treatment of FM. Accordingly, we have conducted a multicenter study and established a regimen termed “life support based comprehensive treatment regimen”, which is an integrated therapy of (i) mechanical life support, (ii) immunomodulation treatment, and (iii) antiviral therapy.^270,324^ The fundamental principle of the treatment regimen involves immune modulation, control of CS, and provision of life support for compromised hemodynamics through the use of MCS devices such as intra-aortic balloon pump (IABP) and extracorporeal membrane oxygenation (ECMO), as well as other life support equipment including ventilators and hemodialyzers (Fig. 4). Mechanical life support aids in circulatory and respiratory function, thereby reducing cardiac workload. The immediate implementation of IABP offers effective circulatory support for patients with FM, resulting in a decrease in in-hospital mortality rates.^324^ Typically, IABP administration elevates systolic blood pressure by more than 20 mmHg and concurrently reduces heart rate by 20–30 beats per minute. If the use of IABP proves ineffective in maintaining circulatory stability, the recommendation is to utilize ECMO. ECMO serves as an alternative method for supporting systemic blood perfusion, while mitigating the potential cardiotoxic effects associated with inotropes and vasopressors. The utilization of ECMO has been instrumental in saving numerous lives during the COVID-19 pandemic.^325^ Nevertheless, there were reports that ECMO application might also induce CS.^326^ Whether this inflammatory response is deleterious or potentially beneficial remains unclear.^326^ Therefore, it is important to keep in mind this possible complication and provide necessary treatments.

In addition to its mechanical support capabilities, MCS aids in reducing myocardial inflammation, restoring normal metabolic function, and modulating cardiac remodeling, all of which are crucial for the restoration of cardiac structure and function.^327^ Mechanical respiratory support also plays a significant role in the treatment of FM. In addition to correcting hypoxemia, the treatment also addresses adult ARDS and reduces cardiac workload. Additional support, such as temporary cardiac pacemaker and continuous renal replacement therapy, may be required as needed. Mechanical life support offers a period of recovery for patients with FM by alleviating organ workload, highlighting the importance of managing cardiogenic shock promptly to prevent further tissue damage, organ failure, or mortality.

Immediate and sufficient application of glucocorticoids (generally 200–400 mg methylprednisolone per day for a few days) and IVIG (20 g per day for 3–5 days) is recommended for immunomodulation in FM. Glucocorticoids serve as potent modulators of inflammation by inhibiting the production of inflammatory mediators during the acute phase of the inflammatory response and dampening downstream signaling pathways.^328^ Specifically, glucocorticoids impede transcription factors downstream of TLR signaling and induce the expression of genes encoding inhibitors of TLR signaling.^329^ Glucocorticoids have been found to alter the mitochondrial metabolism of macrophages, leading to an increase in the production of the metabolite itaconate. Itaconate has been shown to possess anti-inflammatory, antiviral, and antibiotic properties.^328,330,331^ Despite initial concerns regarding the potential for glucocorticoids to promote viral replication, studies have demonstrated that glucocorticoid administration can reduce mortality in viral FM mice and decrease tissue virus titers.^324,332^ Human data demonstrate the potential therapeutic benefits of glucocorticoids in patients with lymphocytic myocarditis confirmed by EMB, regardless of viral status.^333^ Additionally, our recent research has shown that glucocorticoids exhibit antiviral effects by enhancing the production of IFN-γ through the increase of EETs.^334,335^ Clinical studies further support the efficacy of glucocorticoids in managing CS and promoting myocardial function recovery.^288,324^

In the pediatric population, IVIG was initially employed for the treatment of acute myocarditis.^336^ Its therapeutic mechanism involves neutralizing pro-inflammatory cytokines, regulating immune response, and promoting M2 polarization of macrophages through the IVIG Fc fragment. Additionally, IVIG suppresses DC antigen priming and improves outcomes in FM rats.^286^ High-dose IVIG has also been shown to enhance left ventricular ejection fraction in FM patients.^337^ A multicenter study demonstrated that administering high-dose IVIG (1–2 g/kg for several days) to patients with acute myocarditis was associated with improved clinical outcomes, including reductions in inflammatory cytokines, improvements in cardiac systolic function, and decreased mortality rates.^338^

Contrary to this, it is not advisable to use pure immunosuppressive or cytotoxic agents that target lymphocytes, such as azathioprine and cyclosporine, for the treatment of FM. Our research in a mouse model of FM demonstrated that the administration of cyclosporine did not improve the survival rate of mice with myocarditis. Additionally, the Myocarditis Treatment Trial confirmed that cytotoxic drugs did not improve survival rates in FM patients.^339^

Another concept of the “life support-based comprehensive treatment regimen” is antiviral therapy. In our research, oseltamivir was employed as the antiviral agent and significantly enhanced the therapeutic outcomes for patients with FM, regardless of viral status.^324^ The efficacy of oseltamivir in treating FM is attributed in part to its antiviral activity against influenza A and B viruses, as well as its function as a neuraminidase inhibitor. Myocarditis caused by various causes results in the release of neuraminidase from damaged myocardial tissue, leading to an elevation in plasma levels of N-acetylneuraminic acid and exacerbating cardiac injury.^340^ Oseltamivir serves to protect both the heart and the body as a whole from enzymatic damage caused by neuraminidase. This phenomenon elucidates the therapeutic efficacy of oseltamivir in patients with viral-negative FM. Given that viral infection is considered a primary trigger for FM among numerous potential causes, targeted antiviral medications may be utilized upon pathogen detection.

### Viral pneumonia related ARDS

#### Pathogenesis of CS in viral pneumonia related ARDS

**Fig. 5:**
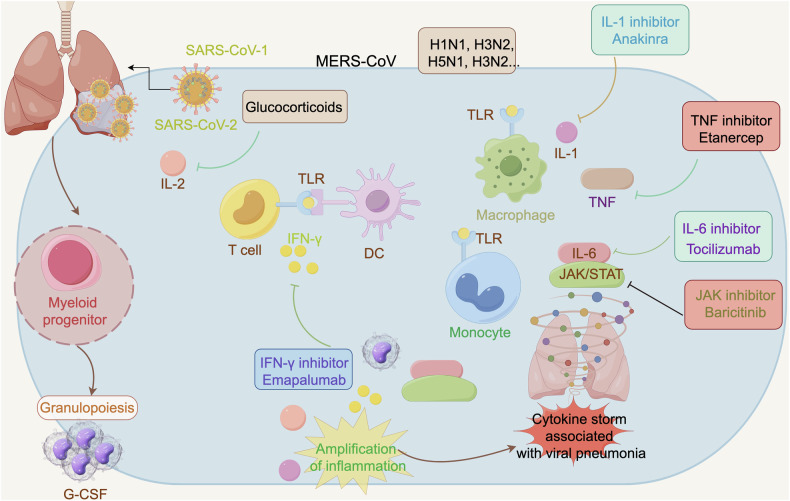
Viral pneumonia-related cytokine storm. Infection and invasion of viruses trigger local immunity, while the activation of inflammation in infected macrophages and immune cells releases proinflammatory cytokines and interleukins, leading to the cytokine release syndrome in severe viral pneumonia. Abbreviation: TLR Toll-like receptor, JAK Janus kinase, STAT signal transducer and activator of transcription, SARS-CoV severe acute respiratory syndrome-coronavirus, IL interleukin, IFN-γ interferon γ, MERS-CoV Middle East Respiratory Syndrome Coronavirus, TNF tumor necrosis factor, G-CSF granulocyte colony-stimulating factor. The figure was created with the assistance of FIGDRAW

ARDS is a severe medical condition characterized by rapid onset of lung inflammation and injury, leading to impaired oxygenation.^341^ ARDS can be classified into extrapulmonary and intrapulmonary subtypes, depending on the underlying cause of injury. Extrapulmonary ARDS is typically associated with systemic conditions such as sepsis or trauma that indirectly affect the lungs, whereas intrapulmonary ARDS is caused by direct lung insults like pneumonia or aspiration.^342^ ARDS is often complicated by the development of CS, with certain viral pneumonias known to trigger these immune responses and worsen the severity of ARDS.^343^ The coronavirus diseases, particularly COVID-19 and SARS, are known for their capacity to elicit an exaggerated immune response.^344,345^ Similarly, influenza viruses such as H1N1 and H5N1 strains, which have caused pandemics, also provoke similar reactions.^346–348^ Therefore, it is crucial to address these responses in order to mitigate the severe outcomes of ARDS in viral-induced pneumonias. Coronaviruses are classified into four categories: α, β, γ, and δ, with the pandemic strains COVID-19, MERS, and SARS belonging to the β type. Coronavirus pneumonia has been shown to result in varying degrees of severe pneumonia and even ARDS.^349–352^ Autopsy reports of deceased COVID-19 patients have confirmed the presence of an overactive immune response and elevated levels of cytokines, suggesting the occurrence of a CS similar to that seen in SARS and MERS.^353–355^ CS is an immune response triggered by the virus (Fig. 5).^356^ The synergistic effects of multiple cytokines result in continuous reinforcement and amplification, ultimately leading to self-directed attacks on the body, resulting in significant tissue and cellular damage that can potentially result in multiple organ dysfunction syndrome.^19^ Consequently, the viral load in the advanced stages of ARDS is just one of several crucial factors influencing the disease progression, with immune activation induced by CS exacerbating systemic organ damage. The timely intervention to inhibit CS is pivotal in preventing the escalation of the disease from a mild or moderate state to a severe one.

The overproduction of cytokines in pneumonia can result in significant pathological alterations, as outlined in Table 3. In pneumonia-associated CS, the JAK signaling pathway plays a crucial role in transmitting intracellular signals downstream. Various cytokine receptors are linked to specific JAKs, suggesting the potential for targeted inhibition of specific JAK functions while preserving the normal functioning of other JAK pathways. The IL-2/IL-2R/JAK (JAK1 and JAK3)/STAT5 signaling pathway is essential for the proliferation and differentiation of NK cells, CD8^+^ T lymphocytes, CD4^+^ T lymphocytes, and other immune cells. IFN-γ exhibits a similar role to that observed in aGVHD and CAR T cell therapy-induced CRS.^357^

**Table 3:** CS in viral pneumonia

Virus type	Viral pneumonia	Main effector cells	Cytokines	Mechanism of CS
SARS-CoV-1	SARS	T cells, respiratory epithelial cells	IL-1β, IL-6, IL-8, IL-12, IL-10, MCP-1, IFN-γ	The deterioration of SARS-CoV infection might result from the combination of direct viral damage and immunopathology caused by CS.^587^
MERS-CoV	MERS	Epithelial cells, DCs, activated T-cells, monocyte/macrophage	IL-15, IL-17, IFN-γ, TNF	MERS-CoV infections of T cells cause programmed cell death, through both internal and external programmed cell death mechanisms, and might promote viral transmission and intense immunopathogenesis.^354,588^
SARS-CoV-2	COVID-19	Respiratory epithelial cells, Tregs, CD4^+^ T cell, CD8^+^ T cell, eosinophils	IL-2, IL-4, IL-6, IL-7, IL-10, G-CSF, IP-10, MCP-1, MIP-1α, IFN-γ, TNF	The IFN-I characteristics of severe COVID-19 patients were significantly impaired. These immune mechanisms may lead to cell death, excessive inflammation, and CS.^589^
H1N1, H3N2, H5N1, H1N1, H3N2 and H7N9	Influenza viral pneumonia	Epithelial cells, endothelial cells, alveolar macrophage, and adaptive immune cells	IFN-α, IFN-β, TNF, IL-1β, IL-18, IL-6, IL-17	The excessive production of pro-inflammatory cytokines leads to positive pro-inflammatory reactions and insufficient control of anti-inflammatory reactions causes CS.^590^

*SARS-CoV* severe acute respiratory syndrome -coronavirus, *IL* interleukin, *MCP-1* human macrophage chemoattractant protein-1, *IFN-γ* Interferon γ, *MERS-CoV* Middle East Respiratory Syndrome Coronavirus, *TNF* tumor necrosis factor, *G-CSF* granulocyte colony-stimulating factor, *IP-10* Human interferon-inducible protein 10, *MIP-1α* macrophage inflammatory protein-1α

CS is a prominent contributor to the mortality associated with severe cases of influenza.^358^ Influenza virus infections are well-known for their propensity to induce lung injury-related fatalities, particularly during pandemics, when mortality rates can significantly rise.^359^ While the prognosis of influenza virus infection is influenced by viral load, the host’s inflammatory response to the virus is closely linked to the development of influenza-induced lung injury.^360,361^ The influenza virus first invades the upper respiratory system by entering epithelial cells through endocytosis. As the infection progresses, it can result in lower respiratory tract infection.^362^ The virus specifically targets epithelial cells, endothelial cells, and alveolar macrophages, inducing an initial release of cytokines essential for virus elimination. This early cytokine response is designed to aid in viral clearance, followed by activation of the adaptive immune system, leading to a secondary cytokine response. The exaggerated immune response, known as CS, plays a crucial role in the increased mortality rates seen in cases of influenza virus infections, particularly during severe outbreaks.^363^ This immune overreaction can lead to significant immunological harm, resulting in severe health complications and worsening the overall disease prognosis.^364^ In severe cases, the CS can induce ARDS.^347^ The characteristic alveolar changes observed in influenza virus pneumonia, resulting from the CS, include capillary thrombosis, localized necrosis, congestion of the alveolar walls, infiltration by inflammatory cells, development of hyaline membranes, and onset of pulmonary edema. These alterations collectively demonstrate the profound immunopathological effects on the lungs.^365^ Severe epidemic pneumonia may result in small vessel thrombosis, bleeding, and diffuse alveolar injury, indicating coagulation dysfunction.^366^ The presence of coagulopathy has been shown to augment the immune response through the induction of CS, as evidenced by the activation of lung endothelial cells, diffuse intravascular coagulation, vascular leakage, and pulmonary microembolism.^367^ Additionally, a severe CS can result in the development of multiple organ dysfunction syndrome, systemic inflammation, and potential mortality.^368^

Cytokines are essential for mediating intercellular communication within the immune system and are crucial for orchestrating an efficient defense against infectious pathogens.^369–371^ Viral RNA has the capability to induce the release of IL-1β and IL-18 by activating inflammasomes through MAVS and NLR.^372,373^ During the adaptive immune response phase, various subsets of T cells and type 2 innate lymphoid cells are activated and modulated. These immune reactions collectively contribute to the elimination of the virus. However, an excessive immune response may result in the overproduction of proinflammatory cytokines, leading to the development of an uncontrolled CS, systemic inflammation, organ dysfunction, and potentially fatal outcomes.^374,375^

Excessive IL-1β has been shown to exacerbate disease and lead to severe outcomes in individuals infected with H1N1, H3N2, and H7N9 viruses. Treatment with targeted anti-IL-1β antibody therapy has demonstrated efficacy in reducing lung inflammation and improving survival rates in both early and late stages of H1N1 or H3N2 infection.^376^ In the context of H7N9 virus infection, NLRP3^-/-^ and caspase-1^-/-^ mice exhibited higher survival rates compared to wild-type mice. This was attributed to lower levels of IL-1β in NLRP3^-/-^ and caspase-1^-/-^ mice, as the absence of caspase-1 during H7N9 infection resulted in reduced recruitment of pro-inflammatory cells to the lungs. Apoptosis related spot like protein knockout (ASC^-/-^) and IL-1R1^-/-^ mice demonstrated reduced lung inflammation and increased survival rates following H7N9 infection.^377^ In the context of the CS induced by influenza virus, IL-1β, and IL-18 play a regulatory role in the production of TNF and IL-6.^378^ H3N2 infection leads to elevated sIL-6R expression, and the expression of IL-6 during influenza virus infection is dependent on sIL-6R.^379^ IL-17 production by influenza virus-activated γδ T cells can exacerbate the inflammatory response during viral infection.^380,381^ Hypercytokinemia induced by Th-17 was identified as an initial host response in severe cases of H1N1 infection in 2009.^382,383^ Following infection with the influenza virus in mice lacking IL-17RA, a reduction in neutrophil cell migration, mild inflammation, preserved lung parenchyma, and decreased morbidity and mortality were observed.^384^

#### Prevention and treatment of CS in viral pneumonia related ARDS

**Fig. 6:**
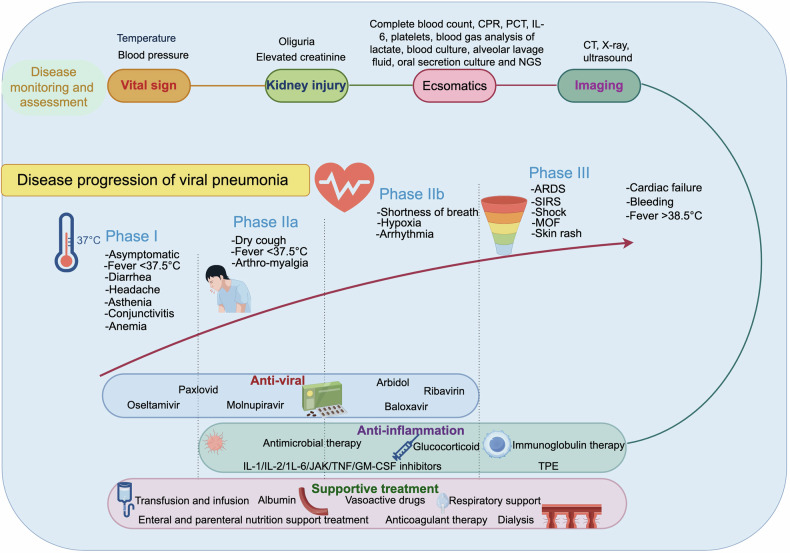
The treatment system of viral pneumonia. Antiviral drugs should be applied as soon as possible, and the best time to use anti-inflammatory drugs is when the inflammatory response is most obvious. There are also some strategies to monitor and assess diseases: vital signs, kidney injury, ecsomatics and imaging. Abbreviation: CPR cardiopulmonary resuscitation, PCT procalcitonin, ARDS acute respiratory distress syndrome, SIRS systemic inflammatory response syndrome, MOF multiple organ failure, TPE therapeutic plasma exchange. The figure was created with the assistance of FIGDRAW

The treatment strategy for viral pneumonia is integrated (Fig. 6). The effects of glucocorticoids have been extensively researched in SARS and MERS. Glucocorticoids are used to suppress CS symptoms and to improve ARDS.^385^ However, a review by Russel et al. found that corticosteroid treatment did not lead to improved 90-day mortality rates in patients. The immune system shows obvious heterogeneity between individuals, and gene expression in immune cells also varies significantly between individuals. These variations lead to great differences in individual susceptibility to immune-related diseases.^386^ It was found that after being infected with SARS-CoV-2, the early response of immune cells related to monocytes and interferon varied among different populations (such as those from Central Africa, Western Europe, and East Asia). Influenced by environmental, genetic, and evolutionary selection pressures, these populations produced different immune responses, which, to a certain extent, can explain the genetic and immunological mechanisms underlying the different susceptibilities of various races to SARS-CoV-2.^387^ Sex susceptibility to CS has also been reported, and this difference mainly stems from the regulatory effect of hormones on the immune system. The testosterone levels and estrogen levels affect the balance between two key immune signaling systems: antiviral IFN-1 and pro-inflammatory signaling such as TNF. Monocytes have an increased testosterone pro-inflammatory response, explaining the more frequent CS in severely infected men.^388^

There are more benefits from the use of glucocorticoids in COVID-19 viral pneumonia patients with chronic obstructive pulmonary disease or asthma.^389^ Dexamethasone has extensive immunosuppressive effects, which can improve the survival rate of COVID-19 patients by inhibiting the in vitro cytokine expression in peripheral blood mononuclear cell induced by SARS-CoV-2.^390^ In another clinical trial that involving hospitalized patients, dexamethasone reduced the 28-day mortality in COVID-19 patients with invasive mechanical ventilation or oxygen alone.^391^ Overall, the dexamethasone-based combination therapy has achieved better therapeutic results in COVID-19^392,393^ IL-6 and CD8^+^ T cell counts serve as dependable prognostic markers for evaluating patient risk and predicting mortality in cases of COVID-19.^394^ Tocilizumab, a recombinant human IL-6 monoclonal antibody, effectively inhibits IL-6 signaling and modulates inflammatory reactions. The administration of tocilizumab, an IL-6 receptor blocker, has demonstrated positive outcomes for critically ill COVID-19 patients necessitating organ support in intensive care units.^395,396^ Treatment with the IL-6 receptor antagonist tocilizumab in critically ill patients with COVID-19 can improve outcomes.^395^ However, research reported that tocilizumab in the absence of mechanical ventilation can reduce the incidence of composite outcomes of progression to mechanical ventilation or death but didn’t improve survival.^397^ Other research found tocilizumab and systemic corticosteroids improved survival and other clinical outcomes of hospitalized COVID-19 patients.^398^ The JAK 2 inhibitor fedratinib was proposed to inhibit the Th17 cytokine.^399^ JAK2 mediates IL-6 and IL-23 signaling in Th17 cells through STAT3. The JAK1 and tyrosine kinase 2 receptors act through STAT1 and STAT2, which are important for the function of activiral immunity. Fedratinib is currently approved by the FDA for myeloproliferative tumors utilizing the JAK2 pathway. During the 2009 H1N1 virus pandemic, a prospective cohort study explored the effect of convalescent plasma therapy in patients with H1N1 pneumonia. The results showed that cellular factors such as viral load, IL-6, IL-10 and TNF significantly decreased in the treatment group, and no adverse events were recorded with plasma therapy,^400^ but in COVID-19 patients, high-titers convalescent plasma did not improve survival.^401^

### SIRS

#### Pathogenesis and mechanism of CS in SIRS

Since 1991, sepsis has been commonly characterized as a systemic inflammatory response to microbial infection, as defined by the presence of at least two manifestations of tachypnea, tachycardia, fever or hypothermia, leukocytosis or leukopenia, and neutropenia.^402^ This definition underscores the role of the body’s exaggerated inflammatory response in the pathogenesis of sepsis. However, it is important to note that the clinical manifestations of SIRS may not accurately capture the complexity of sepsis in critically ill patients. The redefinition of sepsis 3.0 in 2016 emphasizes that sepsis is characterized by life-threatening organ dysfunction resulting from a dysregulated host response to infection.^403^ This updated definition underscores the significance of immune imbalance following infection as the primary driver of sepsis, leading to severe organ damage and potential mortality. While the traditional reliance on clinical manifestations of SIRS for diagnosis has been discarded, the central roles of CS and immune dysregulation in the pathogenesis of sepsis remain paramount.

It is widely accepted that Gram-negative bacteria endotoxin lipopolysaccharide (LPS) triggers mononuclear/macrophage dominated immune cells to release significant quantities of TNF through recognition of PAMPs or DAMPs. The traditional progression of sepsis involves a cascade of inflammatory mediators that lead to an uncontrolled inflammatory response, immune dysfunction, elevated metabolic activity, and damage to multiple organ functions.^404^ This process is initiated by TNF, which activates inflammatory responses through its downstream receptor TNFR1 and collaborates with other inflammatory factors such as IL-6 to rapidly amplify the cascade effects through the automatic amplification of inflammatory mediators, ultimately inducing SIRS.^405^ In the presence of inflammation, IL-6 induces an upregulation of C5a receptor on endothelial cells, thereby augmenting their sensitivity to C5a and subsequently increasing vascular permeability.^406^ Furthermore, IL-6 has been demonstrated to induce myocardial dysfunction. Consequently, the diverse effects of IL-6 contribute to the development of tissue hypoxia, hypotension, myocardial dysfunction, DIC, and multiorgan dysfunction, all of which are hallmark features of SIRS and septic shock.^216^ The CS resulting from the significant release of IL-1 and TNF has the potential to induce myocardial suppression, vasodilation, tissue injury, and mortality through the upregulation of nitric oxide synthesis, myocardial cell contraction, facilitation of immune cell adhesion and activation, and initiation of exogenous coagulation pathways.^407^

#### Prevention, rescue, and treatment strategies of CS in SIRS

It is imperative to prioritize the identification of high-risk populations for SIRS in order to effectively prevent and treat the condition.^408^ Particularly, elderly individuals, those who are malnourished, who have underlying diseases, or who are immunocompromised should undergo prompt assessment to determine the site of infection and potential pathogens. Additionally, for patients with severe infections, timely screening for cytokines is recommended.^409^

Infection control primarily involves identifying pathogenic microorganisms, administering anti-infective therapy, and eliminating sources of infection. Current rapid detection techniques encompass streptococcus pneumoniae urine antigen detection and various platforms for bacterial detection, such as the G test and GM test.^410,411^ Additionally, fungal detection methods, including antigen and nucleic acid testing, as well as Realtime PCR testing, are utilized for fungus detection. The advancement of second-generation sequencing technology has progressed significantly, enabling its widespread application in clinical settings for the detection of numerous pathogens and reducing the likelihood of false negatives. This technology has emerged as a valuable tool for pathogen diagnosis. It is imperative that antimicrobial treatment be promptly initiated, ideally within 1 h of diagnosis, with antibiotics administered within 4 h. Furthermore, it is advisable to collect pathogen specimens prior to the initiation of antibiotic therapy. In cases of SIRS with identifiable etiology, antibiotic therapy alone may be challenging, and achieving efficacious outcomes may be difficult, necessitating the integration of prompt interventions alongside localized treatment of the infectious focus. Mitigating CS represents a critical strategy in the prevention and interception of sepsis. Screening for cytokine levels should be conducted in suspected sepsis patients exhibiting signs of CS to ascertain the extent of SIRS. Contemporary investigations indicate the involvement of numerous cytokines in the CS associated with sepsis. The primary factors implicated in SIRS and CARS encompass various cytokines such as TNF, IL-1, IL-6, IL-12, MIF, sCD74, HMGB-1, as well as anti-inflammatory cytokines including IL-4, IL-10, IL-35, IL-37, TGF-β, and IL-13. Evidence suggests that the management of inflammation should commence when levels of pro-inflammatory factors are significantly elevated or when there is a dysregulation in the inflammatory response. Therefore, for patients at high risk of sepsis infection, regular monitoring of cytokine levels is recommended to promptly identify individuals suspected of developing sepsis. Tregs are essential for maintaining immune tolerance. This process involves the inhibition of T cell activation and proliferation within the body, the production of anti-inflammatory factors like IL-10 and TGF-β, and the promotion of Treg cell proliferation through the activation of the TNFR2 receptor.^412^ These mechanisms collectively contribute to the maintenance of immune homeostasis, the eradication of pathogens, and the prevention of immune overload.^413,414^ Current research on specific antibodies targeting inflammatory mediators, such as TNF, IL-1, and anti-endotoxin LPS antibodies, has shown promise in alleviating sepsis by inhibiting pro-inflammatory cytokines.^415^

In cases where infected patients exhibit a marked elevation in cytokine levels or an imbalance in inflammation, prompt intervention to regulate inflammation is recommended in order to restore a stable and harmonized inflammatory response within the body. The early administration of glucocorticoids has been shown to effectively suppress the secretion and release of inflammatory cytokines in patients with sepsis. Nevertheless, the challenge remains in accurately determining the appropriate timing for the initiation of glucocorticoid therapy in the absence of reliable methods for detecting SIRS. Furthermore, ustestatin, a non-steroidal anti-inflammatory drug, has been shown to impede the release of lysosomal enzymes, suppress the production of myocardial inhibitory factor, eliminate oxygen free radicals, and inhibit cytokine release. During the initial phases of cytokine elevation, the administration of low-dose ustestatin may exert a modulatory influence on cytokines. However, in the event of cytokine dysregulation, the equilibrium between pro-inflammatory cells and anti-inflammatory cytokines is disrupted, resulting in impaired organ function. The administration of high doses of ustestatin has been shown to effectively inhibit the progression of sepsis-related indicators in patients.

### HLH

#### Pathogenesis of HLH

**Fig. 7:**
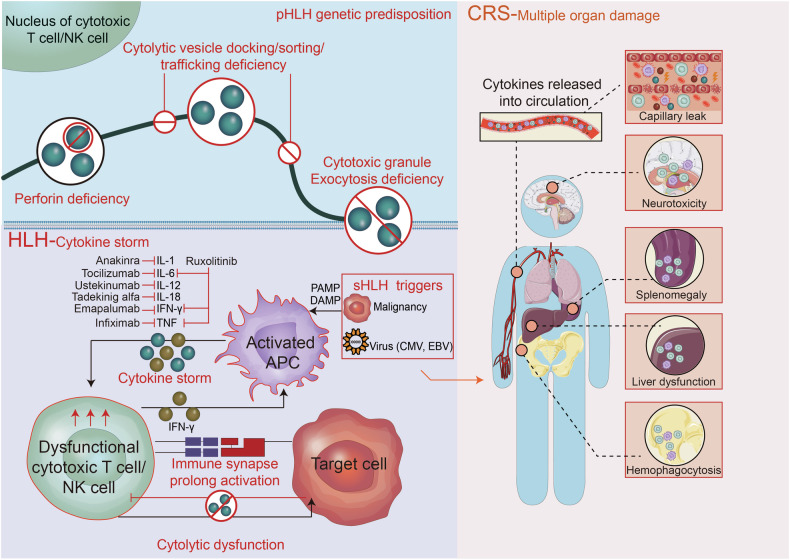
Mechanisms and clinical manifestation of HLH-associated CRS. Upon recognition of a target cell, cytolytic T/NK cells polarize preformed, perforin-containing lytic vesicles toward the immunologic synapse, facilitating perforin release to form pores and deliver cytotoxic proteins into the target cell. In primary HLH (pHLH), genetic mutations affecting perforin-mediated cytolysis impair the lytic pathway, resulting in prolonged interactions between cytolytic T/NK cells and target cells. This extended engagement increases the production of inflammatory cytokines (e.g., IFN-γ), leading to hyperactivation of APCs and subsequent hypercytokinemia. In secondary HLH (sHLH), APCs are activated by PAMPs or DAMPs from malignancies and viruses, causing multi-organ dysfunction, including damage to the vascular endothelium, central nervous system, spleen, liver, and bone marrow. Abbreviation: HLH hemophagocytic lymphohistiocytosis, APCs antigen-presenting cells, PAMPs pathogen-associated molecular patterns, DAMPs damage-associated molecular patterns, IFN interferon, TNF tumor necrosis factor, CMV human cytomegalovirus, EBV Epstein-Barr virus. The figure was created with the assistance of Adobe Illustrator

HLH is a rare and potentially life-threatening hyperinflammatory response syndrome characterized by dysregulated activation of cytotoxic T-lymphocytes, NK-cells, and macrophages resulting in a cascade of hypercytokinemia and immune-mediated harm to various organ systems (Fig. 7).^416–419^ Depending on the underlying cause, HLH is classified into primary and secondary forms. Primary HLH typically presents with a familial history of the disorder and/or genetic abnormalities, and predominantly manifests in childhood.^420^ Primary HLH (pHLH) is associated with abnormalities in genes such as PRF1, Unc13D, Syntaxin-11, STXBP2, and UNC18B, while SH2D1A/SAP and BIRC4 also contribute to the development of pHLH. These genes affect processes such as perforin-dependent granzyme exocytosis, transport, and loading in CTLs and NK cells.^421^ Therefore, the pathogenesis of primary HLH is characterized by hyper-immune activation, resulting from the diminished or absent function of NK cells and cytotoxic lymphocytes. Various immune cells remain persistently activated, continuously secreting cytokines and chemokines such as IFN-γ, TNF, IL-1β, IL-2, IL-6, IL-12, IL-16, and IL-18, leading to a severe CS.^422^ Mouse models with perforin deficiencies have confirmed that elevated IFN-γ secretion by CD8^+^ T cells plays a crucial role in the disease’s pathogenesis.^423^ Syntaxin-11 deficient mouse models indicate that T cell exhaustion is a critical factor in determining the severity of HLH disease.^424^

Recent research advancements indicate that there are about 30 genes linked to HLH, with the primary subtypes being the familial HLH (FHL) family class, the immunodeficiency syndrome class, and the EBV-driven class (Table 4). Furthermore, HLH has transitioned from being solely a recessive genetic disorder to one that can manifest through both recessive and dominant inheritance patterns. Additionally, individuals infected with EBV may exhibit an inherent immunodeficiency that not only predisposes them to HLH but also increases their risk for developing EBV-associated lymphoma.^425^ Age has traditionally served as a distinguishing factor between primary and secondary HLH. Nevertheless, the growing number of hereditary cases identified in adolescents and adults contradicts this assumption. The correlation between genetic mutations and adult HLH patients is currently being investigated both domestically and internationally, with no established theory to elucidate the development of primary HLH in adulthood. Some researchers suggest that the delayed onset of primary HLH in adults may be linked to the specific location of the gene mutation, the manner in which the mutation occurs, and the presence or absence of a triggering factor.^426–429^ For instance, missense mutations and shear site mutations may manifest at an advanced age, while complex heterozygous mutations typically exhibit a later onset compared to pure heterozygous mutations.^428–430^ Additionally, specific sub equivalent loci typically remain quiescent but can be activated by external stimuli, such as an infection, leading to their development.^426^

**Table 4:** Genetic Mutations Associated with HLH

Disorder	Gene	Cytogenetic localization	MOI	Pathogenic pathway
Familial HLH^421^				
FHL-2	PRF1	10q21-22	AR	Pore formation
FHL-3	UNC13D	17q25	AR	Lymphocyte granule priming
FHL-4	STX11	6q24	AR	Lymphocyte granule fusion
FHL-5	STXBP2	19p13.2	AR	Lymphocyte granule fusion
Pigmentary disorders associated with HLH^421^				
GS-2	RAB27A	15q21	AR	Lymphocyte granule docking
CHS	LYST	1q42-43	AR	Lymphocyte granule trafficking
HPS2	AP3B1	5q14.1	AR	Lymphocyte granule trafficking
XLP-1 and XLP-2^421^				
XLP-1	SH2D1A	Xq25	XLR	Defective 2B4-mediated cytotoxicity, impaired T-cell restimulation-induced cell death, absent iNKT cells
XLP-2	XIAP	Xq25	XLR	Dysregulated NLRP3 inflammasome; increased effector cell susceptibility to cell death
Autoinflammation, enterocolitis^421^	NLRC4	2p22.3	AD	Constitutively active NLRC4 inflammasome
NOCARH syndrome^421^	CDC42	1p36.12	AD	Impaired actin structure formation, defective cell proliferation, migration, cytotoxicity, increased IL-1β and IL-18
EBV susceptibility disorders				
X-linked immunodeficiency, magnesium defect^421^	MAGT1	Xq21.1	XLR	Impaired Mg^2+^ transporter; low NKG2D, reduced cytotoxicity
Lymphoproliferative syndrome 1^421^	ITK	5q33.3	AR	Defective tyrosine kinase function, impaired T-cell expansion, decreased iNKT cells
Lymphoproliferative syndrome 2^421^	CD27	12p13.31	AR	Impaired T-cell proliferation/cytotoxicity against EBV^+^ B cells, decreased iNKT cells
Lymphoproliferative syndrome 3^421^	CD70	19p13.3	AR	Defective T-cell expansion/cytotoxicity, decreased NKG2D, 2B4, iNKT cells
Immunodeficiency^421^	CTPS1	1p34.3	AR	Impaired CTP synthesis for nucleic acid metabolism; defective cell proliferation, decreased iNKT cells
Immunodeficiency^421^	RASGRP1	15q14	AR	Impaired T-cell activation, proliferation, migration, cytotoxicity, decreased iNKT cells
Hyperinflammatory Disorder				
NCKAP1L-associated hyperinflammatory disorder^591^	NCKAP1L	12q13.13-q13.2	AR	Impaired actin reorganization, early T-cell activation defects, impaired neutrophil migration
RC3H1-associated hyperinflammatory disorder^592^	RC3H1	1q25.1	AR	Chronic hepatitis, dyslipidemia, dysmorphic features, mild intellectual disability
RHOG-associated hyperinflammatory disorder^593^	RHOG	11p15.4	AR	Radiolucent bone lesions, sclerosis, cupping on distal metaphyses
Metabolic Disorders				
Adenosine deaminase deficiency^594^	ADA	20q13.11	AR	Defective nucleic acid metabolism
Purine nucleoside phosphorylase deficiency^595^	PNP	14q13 .1	AR	Defective nucleic acid metabolism
Cobalamin C disease^596^	MMACHC	1p	AR	Defective vitamin B12 metabolism
Immune System Deficiencies				
IL-2 Ra chain deficiency^421^	1L2RA	10p15-14	AR	Impaired T-cell activation and regulation
Common γ chain deficiency^597^	IL-2RG	Xq13	XLR	Impaired T-cell activation and regulation
X-linked agammaglobulinemia^598^	BTK	Xq21.3-g22	XLR	Impaired B-cell maturation and proliferation
Wiskott-Aldrich syndrome^599^	WASP	Xp11.23-22	XLR	Cytoskeletal defects
DiGeorge syndrome^600^	DCGR	22q11.2	AD	Disrupted TBX1 pathway affects pharyngeal arch/pouch development; thymus, parathyroid, and cardiac defects
Hyper-lgD syndrome^601^	MVK	12q24	AR	Defective cholesterol and lipid synthesis
Lysinuric protein intolerance^421^	SLC7A7	14q11.2	AR	Impaired amino acid transport
Multiple sulfatase deficiency^602^	SUMF1	3p26	AR	Impaired sulfatase transcriptional activation
Holt-Oram syndrome	TBX5	12q24.1	AD	Defective cardiomyocyte differentiation
Heme oxygenase-1 deficiency^603^	HMOX1	22q12.3	AR	Impaired heme oxidation to biliverdin

*AD* Autosomal dominant, *AR* Autosomal recessive, *CHS* Chediak Higashi syndrome, *CTP* Cytidine triphosphate, *GS-2* Griscelli syndrome type 2, *HPS2* Hermansky-Pudlak syndrome type 2, *MOI* Mode of inheritance, *NOCARH* Neonatal onset of pancytopenia, autoinflammation, rash, & episodes of HLH, *XLP-1* X-linked lymphoproliferative syndrome type 1, *XLP-2* X-linked lymphoproliferative syndrome type 2, *XLR* X-linked recessive

Secondary or acquired HLH (sHLH) is characterized by mutations that are triggered by external factors such as infections, malignant tumors, rheumatologic disorders, allo-HSCT, drug hypersensitivity reactions, or other underlying causes. The precise pathogenesis remains unclear, but it may involve persistent TLR activation resulting from infection or autoimmune triggers.^431–433^ EBV is the most commonly associated infectious agent with HLH.^434–437^ Chronic active EBV infection (CAEBV) is particularly prevalent in East Asia. A previous study has demonstrated a significant association between CAEBV and HLA-A26, a genetic marker frequently observed in individuals of East Asian descent.^437,438^ In China, EBV was identified as the primary etiology in 44.01% of 1445 cases across 31 regions, making it the most prevalent cause of HLH.^439^ HLH triggered by EBV infection is more prevalent in children and adolescents with mutations in genes linked to familial HLH and primary immune disorders, such as X-linked lymphoproliferative syndromes types 1 and 2.^440^ In adults, EBV-associated HLH is predominantly induced by immunosuppression leading to reactivation of the virus. CAEBV was previously believed to induce lymphocyte cytotoxicity, such as HLH, through systemic inflammation and clonal proliferation of EBV-infected T or NK cells.^437^ However, a recent study by Wang et al. demonstrated that EBV infects a wide range of cells within the hematopoietic system, including both lymphoid and myeloid lineages, as well as hematopoietic stem cells in patients with CAEBV. This suggests that CAEBV disease may stem from the infection of hematopoietic stem cells.^388,441^ Our prior research demonstrated a correlation between lymphocyte cytotoxicity and genetic mutations in nonimmunosuppressed patients with EBV^+^T/NK-lymphoproliferative disorders. Furthermore, patients with genetic defects exhibited a poorer clinical prognosis when compared to those without mutations.^436^

sHLH has been documented to have a correlation with GVHD following allogeneic or autologous HSCT. Upon diagnosis, mortality rates among these patients are notably elevated, underscoring the importance of promptly identifying and addressing excessive inflammation. There is evidence suggesting that ferritin levels may not be strongly associated with GVHD, but rather with HLH. Elevated ferritin levels in the post-transplant setting may indicate the occurrence of secondary HLH in patients, making it a potentially valuable biomarker for distinguishing sHLH/MAS. It is recommended to first test serum ferritin levels in suspected HLH cases, but in both adults and children, serum ferritin levels <500 μg/L may serve as a negative diagnostic indicator for HLH.^442^ Recent research has identified increased levels of various cytokines and chemokines in secondary sHLH/MAS following allo-HSCT, suggesting that the allogeneic reactive state in GVHD may contribute to the development of sHLH/MAS.^443^ Additionally, patients treated with CD22 CAR-T cells exhibited a higher incidence of CAR-T cell-associated HLH (CAR HLH), which may be considered a form of CRS, aligning with the proposed pathophysiology of HLH as an inflammatory process mediated by T cells and the known association of key cytokines with HLH. Numerous cytokines and chemokines linked to HLH in patients with CAR HLH, including IFN-γ, IL-6, IL-1β, IL-18, IL-18 binding protein (IL18bp), IL-8, MIP-1α, CXCL 9, and CXCL 10, were consistently and significantly elevated in comparison to patients without CAR HLH. Conversely, cytokine elevations in patients with CAR T-associated severe CRS, while reaching similar peak levels, were transient and rapidly decreased, indicating a potential association between more severe CRS and CAR HLH.^444^ Recent findings by researchers have revealed a perforin-deficient homozygous mouse model in which antigen-independent CAR-T cell expansion is linked to HLH-like toxicity. The expansion of perforin-deficient CAR-T cells is accompanied by the simultaneous expansion of wild-type T cells, suggesting that T-cell-driven expansion contributes to a secondary inflammatory response.^427^ For sHLH/MAS identification after HSCT/CAR-T-cell therapy, standard screening protocols, such as ferritin levels, are readily available.^445^

#### Recognition, monitoring, and treatment strategies of HLH

HLH is a potentially life-threatening systemic hyperinflammatory syndrome. Early recognition and prompt management are critical to prevent organ failure and reduce mortality. According to the EULAR/ACR guidelines,^446^ HLH should be suspected when the following unexplained or markedly abnormal clinical and laboratory features are present, particularly when occurring concurrently in an appropriate clinical context. These include persistent fever, elevated ferritin, and other inflammatory or damage markers (e.g., CRP, LDH); inappropriate reductions in hemoglobin, platelet count, or white blood cells (neutrophils and lymphocytes); liver dysfunction (elevated ALT, AST, bilirubin); coagulation abnormalities (e.g., low fibrinogen, elevated PT/INR, D-dimer); splenomegaly; and central nervous system involvement. Diagnostic evaluation should include testing for ferritin, fibrinogen, NK cell activity, IL-2Rα (CD25), and other inflammatory biomarkers. For patients with suspected HLH, the need for genetic testing should be carefully considered based on clinical presentation, age, and laboratory findings, as these factors significantly influence diagnostic and therapeutic decisions. The early identification of high-risk patients is paramount. Various scoring systems and modeling studies utilizing serum markers and clinical characteristics have been developed to predict the severity and prognosis of HLH. Li Xiao et al. identified two key prognostic factors in HLH: total cholesterol levels ≤3.11 mmol/L and BUN levels ≥7.14 mmol/L, both of which were associated with poor outcomes.^447^ In addition, Hua Pan et al. developed a prognostic scoring system to identify pediatric patients at high risk for disease progression, which aids in determining the need for second-line therapies, including allo-HSCT.^448^ Zoref-Lorenz et al. demonstrated that an optimized HLH inflammatory (OHI) index, in conjunction with elevated soluble CD25 (>3900 U/mL) and ferritin (>1000 ng/mL), serves as a potent predictor of poor outcomes in HLH associated with hematologic malignancies.^449^ Furthermore, a recent study suggested that the ferritin/platelet ratio post-induction therapy could reliably reflect treatment response in adult HLH patients.^450^ Cheng et al. established an albumin-bilirubin (ALBI) score and classification system, which uses pretreatment albumin and bilirubin levels. ALBI grade 3 was identified as a significant independent predictor of both 30-day mortality and overall survival, indicating a higher risk of mortality in affected patients.^451^ Zhang et al. proposed an early prognostic model incorporating factors such as deep organ hemorrhage, response to initial induction therapy, and serum calcium levels at 8 weeks post-induction, which helps identify patients at elevated risk of mortality within this timeframe.^452^ Additionally, Tingting Cui et al. developed a column-line diagram model that integrates factors such as age, EBV-DNA levels, BUN, sCD25, and PCT to predict high mortality risk during induction therapy.^453^ Another study utilizing machine learning identified that low total cholesterol, high urea nitrogen and bilirubin, and prolonged thrombin time were strongly associated with early mortality, particularly in pediatric HLH patients.^454^

The management of HLH focuses initially on controlling the excessive inflammatory response to halt disease progression, followed by addressing underlying immune deficiencies and managing the primary disorder to prevent recurrence. The HLH-94 and HLH-2004 protocols are the most widely used for initial treatment. The HLH-2004 protocol incorporates cyclosporine A during the induction phase to enhance immunosuppression, but early clinical studies have shown no significant clinical advantage of cyclosporine inclusion at this stage.^455^ As a result, most centers continue to follow the HLH-94 protocol.^422^ For patients with primary HLH, allo-HSCT remains the only curative option. In cases where primary HLH patients are not eligible for immediate allo-HSCT, maintenance therapy is essential to prevent relapse. The HLH-94 protocol recommends etoposide combined with dexamethasone for maintenance, with adjustments to treatment intensity based on patient tolerance to minimize toxicity while maintaining disease control.^455^ Secondary HLH is more complex, often requiring individualized approaches that deviate from the HLH-94/2004 protocols. For instance, CAR-T cell therapy-associated immune effector cell hemophagocytic syndrome is initially managed with corticosteroids as the first-line immunosuppressive treatment. However, prolonged corticosteroid use can impair CAR-T cell function.^456^ Refractory or relapsed HLH requires timely salvage therapy, which may differ from the initial induction regimen. Relapsed cases can also be managed by repeating the original treatment protocol. However, there is no consensus among medical professionals on an optimal salvage therapy regimen for relapsed or refractory HLH. The DEP regimen (a combination of liposomal doxorubicin, etoposide, and methylprednisolone), with or without asparaginase, has shown significant efficacy in adult patients with refractory HLH.^457–459^ In recent years, several targeted therapies have emerged for HLH treatment. Emapalumab, approved by the FDA in 2018, is currently the only drug indicated for refractory or relapsed primary HLH in children (including neonates) and adults.^460,461^ Other promising agents include the JAK1/2 inhibitor ruxolitinib, the CD52 monoclonal antibody alemtuzumab, IL-6 inhibitors, IL-1Ra anakinra, IL-18 inhibitors and TNF inhibitors.^456^ Additionally, gene therapy approaches targeting defective genes associated with XLP1, FHL2, and FHL3 have shown encouraging results in preclinical mouse models, offering potential avenues for future curative treatments.^462–464^

### CRS associated with CAR-T therapy

#### Pathogenesis of CRS associated with CAR-T therapy

**Fig. 8:**
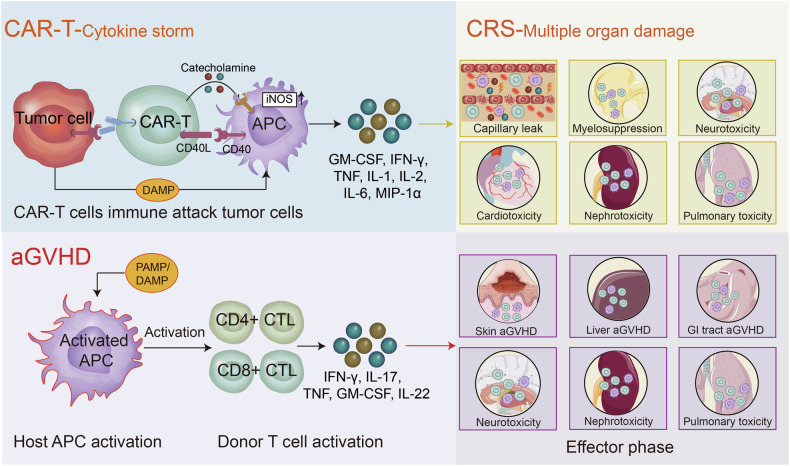
Mechanisms and clinical manifestations of CAR-T and GVHD-associated CRS. CAR-T: Following infusion, CAR-T cells are transported to the tumor site, where target recognition activates them to proliferate locally and produce cytokines such as IL-6, IFN-γ, GM-CSF, and TNF, along with soluble inflammatory mediators and catecholamines. This activation stimulates various components within the tumor microenvironment, leading to increased cytokine levels in peripheral blood and further expansion of the CAR-T cell population. The resulting cytokine storm can trigger systemic inflammation, potentially leading to multi-organ dysfunction. GVHD: Conditioning chemotherapy or radiation causes tissue damage, releasing pathogen-associated molecular patterns (PAMPs; e.g., LPS) and damage-associated molecular patterns (DAMPs; e.g., from total body irradiation, TBI), which increase the activation of host APCs during the initiation phase. In the donor T cell activation phase, these host APCs activate alloreactive donor CD4^+^ and CD8^+^ T cells. In the effector phase, effector T cells and pro-inflammatory cytokines damage epithelial cells of the skin, gastrointestinal (GI) tract, liver, CNS, kidneys, and lungs, leading to apoptosis and necroptosis, and resulting in the symptoms of aGVHD. Abbreviation: CAR-T chimeric antigen receptor-modified T cells, GM-CSF granulocyte-macrophage colony-stimulating factor, IFN interferon, MIP macrophage inflammatory protein, aGVHD acute graft-versus-host disease, APCs antigen presenting cells. The figure was created with the assistance of Adobe Illustrator

CAR-T cell-associated CRS is a systemic condition characterized by the hyperactivation of immune effector cells and a diverse array of proinflammatory cytokines. This syndrome demonstrates a cytokine elevation profile that closely resembles that seen in HLH, with IL-6, IFN-γ, and IL-1 playing key roles (Fig. 8).^1^ CAR-T is a novel therapy for tumor immunotherapy. It has demonstrated the ability to circumvent host immune tolerance and selectively target tumor cells within the major histocompatibility complex (MHC) constraints. Moreover, it exhibits notable advantages including robust targeting capabilities, broad spectrum tumor cell killing, and enduring therapeutic effects. Consequently, CAR-T has been effectively utilized in the clinical management of numerous hematological malignancies.^465,466^ Nevertheless, CRS and ICANS have emerged as significant obstacles impeding the broader application of CAR-T cell therapy in cancer treatment, marked by hyperactivation of the immune system and elevated levels of serum cytokines and pro-inflammatory molecules linked to the proliferation of CAR-T cells (Table 5).^467,468^ Severe CRS and ICANS pose significant risk to patient health. Myeloid-derived macrophages are known to be pivotal in the pathogenesis of CRS.^469^ Research suggests that CRS-related toxicity may primarily involve a macrophage-centric pathophysiological mechanism, characterized by the initial activation of macrophages through CD40L-CD40 interactions within the tumor microenvironment of CAR-T cells, the release of key cytokines (such as IL-6, IL-1, and IFN-γ) during CRS, and the involvement of catecholamine self-amplification loops in macrophages.^470^ Notably, both IL-6 and its downstream effectors are crucial in the manifestation of clinical symptoms associated with CRS. IL-6 is primarily synthesized by activated T cells, with additional contributions from endothelial cells in the vasculature and the monocyte/macrophage lineage in the context of CRS.^471–474^ Elevated IL-6 levels have been associated with vascular permeability, complement activation, DIC, and myocardial dysfunction.^216^ Recent studies have highlighted the role of inducible nitric oxide synthase (iNOS) as a proinflammatory cytokine expressed by M1 macrophages in the pathogenesis of CRS.^470^ IL-1β has been shown to stimulate the expression and synthesis of iNOS. Furthermore, GM-CSF plays a role in a complex network of inflammation, primarily originating from CAR-T cells.^475–478^ Its heightened expression in neuronal cells contributes to the manifestation of neurotoxic symptoms.^479^ IFN stimulation or co-induction with pathogens triggers macrophage activation, resulting in increased ferritin release and subsequent development of severe CRS. Research indicates that interactions between CD40L on CAR-T cells and CD40 on monocyte-macrophage lineages during CRS exacerbate the severity of the syndrome. Macrophages have the capability to respond and secrete catecholamines through the activation of adrenergic receptors,^480^ leading to an increased production of cytokines (IL-2, TNF, IFN-γ, and MIP-1α) within the macrophage, a phenomenon referred to as the autocrine loop of catecholamines.^481^ This cascade of events can exacerbate the inflammatory damage associated with CRS.

**Table 5:** Prediction of CAR-T-associated severe CRS

Predictive models/Indicators	Applicable Diseases	Method of calculation/Risk grouping	Significance
CRS-PSS; ICANS-PSS^604^	LBLC	Bulk >5 cm, Platelets < 150 G/L, No bridge or bridge failure, CRP > 30 mg/L, Female sex, Axi-cel	Score > 2; higher grade ≥ 3 CRS or ICANS
Column line graph model^484^	MM (BCMA CAR-T); ALL, NHL (CD19 CAR-T)	Calculated score based on CX3CL1, GZMB, IL-6, IL-4, PDGFAA. sCRS incidence=exp (score)/(exp (score) + 1)	Score > 0: sCRS% > 50%, identify ≥ grade 4 CRS
Absolute Neutrophil Count (ANC)^605^	MM (BCMA CAR-T); RR B-ALL/NHL (CD19/22 CAR-T)	ANC < 0.01 × 10^9^/L for more than 3 consecutive days	Identify ≥ grade 3 CRS
Three-factor decision tree modeling^485^	B-ALL (CD19 CAR-T)	Three of the 25 clinical factors (including TNF, TG, and PT, etc) were selected to form a decision tree model	Identify ≥ grade 3 CRS
IL-6; Ferritin^606^	MM (BCMA/BCMA CD19 CAR-T)	IL-6 > 14.1 pg/mL, Ferritin > 920 ng/mL	Identify ≥ grade 3 CRS
sST2, AngII, and NETs^486^	Hematologic malignancies (CD19 CAR-T; BCMA CAR-T)	AngII > 1877 pg/mL, sST2 > 38.7 ng/mL, and NETs > 7.5 μg/mL	Prediction ≥grade 2 CRS; Ang-II > 4823 pg/mL or NET > 16.5 μg/mL indicates sepsis
Endothelial Activation and Stress Index (EASIX-pre)^488^	DLBCL, PBMCL, MCL, CLL, FL, ALL (CD19 CAR-T)	Score=LDH(U/L) × Cr (mg/dl)/PLT (10^9^cells/L), High risk: EASIX-pre>4.67	Prediction ≥ grade 3 CRS
EASIX score^489^	LBCL (Axicabtagene ciloleucel)	EASIX-F: score ≥ 4.6, high ferritin (>321 ng/mL); EASIX-FC: score > 2.1, high ferritin(>1583 ng/mL), CRP >21 mgŁ	The higher the risk, the higher the cumulative incidence of grade 2–4 CRS or ICANS
M-EASIX score^490^	B-ALL (1928z CAR-T) and LBCL	Score= LDH[U/L]×CRP[mg/dL]/PLT[10^9^cells/L]	Score ≥ 6.2 predict ≥ grade 3 CRS or ICANS
Decision tree modeling^487^	ALL (CD19 CAR-T)	sVCAM-1 ≤ 2612103.625 pg/ml; sICAM-1 ≤ 145167.594 pg/ml	Prediction ≥ grade 4 CRS
Classification tree model^468^	B-ALL, CLL, NHL (CD19 CAR-T)	High risk: Within 36 h after infusion T ≥ 38.9°C and MCP-1 ≥ 1343.5 pg/mL	Identify ≥ grade 4 CRS
Modeling of cytokine profiles^607^	ALL (CD19 CAR-T)	Three cytokines were selected from 24 cytokines including sgp130, IFN-γ, IL-1RA, MCP-1, etc.	Identify ≥ grade 4 CRS

*MM* multiple myeloma, *BCMA* B-cell maturation antigen, *ALL* acute lymphoblastic leukemia, *NHL* non-Hodgkin lymphoma, *sCRS* severe CRS of grade≥3, *RR* relapsed or refractory, *B-ALL* B-cell acute lymphoblastic leukemia, *TG* triglyceride, *PT* prothrombin time, *IP* inorganic phosphate, *Mg* magnesium, *DLBCL* diffuse large B-cell lymphoma, *sST2* soluble suppression of tumorigenesis-2 factor, *Ang-II* angiopoietin-II, *MCL* mantle cell lymphoma, *CLL* chronic lymphocytic leukemia, *FL* follicular lymphoma, *LBCL* large B-cell lymphoma, *LDH* lactate dehydrogenase, *Cr* creatinine, *PLT* platelet, *CRP* C-reactive protein, *sVCAM-1* soluble vascular cell adhesion molecule, *sICAM-1* soluble intercellular adhesion molecule, *T* temperature, *MCP-1* monocyte chemoattractant protein-1

#### Recognition, monitoring, and treatment strategies of CRS associated with CAR-T therapy

The correlation between the severity of CRS and patient survival is significant. Severe CRS is associated with heightened risks of disability and mortality, impacting the course of patient management. The grading criteria for CRS are typically based on the standards established by the ASTCT (American Society for Transplantation and Cellular Therapy).^482^ This system categorizes CRS severity on a scale from 1 to 4, with grades of 3 or higher indicating severe CRS.^483^ Accurate grading is crucial, as it enables timely identification and management of CRS, potentially improving patient outcomes. However, the ability to predict CRS risk, particularly for early detection of severe cases, remains imperative for optimizing treatment strategies. Numerous studies have investigated methods for identifying and monitoring high-risk CRS patients in order to intervene prior to the onset of severe symptoms, ultimately mitigating CRS severity and mortality rates. These models commonly incorporate pre-infusion laboratory markers and/or post-administration serum levels of cytokines or other immune proteins, including but not limited to absolute neutrophil count, hemoglobin, CRP, ALP, BNP, APTT, PCT, and ferritin. Additionally, cytokine profiles such as IFN-γ, soluble IL-2 receptor, IL-4, IL-6, IL-8, IL-10, IL-15, MCP-1, TNFRp55, CX3CL1, GZMB, PDGFAA, among others, are also employed. Moreover, early changes in ST2, Ang-2, NETs levels, and the soluble forms of vascular cellular adhesion molecule-1 (sVCAM-1) or intercellular adhesion molecule 1 (sICAM-1) within the first 24–48 h post-infusion have been utilized for the early identification of severe CRS patients.^484–487^ Considering that endothelial cell activation plays a crucial role in the pathogenesis of CRS, the endothelial activation and stress index (EASIX) score, incorporating baseline levels of blood creatinine, lactate dehydrogenase, and platelets, as well as the modified EASIX formula, have been employed for the stratification of severe CRS risk.^488–490^ Table 5 presents the current research on predicting CRS. It is worth noting that there is a lack of standardized systems for identifying and monitoring CRS in patients with different hematologic malignancies. Vigilant monitoring and evaluation of disease severity, particularly in high-risk CRS patients following CAR-T cell therapy, are essential, with a focus on various serum biomarkers or cytokines (Table 6).

**Table 6:** Characteristics and management of CAR-T associated CRS

Characteristics Recognition and management	
Signs and symptoms^482^	Initiation: fever with other systemic symptoms (myalgia, fatigue, nausea, vomiting, diarrhea, etc)
	Progress: hypotension, hypoxia, tachycardia, tachypnea, arrhythmia, pleural effusion, capillary leakage, coagulation dysfunction, pulmonary edema, DIC, multiple organ failure
Development time^482^	Within 14 days of CAR T cell infusion
	The median time for common occurrence is 2–7 days
Cytokine profile^482^	IL-6, IFN-γ, TNF, GM-CSF, IL-1, IL-2, IL-10, MIP-1, MCP-1,etc
Risk factor^482^	Patient characteristics: disease type, high disease burden, preexisting thrombocytopenia, and endothelial activation
	CAR-T cell product features: targeted CD19, CD28
	Co-stimulatory domains, receiving fodarabine and cyclophosphamide, high-dose infusion, high levels of serum CAR-T cells
Diagnostic criteria^483^	Rule out infectious causes of fever
	CRS scoring
	Assess ECG, troponin, and BNP levels, atransthoracic echocardiogram for grade 2-4 CRS
Management measure^482^	Antipyretic, intravenous fluid
	ICU treatment, vasopressor support, supplemental oxygen
	Anti-IL-6 therapy, glucocorticoid, anakinra, ruxolitinib, emapalumab, antithymocyte globulin, and/or cyclophosphamide

*DIC* disseminated intravascular coagulation, *GM-CSF* granulocyte-macrophage colony-stimulating factor, *MIP-1* macrophage inflammatory protein, *MCP-1* monocyte chemoattractant protein 1

Various strategies have been suggested for the prevention of CRS, such as optimizing dosage regimens, creating less toxic chimeric antigen receptors (CARs), and implementing reversible switches. However, further research is needed before these strategies can be applied in clinical settings. Currently, the predominant intervention methods involve pharmacological treatments.^491^ CRS prevention can be accomplished by inhibiting the IL-6 receptor using tocilizumab (an IL-6 receptor antagonist) or through monocyte depletion.^492,493^ Although the potential risk of ICANS associated with tocilizumab was initially considered uncertain, studies have now shown that patients who receive multiple doses of tocilizumab are at a heightened risk for both low-grade and high-grade ICANS compared to those who receive fewer doses. Conversely, IL-1Ra (anakinra) has demonstrated efficacy in preventing CRS and neurotoxicity in animal models.^470,494,495^

Early administration of tocilizumab and prompt use of corticosteroids are crucial in managing sCRS. Studies have shown that risk-adapted preemptive tocilizumab effectively prevents grade 4 CRS following CTL019 treatment for pediatric B-cell acute lymphoblastic leukemia, without adversely affecting the antitumor efficacy or safety of CTL019.^496^ The prevailing consensus in current research literature advocates for the implementation of a stepwise treatment approach. In cases where patients present with sCRS symptoms, initial therapy typically consists of tocilizumab and corticosteroids. Specifically, tocilizumab is administered at a dose of 8 mg/kg via intravenous infusion, with a maximum dose of 800 mg, given 1–2 times, not exceeding three doses within a 24-hour period, up to a maximum of four doses.^497^ Additionally, dexamethasone is administered at a dose of 10 mg via intravenous infusion every 6 h, with treatment duration ranging from 1 to 3 days. Alternatively, methylprednisolone may be administered at 1000 mg intravenously daily for 3 days, followed by a taper; however, the optimal dose and timing for corticosteroid administration remain unclear.^482^ If methylprednisolone proves to be ineffective, alternative treatments such as the anti-IL-6 antibody siltuximab and IL-1Ra anakinra have been utilized in certain medical centers for the management of CRS.^482^ Anakinra has been shown to mitigate CRS symptoms in animal models without compromising the efficacy of CAR T cell therapy.^492^ Clinical reports suggest that anakinra is effective in controlling CRS that is resistant to steroid treatment.^498,499^ Additional treatment options for steroid-refractory severe CRS include JAK pathway inhibitors, GM-CSF inhibitors, TNF blockers, tyrosine kinase inhibitors, mTOR inhibitors, blood filtration, plasma exchange, mechanical ventilation, and surgical intervention.^482^ In cases of multi-organ dysfunction, continuous veno-venous hemofiltration can effectively eliminate excess fluids and inflammatory mediators, stabilize the internal milieu, and facilitate the restoration of compromised organs. Additional large-scale clinical trials may be necessary to confirm the effectiveness of these treatments. Despite advancements in current methods for treating and preventing CRS, further research and clinical trials are essential to enhance these strategies and guarantee their safety and efficacy.

### aGVHD post allo-HSCT

#### Pathogenesis of HSCT associated CRS

aGVHD is a significant complication following allo-HSCT, marked by heightened inflammatory reactions due to antigenic disparities between transplant recipients and donors. It stands as the primary contributor to non-recurrent mortality post-transplantation, linked to elevated rates of morbidity and mortality (Fig. 8). This condition affects multiple organs, including the skin, liver, gastrointestinal tract, lungs, kidneys, thymus, lymph nodes, bone marrow, and central nervous system, and is categorized into grades I-IV according to its severity, typically manifesting weeks to months post-HSCT.^500^ The clinical symptoms of this condition are a result of a complex series of events that commence with the activation of host antigen-presenting cells in response to tissue damage caused by pretreatment. Subsequently, donor T cells are stimulated by the activated host antigen-presenting cells to identify host antigens, migrate to affected tissues, and trigger apoptosis.^501^ During the initial phase, PAMPs and DAMPs generated by pretreatment chemotherapy or radiation therapy regimens are detected by innate immune receptors like TLRs, resulting in the secretion of pro-inflammatory cytokines (e.g., TNF, IL-1β, and IL-6) and subsequent activation of host antigen-presenting cells (Fig. 8).^502,503^ Subsequently, in the second phase, the engagement of donor T cells with activated antigen-presenting cells triggers the activation and proliferation of T cells.^504^ During the third effector phase, activated donor T cells and monocytes migrate to the target organs affected by aGVHD, including the skin, liver, spleen, and intestine. These cells stimulate the recruitment of additional effector cells such as cytotoxic T cells and NK cells, which contribute to tissue injury through direct cytotoxicity or the release of proinflammatory cytokines and chemokines such as TNF, IL-1β, IL-2, IL-12, IL-17, IFN-γ, CCL2, CCL3, CCL4, and CCL5. This exacerbates the severity of aGVHD and can ultimately result in patient mortality.^504–506^ Furthermore, research indicates that T cells in individuals with aGVHD predominantly rely on glycolysis as their primary metabolic pathway, suggesting that dysregulated T-cell glycolysis may represent a novel mechanism contributing to the development of aGVHD.^507^

#### Recognition, monitoring, and treatment strategies for HSCT associated CRS

Patients with severe aGVHD frequently demonstrate suboptimal responses to treatment and elevated rates of mortality following transplantation, underscoring the significance of timely identification and monitoring of risk factors. Scholars from both domestic and international arenas have devised diverse predictive models utilizing serum biomarkers, cytokines, and comorbidities. For example, researchers in the domestic sphere have proposed the utilization of the SA/PA ratio (<0.731) on the 7th day post-hematopoietic stem cell transplantation as a means of predicting severe aGVHD.^508^ In the field of international studies, various scoring systems such as the gut microbiota score, HCT-CI score, and DeltaAlb≥0.9 have been utilized to assess the likelihood of developing grade III-IV aGVHD. Additionally, biomarkers including IL-2Rα, TNFR1, IL-6, and ST-2, as well as REG3a identified through the MAGIC algorithm, have shown promise in predicting severe and fatal GVHD within the first 2 weeks post-transplantation.^509–514^ Furthermore, markers of endothelial injury such as Ang-II have been found to be significantly elevated in cases of aGVHD and are associated with lower survival rates in patients with severe aGVHD.^515,516^ Convolutional neural network models, clinical variables, and cytokine gene polymorphism models have demonstrated efficacy in predicting severe GVHD.^517,518^ Furthermore, the ratio of inflammatory CD4/CD8 double-positive T cells and Tim-3CD8 T cells shows promise for early identification of high-risk patients.^519^ Specific prediction studies for aGVHD are outlined in Table 7, highlighting the diversity of predictive methods available. However, it is important to note that these studies may not be universally applicable as they target distinct patient populations. Hence, it is imperative for clinicians to conduct personalized evaluations for distinct demographic groups and implement proactive preventative interventions or immediate interventions for patients at elevated risk (Table 8).

**Table 7:** Prediction of severe aGVHD

Predictive models/Indicators	Applicable Diseases	Method of calculation/Risk grouping	Significance
CNN predictive model^517^	Indefinite	High risk: 90–100th percentile; Intermediate risk: 10–90th percentile; Low risk: 0-10th percentile	Higher scores: predicts grade 3-4 aGVHD
CD4/CD8 double-positive T-cell (DPT)^519^	Indefinite	DPT is not present in the initiating grafts	Presence of DPT predicts ≥grade 2 aGVHD
daGOAT predictive model^608^	All patients	Clinical data, dynamic variables, and benchmarks like MAGIC and Ann Arbor scores	Dynamic forecasting of severe acute graft-versus-host disease
The Minnesota GVHD Risk Score^609^	All patients	Calculation of risk groups based on severity of organ involvement using	Predicted grade 3-4 aGVHD
MAGIC algorithm probability (MAP)^514^	All patients	log10[-log10(1-P^)] = −11.263 + 1.844(log10ST2) + 0.577(log10REG3α). High risk: P^ ≥ 0.16	Predicted grade 3-4 aGVHD
GMS score^509^	ALL; AML; MDS	Calculation of GMS scores based on different gut flora formulas	Higher scores: predicts grade 3-4 aGVHD
CG-M model^518^	AML; ALL; MDS; Lymphoma; Myelofibrosis; MM	Risk scores were calculated based on LASSO multivariate analysis	0.11≤score＜0.3: predicts grade 3-4 aGVHD
SA/PA ratio^508^	ALL; AML; NHL	SA/PA ratio<0.731	Predicted grade 2-4 aGVHD
Gradient of fecal microorganisms^610^	All patients	Gradient=sum of relative abundance of positively correlated bacteria-sum of relative abundance of negatively correlated bacteria	Higher gradient: predicts grade 3-4 aGVHD
TIM3; IL-6; sTNFR1^512^	All patients	TIM3, IL6, sTNFR1	Predicted grade 3-4 aGVHD
Ann Arbor score^611^	All patients	TNFR1, ST2, and Reg3α；High risk (3); Intermediate risk (2); Low risk (0)	Prediction aGVHD
HCT-CI scores^510^	All patients	Weighted according to the corresponding comorbidity score: High risk (≥5); Intermediate risk (1–4); Low risk (0)	Medium-High risk: predicts grade 3-4 aGVHD

*CNN* convolutional neural network, *KRT20* Cytokeratin 20, *daGOAT* Dynamic forecasting of severe acute graft-versus-host disease after transplantation, *AML* acute myeloid leukemia, *MDS* myelodysplastic syndrome, *MAGIC* The Mount Sinai Acute GVHD International Consortium, *p^* probability, *REG3α* regenerating islet-derived 3-α, *GMS* gut microbiota score, *CG-M* clinical variables and cytokine gene polymorphisms, *LASSO* least absolute shrinkage and selection operator, *SA/PA* stearic acid/palmitic acid, *DeltaAlb* magnitude of decline in serum albumin, *HCT-CI* Hematopoietic cell transplantation co-morbidity index

**Table 8:** Characteristics and management of CAR-T associated ICANS

Characteristics Recognition and management	
Signs and symptoms^483^	Initiation: drowsiness, disorientation, inattention, tremors, expressive aphasia, dysgraphia, and apraxia
	Progress: cognitive impairment, focal motor and sensory deficits, epilepsy, fatal cerebral edema, and intracranial hemorrhage
	Long-term sequelae
Development time^482^	Usually occurs concurrently with or shortly after CRS
	Delayed instances starting >3 weeks after CAR T cell infusion
Cytokine profile^251^	Plasma: IFN-γ, IL-15, IL-6, IL-10, GM-CSF, IL-1RA, IL-2, IP-10, IL-1β, IL-8, TNF Cerebrospinal fluid: the cytokine profile was similar to that of serum, except for elevated levels of IL-8, CXCL-10 and MCP-1
Risk factor^482^	Patient characteristics: CRS, disease type, high disease burden, past thrombocytopenia and endothelial activation, and past neurological comorbidities
	CAR-T cell product features: targeted CD19, CD28 co-stimulatory domains, receiving fodarabine and cyclophosphamide, high-dose infusion, high levels of serum CAR-T cells
Diagnostic evaluation^482^	ICE score and/or CAPD score
	Neurology consultation; Brain PET–CT, brain MRI, and EEG
	Rule out infection and leptomeningeal malignancy
	ICANS scoring
Management measure^482^	Supportive treatment
	Glucocorticoids, tocilizumab (only when accompanied by CRS), antiepileptic drugs
	Intrathecal hydrocortisone ± intrathecal chemotherapy, anakinra, siltuximab, ruxolitinib, cyclophosphamide and/or antithymocyte globulin
	ICU treatment, airway protection, special nerve intensive therapy

*GM-CSF* granulocyte-macrophage colony-stimulating factor, *MCP-1* monocyte chemoattractant protein 1, *IP-10* interferon- induced protein 10

The prevailing conventional prophylactic regimen for GVHD commonly involves calcineurin inhibitors (e.g., cyclosporine or tacrolimus) and antimetabolite medications (e.g., methotrexate or mycophenolate mofetil), occasionally supplemented with anti-thymocyte globulin (ATG).^520^ Strategies such as the Beijing protocol, which incorporates ATG and granulocyte colony-stimulating factor, as well as post-transplant cyclophosphamide-based T-cell depletion, have demonstrated efficacy in reducing the occurrence of aGVHD.^521–524^ In the context of reduced-intensity conditioning and matched unrelated donor allo-HSCT, the addition of sirolimus to the standard treatment regimen has been associated with decreased incidence of GVHD and non-relapse mortality, ultimately leading to improved overall survival rates. Abatacept, a T-cell co-stimulation inhibitor, has received approval in the United States for the prevention of GVHD and is well-known for its effectiveness when used in conjunction with calcineurin inhibitors and methotrexate, particularly for HLA-matched or mismatched unrelated donor transplants.^511^ Vedolizumab, which targets the α4β7 integrin, has the potential to prevent acute gastrointestinal GVHD by disrupting T-cell migration to gut-associated lymphoid tissue.^525^

The initial treatment approach for aGVHD typically involves the use of corticosteroids. In cases where patients do not respond well to corticosteroid therapy, known as steroid-refractory aGVHD (SR-aGVHD), ruxolitinib has been approved by the FDA as a viable treatment option. Additional therapeutic strategies for aGVHD include alemtuzumab, α1-antitrypsin, basiliximab, cellular therapies (such as mesenchymal stem cells and regulatory T cells), daclizumab, extracorporeal photopheresis, fecal microbiota transplantation, other JAK inhibitors, mycophenolate mofetil, methotrexate, pentostatin, rabbit anti-thymocyte globulin, sirolimus, and vedolizumab.^520^ In cases of low-risk aGVHD, investigations into monotherapy with alternative agents like itacitinib are currently underway. In the context of high-risk patients, the combination of novel agents with corticosteroids is being explored due to their anti-inflammatory and tissue-protective properties. Recent research has identified potential therapeutic targets for aGVHD, such as the simultaneous inhibition of the pro-inflammatory cytokines IL-6R and TNF in MHC-mismatched mouse models. This approach has shown promise in rescuing recipients from corticosteroid resistance and lethal intestinal GVHD while maintaining graft-versus-tumor effects.^526^ Additionally, the expression levels of ubiquitin-specific protease 11 (USP11) have been found to correlate with the development of aGVHD in patients undergoing allo-HSCT, suggesting that USP11 inhibition may be a viable strategy for both the prevention and treatment of aGVHD.^527^ Furthermore, the synergistic enhancement of T-cell function in aGVHD patients has been demonstrated by combining corticosteroids with glycolysis inhibitors, resulting in a reduction in disease severity in mouse models while maintaining graft-versus-tumor effects. This approach presents a promising avenue for precision therapy in aGVHD.^507^

## Therapeutic prospect in halting CS

Given the significant impact of CS on disease pathology, there has been ongoing research into strategies aimed at quick diagnosis and therapy targeting of CS. Quick diagnostic tools are under exploration. For instance, sST2 detecting kit is developed for quick FM diagnosis based on the finding that serum sST2 levels correlate with FM progression.^34^ Furthermore, a variety of cytokine antibodies or analogs have been developed to either neutralize proinflammatory cytokines or inhibit downstream cascades.

For instance, IL-1, which includes IL-1α and IL-1β, is a key proinflammatory cytokine that promotes destructive inflammation. Anakinra, the recombinant IL-1Ra, has been created to specifically block the activity of both IL-1α and IL-1β. The administration of anakinra resulted in notable and prompt alleviation of fever, as well as a decrease in inflammatory cytokines and biomarkers linked to ICANS/CRS.^528^ This treatment has also demonstrated efficacy in patients with CS related to rheumatic conditions.^529,530^ Similarly, the IL-1β neutralizing antibody canakinumab exhibited anti-inflammatory properties. In a study involving CVB3-induced acute myocarditis in mice, significant enhancements in myocardial injury and inflammation were noted in groups treated with IL-1β neutralizing agents.^531^

Similarly to IL-1Ra, tocilizumab, an IL-6R antibody, has demonstrated efficacy in the treatment of systemic juvenile idiopathic arthritis (sJIA), severe rheumatoid arthritis, multicentric Castleman’s disease, and CART therapy-induced CRS.^58,532–535^ In addition, IFN-γ is a potential target for CS control. Recently, FDA approved emapalumab, an IFN-γ antibody, for the treatment of relapsed/refractory HLH based on positive results from a single-arm, open-label phase 2/3 trial (NCT01818492; NCT02069899).^461^ Furthermore, IFN-γ neutralizing antibodies have been shown to improve survival in mice with LPS-induced sepsis.^62^ Other promising targets for treatment include IL-18 and TNF antibodies or analogs.

In addition to anti-cytokine therapies, the utilization of small molecules to inhibit cytokine production and signaling represents a promising approach. For instance, JAK inhibitors such as baricitinib, tofacitinib, upadacitinib, and ruxolitinib have demonstrated efficacy in clinical settings for managing CS in conditions such as aGVHD, rheumatoid arthritis, sJIA, and systemic lupus erythematosus.^65,536^ Ruxolitinib has shown potential in ameliorating disease symptoms in murine models of primary and secondary HLH. Additionally, baricitinib has been shown to enhance recovery from COVID-19 infection.^537^ Despite these antibodies being approved for certain diseases, there is ongoing research into the efficacy of this treatment for a broader range of conditions.^11^

**Fig. 9:**
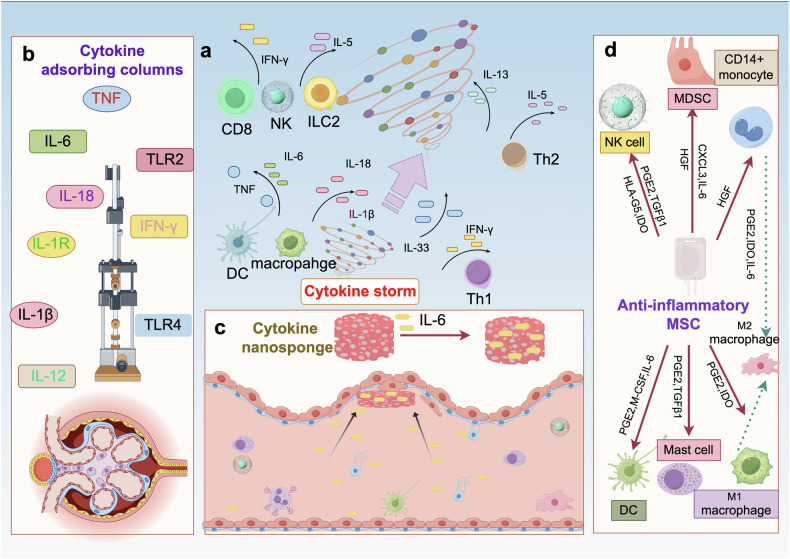
Future therapeutic targets for drugs and new therapeutic approaches in cytokine storm related diseases. **a** The important target factors for anti-inflammation therapy. Blood purification using cytokine adsorption columns (**b**), cytokine nanosponges (**c**), mesenchymal stem cell (MSC) therapy (**d**) are the most promising treatments against cytokine storm. Abbreviation: TLR Toll-like receptor, IL interleukin, IFN interferon, TNF tumor necrosis factor, NK natural killer cells, DC dendritic cells, ILC2 the group 2 innate lymphoid cell, Th helper T cells, PGE2 prostaglandin E2, TGF transforming growth factor, MDSC: myeloid-derived suppressor cells, HLA-G5 human leukocyte antigen G5, IDD intervertebral disc degeneration, HGF hepatocyte growth factor, CXCL3 chemokine (C-X-C motif) ligand 3. The figure was created with the assistance of FIGDRAW

In addition to drug research, new strategies for controlling CS are being developed and have shown promising results. These include cytokine nanosponges, mesenchymal stem cell treatment, and blood purification using cytokine adsorbing columns, all of which offer potential for improved cytokine control (Fig. 9).

### Cytokine nanosponges

Biological neutralization is a promising strategy for mitigating the effects of destructive CS, in which therapeutic agents are employed to bind with inflammatory mediators or infectious pathogens and inhibit their bioactivity. Cellular nanosponges, consisting of cell membrane-coated nanoparticles, are engineered as decoys for biological neutralization purposes.^538^ Cytokine nanosponges are utilized to bind to and eliminate cytokines by presenting identical antigen epitopes as target cells, mimicking source cells, and neutralizing cytokines to disrupt the cytokine cascade in inflammatory disorders.

Macrophages possess a high concentration of cytokine-binding receptors, making macrophage membrane-coated nanoparticles a subject of extensive research for anti-inflammatory therapy. It has been demonstrated that LPS-stimulated macrophage membrane-encapsulated nanoparticles (LMNP) have the ability to attenuate CS in HLH.^539^ By effectively binding various cytokines, LMNP can mitigate HLH symptoms by inhibiting CS and preventing excessive macrophage activation. LMNP shows promise as a therapeutic option for treating lethal HLH in both mouse models and human patients.^539^ Neutrophil membrane-coated nanoparticles, akin to macrophage nanoparticles, have been engineered for the purpose of neutralizing inflammatory cytokines.^538,540^

### Mesenchymal stem cells

Mesenchymal stem cells (MSCs) are a type of multipotent progenitor cell with hematopoietic capabilities, capable of differentiation into various mesodermal lineages.^541^ MSCs possess significant immunomodulatory properties, exerting control over the inflammatory responses of various immune cells such as DCs, macrophages, and lymphocytes.^542–544^ MSCs have the ability to alter the inflammatory environment of CD4^+^ T cells, shifting from an effector T cell dominant microenvironment to one rich in regulatory T cells.^543,545^ Additionally, MSCs inhibit the maturation of DCs, promoting a more tolerogenic regulatory phenotype, and induce the polarization of macrophages into an anti-inflammatory M2 phenotype.

Due to their potent immunomodulatory properties, the use of MSC transplantation (MSCT) for the treatment of immune and inflammatory diseases is a subject of active research.^546^ In 2004, the use of MSCT to treat steroid-resistant severe aGVHD following allo-HSCT was documented in a pediatric patient.^547^ The promising results observed in this case study sparked significant interest in the application of MSCT for immune disorders. Subsequently, two large-scale trials were initiated to investigate the efficacy of MSCT in treating aGVHD, leading to further exploration of its potential in managing other inflammatory conditions. While reports have indicated the effectiveness of MSCT in treating severe cases of COVID-19, discrepancies have been noted among various clinical trials.^548–550^ As of the present time, more than 1000 clinical trials investigating MSC treatment have been recorded in the NIH Clinical Trial Database. These trials focus on a range of diseases including pulmonary inflammation, aGVHD, rheumatic disorders, and other inflammatory conditions. Additionally, the development of new products such as embryonic stem cells and induced human pluripotent stem cells has expanded the potential clinical uses of MSC immunomodulation.

### Blood purification with cytokine adsorbing columns

Blood purification therapies have been employed in the treatment of cytokine-associated diseases, with the β2 microglobulin adsorption column being a prominent method investigated for hypercytokinemia resulting from various causes.^551^ Animal studies have demonstrated a significant decrease in IL-6 and TNF levels in mice with sepsis following treatment with the β2 microglobulin adsorption column. Similarly, human studies on sepsis patients have shown a time-dependent reduction in plasma levels of IL-1β, IL-6, IL-8, and TNF in those receiving treatment with the β2 microglobulin adsorption column.

In addition to the utilization of a β2 microglobulin adsorption column, various other columns have been developed to target novel cytokines and chemokines. One such example is the polymyxin B-immobilized fiber column, which has been employed for endotoxin adsorption therapy in septic shock patients. Most recently, Sekiya et al. introduced a novel adsorption column (NOA-001) designed to eliminate cytokines and activated neutrophils in a rabbit model of ALI.^552^ In the pathogenesis of ARDS and ALI, neutrophils play a crucial role in promoting inflammation through various mechanisms, such as the release of toxic granules, formation of NETs, deposition of platelet-neutrophil complexes in tissues, and activation of other immune cells, ultimately contributing to CS. Therefore, the simultaneous targeting of cytokines and activated neutrophils may potentially halt the progression of ARDS and ALI. Given its demonstrated efficacy in improving pulmonary function in animal models, NOA-001 shows promise as a potential treatment for ARDS in humans.

## Conclusion and prospect

The main characteristics of CS include primary diseases and complications arising from circulating cytokines, acute systemic inflammation, and secondary organ dysfunction that surpass the body’s compensatory capacity, ultimately resulting in irreversible damage. Preventing the onset of CS and enhancing survival rates pose ongoing challenges. With persistent and intensive research into the underlying mechanism and targeted therapies of CS, there have been improvements in CS treatment. However, the overall mortality rate of CS is high. For better control of CS and its subsequent organ damage, three principles are to be followed: comprehensive integration of treatment, targeting the key mediator of different conditions, and choosing the better selective therapy with fewer off-targets.

Comprehensive integration of treatment is highly needed, because the pathophysiology of CS encompasses disturbance from molecular, cellular, organic to systemic levels. The first integration is combination of multiple blockers or modulators of CS signaling. One good example is the treatment for HLH, in which CS signaling is targeted in various ways.^553^ The treatment of HLH basically comprises two phases: controlling overwhelmed CS with chemotherapy to eliminate activated T cells and inhibit inflammatory cytokine production, and subsequently replacing the defective immune system by allo-HSCT.^554^ Additionally, IFN-γ antibody (emapalumab) is also applied for HLH treatment. Other target therapies such as JAK inhibitors, and CD20 antibody are also under investigation for treating HLH.^553^ The second integration is combination of multiple forms of medical intervention, including drug therapies, respiratory support with mechanical ventilation, circulatory support with IABP/ECMO, renal support with CRRT etc. This applies to CS control of all the clinical situations, such as FM, ARDS, HLH, aGVHD, CAR-T related CRS.^270,446,555,556^ The third integration is multidisciplinary cooperation. Because CS is a systemic inflammatory state, multiple organ might suffer to different extents. Multidisciplinary teamwork contributes to early recognition and better preservation of organ function.^557^

Identifying the key mediator of specific conditions provides information for diagnostic biomarkers and target therapies. Several common pathways, such as TLRs, JAK/STAT, NLRP3 inflammasome, and NETs, are activated to produce massive inflammatory cytokines across various CS scenarios. The dominant signaling pathway and key inflammatory mediator are different in each disease. Applying improper targeted therapy may lead to ineffective treatment or even harmful consequence. For example, in rheumatic diseases and MAS, TNF blocking is effective. However, the effect of TNF blocking in HLH is yet uncertain. There were even reports suggesting that anti-TNF therapy could indirectly induce HLH or worsen inflammation.^558^ Therefore, it is of great importance to identify the key mediator.

Pharmacological evolution for more selective therapy with less off-target effects makes the treatment safer. A well-studied example is JAK- inhibitors. The first generation JAK inhibitors have been approved for controlling CS in aGVHD and rheumatic diseases and showed efficacy in treating ARDS.^559^ However, due to insufficient selectivity, these inhibitors also have off-target effects. They increase the risk of severe and opportunistic infections, and lead to anemia and decreased counts of lymphocytes, NK cells, neutrophils and platelets.^560,561^ Therefore, the next generation of JAK inhibitors with better selectivity have been under exploration. Their therapeutic efficacy and safety are to be investigated.

The future of CS management lies in a multifaceted approach that combines cutting-edge technologies, such as omics, artificial intelligence (AI), targeted drug delivery, gene editing, and biomaterials, with advanced life support systems and organ replacement therapies. These innovations will lead to more effective, precise, and personalized treatments, offering hope for improved outcomes in patients suffering from CS.

For deeper insight into CS, advancements in omics technologies will provide an unprecedented view of the complex molecular networks driving CS. The high-throughput omics technologies, such as genomics, transcriptomics, proteomics, and metabolomics, allow for the identification of specific cytokines, signaling pathways, and cellular processes involved in the initiation and escalation of the inflammatory response. By combining these technologies with single-cell RNA sequencing (scRNA-seq), researchers can now map the single-cell atlas of CS. The detailed molecular profiling provided by these techniques offers a better understanding of the immune cell populations and their roles in driving inflammation, enabling the discovery of novel therapeutic targets for more precise intervention.

For early diagnosis of CS, AI and deep machine learning models hold great potential in predicting CS. By analyzing vast datasets from electronic health records, biomarkers, and clinical imaging, AI models can identify early signs of CS development before it progresses to life-threatening stages. These predictive models can be integrated into early-warning systems, enabling clinicians to take timely action. Additionally, machine learning algorithms can help in stratifying patients based on their likelihood of developing severe CS, thus allowing for personalized treatment regimens.

For better treatment of CS, novel treatments might offer new options besides drug therapies. The upgrading of biomaterials may lead to the generation of new materials with broad adsorption spectrum of cytokines to cool down CS directly and rapidly. In addition, targeted drug delivery systems have the potential to revolutionize the treatment of CS. Technologies such as liposomes, nanoparticles, and microspheres can be engineered to deliver anti-cytokine agents directly to inflamed tissues or immune cells, ensuring high local concentrations of drugs while minimizing systemic toxicity. These delivery systems can be activated by specific stimuli (such as pH or temperature changes) that are characteristic of the inflammatory microenvironment, allowing for precise targeting of cytokines involved in the storm. Furthermore, the emerging field of gene editing using technologies like CRISPR-Cas9 holds tremendous potential for treating CS. One promising approach is the genetic modification of MSCT to enhance its anti-inflammatory properties. Gene editing could be used to modify MSCTs to express cytokine inhibitors or suppress inflammatory pathways, thereby reducing the severity of CS.

In cases of severe CS, advanced life support technologies and organ replacement are critical in sustaining life. Based on the current life support technologies such as ECMO, ventilation, and CRRT, a new generation of life support may aim to improve treatment efficacy and reduce side effects. Integrating these systems with real-time monitoring of inflammatory biomarkers will enable more personalized and responsive care. For patients suffering end stage organ failure with severe CS, organ replacement could be life-saving. To overcome the lack of organ from human donors, organ replacement from other species and artificial organs offers new choice. There has been exploration in transplanting pig heart to patients with end stage heart failure.^562^ The integration of organ-on-chip technologies and bioprinting would eventually enable the creation of functional organs for transplantation or as temporary supports until donor organs become available.

While the development of new therapies for CS is exciting, it is essential to remain vigilant regarding the potential for therapeutic-induced CS. Some treatments, particularly immune-modulating therapies or CAR-T cell therapies, can inadvertently trigger excessive CS in certain individuals. Monitoring for early signs of CS in patients undergoing these treatments is crucial to prevent harm and ensure the safe administration of novel therapies. As research in these fields progresses, we are likely to see significant improvements in managing this life-threatening inflammatory response.

## References

[1] Fajgenbaum, D. C. & June, C. H. Cytokine storm. *N. Engl. J. Med.* **383**, 2255–2273 (2020). 33264547 10.1056/NEJMra2026131 PMC7727315

[2] Yang, L. et al. The signal pathways and treatment of cytokine storm in COVID-19. *Signal Transduct. Target. Ther.* **6**, 255 (2021). 34234112 10.1038/s41392-021-00679-0 PMC8261820

[3] Flemming, A. Sleep deprivation whips up cytokine storm. *Nat. Rev. Immunol.* **24**, 2 (2024). 38082102 10.1038/s41577-023-00980-9

[4] Kang, H. et al. Neutrophil-macrophage communication via extracellular vesicle transfer promotes itaconate accumulation and ameliorates cytokine storm syndrome. *Cell. Mol. Immunol.* **21**, 689–706 (2024). 38745069 10.1038/s41423-024-01174-6 PMC11637192

[5] Alarabei, A. A. et al. Immunomodulating phytochemicals: an insight into their potential use in cytokine storm situations. *Adv. Pharm. Bull.* **14**, 105–119 (2024). 38585461 10.34172/apb.2024.001 PMC10997936

[6] Lerro, S. J., Schmerer, F. & Rapalski, A. J. Unusually severe cases seen in an explosive epidemic of influenza and influenza-like syndrome. *US Armed Forces Med. J.* **9**, 487–497 (1958). 13543966

[7] Alegre, M. L. et al. Cytokine release syndrome induced by the 145-2C11 anti-CD3 monoclonal antibody in mice: prevention by high doses of methylprednisolone. *J. Immunol.* **146**, 1184–1191 (1991). 1825107

[8] McCarthy, P. L. Jr. et al. Inhibition of interleukin-1 by an interleukin-1 receptor antagonist prevents graft-versus-host disease. *Blood* **78**, 1915–1918 (1991). 1832996

[9] Hervé, P. et al. Phase I-II trial of a monoclonal anti-tumor necrosis factor alpha antibody for the treatment of refractory severe acute graft-versus-host disease. *Blood* **79**, 3362–3368 (1992). 1596576

[10] Ferrara, J. L., Abhyankar, S. & Gilliland, D. G. Cytokine storm of graft-versus-host disease: a critical effector role for interleukin-1. *Transpl. Proc.* **25**, 1216–1217 (1993). 8442093

[11] Karki, R. & Kanneganti, T. D. The ‘cytokine storm’: molecular mechanisms and therapeutic prospects. *Trends Immunol.* **42**, 681–705 (2021). 34217595 10.1016/j.it.2021.06.001 PMC9310545

[12] Baik, A. H. et al. Mechanisms of Cardiovascular Toxicities Associated With Immunotherapies. *Circ. Res.* **128**, 1780–1801 (2021). 33934609 10.1161/CIRCRESAHA.120.315894 PMC8159878

[13] Zhou, T., Su, T. T., Mudianto, T. & Wang, J. Immune asynchrony in COVID-19 pathogenesis and potential immunotherapies. *J. Exp. Med.* **217**, e20200674 (2020). 32910820 10.1084/jem.20200674 PMC7481961

[14] Li, G., Wang, H. & Meftahpour, V. Overall review of curative impact and barriers of CAR-T cells in osteosarcoma. *EXCLI J.* **23**, 364–383 (2024). 38655095 10.17179/excli2023-6760 PMC11036068

[15] Jin, H., Li, M., Jeong, E., Castro-Martinez, F. & Zuker, C. S. A body-brain circuit that regulates body inflammatory responses. *Nature* **630**, 695–703 (2024). 38692285 10.1038/s41586-024-07469-y PMC11186780

[16] Rubin, T. S. et al. Perforin and CD107a testing is superior to NK cell function testing for screening patients for genetic HLH. *Blood* **129**, 2993–2999 (2017). 28270454 10.1182/blood-2016-12-753830 PMC5766842

[17] Fardet, L. et al. Development and validation of the HScore, a score for the diagnosis of reactive hemophagocytic syndrome. *Arthritis Rheumatol.* **66**, 2613–2620 (2014). 24782338 10.1002/art.38690

[18] Minoia, F. et al. Development and initial validation of the MS score for diagnosis of macrophage activation syndrome in systemic juvenile idiopathic arthritis. *Ann. Rheum. Dis.* **78**, 1357–1362 (2019). 31296501 10.1136/annrheumdis-2019-215211

[19] Lin, Z., Zuo, J. & He, S. Surrogate cytokine agonists: promising agents against COVID-19. *Signal Transduct. Target. Ther.* **7**, 150 (2022). 35523768 10.1038/s41392-022-01015-w PMC9072766

[20] Li, H. et al. Self-recruited neutrophils trigger over-activated innate immune response and phenotypic change of cardiomyocytes in fulminant viral myocarditis. *Cell Discov.* **9**, 103 (2023). 37816761 10.1038/s41421-023-00593-5 PMC10564723

[21] Sousa, P. M. B. et al. Fatal myocarditis following COVID-19 mRNA immunization: a case report and differential diagnosis review. *Vaccines* **12**, 194 (2024). 38400177 10.3390/vaccines12020194 PMC10891853

[22] Kullberg, R. F. J., de Brabander, J., Boers, L. S., Bos, L. D. J. & Wiersinga, W. J. Reply to: microbial burden-associated cytokine storm may explain non-resolving ARDS in COVID-19 patients. *Am. J. Respir. Crit. Care Med.* **206**, 1183–1184 (2022). 35867884 10.1164/rccm.202207-1346LE PMC9704841

[23] Gibson, B. H. Y. et al. Plasmin drives burn-induced systemic inflammatory response syndrome. *JCI Insight* **6**, e154439 (2021). 34877937 10.1172/jci.insight.154439 PMC8675186

[24] Gentile, L. F. & Moldawer, L. L. HMGB1 as a therapeutic target for sepsis: it’s all in the timing! *Expert Opin. Ther. Targets* **18**, 243–245 (2014). 24479494 10.1517/14728222.2014.883380 PMC5119516

[25] Dettmer-Monaco, V. et al. Gene editing of hematopoietic stem cells restores T-cell response in familial hemophagocytic lymphohistiocytosis. *J. Allergy Clin. Immunol.* **153**, 243–255.e214 (2024). 37595758 10.1016/j.jaci.2023.08.003

[26] Sato, A. et al. Inhibition of plasmin attenuates murine acute graft-versus-host disease mortality by suppressing the matrix metalloproteinase-9-dependent inflammatory cytokine storm and effector cell trafficking. *Leukemia* **29**, 145–156 (2015). 24791857 10.1038/leu.2014.151

[27] Zhu, C. et al. Rationally designed approaches to augment CAR-T therapy for solid tumor treatment. *Bioact. Mater.* **33**, 377–395 (2024). 38059121 10.1016/j.bioactmat.2023.11.002 PMC10696433

[28] Yang, S. et al. Neutrophil activation and clonal CAR-T re-expansion underpinning cytokine release syndrome during ciltacabtagene autoleucel therapy in multiple myeloma. *Nat. Commun.* **15**, 360 (2024). 38191582 10.1038/s41467-023-44648-3 PMC10774397

[29] Tian, J. et al. Clinical characteristics and risk factors associated with COVID-19 disease severity in patients with cancer in Wuhan, China: a multicentre, retrospective, cohort study. *Lancet Oncol.* **21**, 893–903 (2020). 32479790 10.1016/S1470-2045(20)30309-0 PMC7259911

[30] Chu, J. et al. Clinical characteristics of 54 medical staff with COVID-19: A retrospective study in a single center in Wuhan, China. *J. Med. Virol.* **92**, 807–813 (2020). 32222986 10.1002/jmv.25793 PMC7228263

[31] Wang, F. et al. The laboratory tests and host immunity of COVID-19 patients with different severity of illness. *JCI insight* **5**, e137799 (2020). 32324595 10.1172/jci.insight.137799 PMC7259533

[32] Ning, Q. et al. The mechanism underlying extrapulmonary complications of the coronavirus disease 2019 and its therapeutic implication. *Signal Transduct. Target. Ther.* **7**, 57 (2022). 35197452 10.1038/s41392-022-00907-1 PMC8863906

[33] Chen, T. et al. Clinical characteristics of 113 deceased patients with coronavirus disease 2019: retrospective study. *BMJ* **368**, m1091 (2020). 32217556 10.1136/bmj.m1091 PMC7190011

[34] Wang, J. et al. Soluble ST2 is a sensitive and specific biomarker for fulminant myocarditis. *J. Am. Heart Assoc.* **11**, e024417 (2022). 35377184 10.1161/JAHA.121.024417 PMC9075487

[35] Tang, Y. et al. Excessive IL-10 and IL-18 trigger hemophagocytic lymphohistiocytosis-like hyperinflammation and enhanced myelopoiesis. *J. Allergy Clin. Immunol.* **150**, 1154–1167 (2022). 35792218 10.1016/j.jaci.2022.06.017 PMC9643619

[36] Gao, Y. et al. A living neutrophil Biorobot synergistically blocks multifaceted inflammatory pathways in macrophages to effectively neutralize cytokine storm. *Chem. Sci.* **15**, 2243–2256 (2024). 38332816 10.1039/d3sc03438k PMC10848682

[37] Gao, Q. et al. Autologous cryo-shocked neutrophils enable targeted therapy of sepsis via broad-spectrum neutralization of pro-inflammatory cytokines and endotoxins. *Front. Chem.* **12**, 1359946 (2024). 38449477 10.3389/fchem.2024.1359946 PMC10914999

[38] Pu, Y. et al. Multi-centers experience using therapeutic plasma exchange for corticosteroid/tocilizumab-refractory cytokine release syndrome following CAR-T therapy. *Int Immunopharmacol.* **130**, 111761 (2024). 38422769 10.1016/j.intimp.2024.111761

[39] Lin, Y. T. et al. JAK2 phosphorylation signals and their associated cytokines involved in chronic rhinosinusitis with nasal polyps and correlated with disease severity. *Biomolecules* **11**, 1059 (2021). 34356683 10.3390/biom11071059 PMC8301971

[40] Istanbullu, H., Coban, G., Turunc, E., Disel, C. & Debelec Butuner, B. Discovery of selective TYK2 inhibitors: design, synthesis, in vitro and in silico studies of promising hits with triazolopyrimidinone scaffold. *Bioorg. Chem.* **148**, 107430 (2024). 38728909 10.1016/j.bioorg.2024.107430

[41] Zhao, R. et al. The oncogenic mechanisms of the Janus kinase-signal transducer and activator of transcription pathway in digestive tract tumors. *Cell Commun. Signal* **22**, 68 (2024). 38273295 10.1186/s12964-023-01421-9 PMC10809652

[42] Gupta, S. et al. Inhibition of JAK-STAT pathway corrects salivary gland inflammation and interferon driven immune activation in Sjogren’s disease. *Ann. Rheum. Dis.* **83**, 1034–1047 (2024). 38527764 10.1136/ard-2023-224842 PMC11250564

[43] Ahmed, E. A., Alzahrani, A. M., Abdelsalam, S. A. & Ibrahim, H. M. Flavipin from fungi as a potential inhibitor of rheumatoid arthritis signaling molecules. *Inflammopharmacology* **32**, 1171–1186 (2024). 38349589 10.1007/s10787-024-01429-8

[44] Xiang, J. et al. Design, synthesis, and pharmacological evaluation of quinazoline derivatives as novel and potent pan-JAK inhibitors. *Bioorg. Chem.* **140**, 106765 (2023). 37582330 10.1016/j.bioorg.2023.106765

[45] Telliez, J. B. et al. Discovery of a JAK3-selective inhibitor: functional differentiation of JAK3-selective inhibition over pan-JAK or JAK1-selective inhibition. *ACS Chem. Biol.* **11**, 3442–3451 (2016). 27791347 10.1021/acschembio.6b00677

[46] Xue, C. et al. Evolving cognition of the JAK-STAT signaling pathway: autoimmune disorders and cancer. *Signal Transduct. Target. Ther.* **8**, 204 (2023). 37208335 10.1038/s41392-023-01468-7 PMC10196327

[47] Verhoeven, Y. et al. The potential and controversy of targeting STAT family members in cancer. *Semin. Cancer Biol.* **60**, 41–56 (2020). 31605750 10.1016/j.semcancer.2019.10.002

[48] Mahjoor, M. et al. Double-edged sword of JAK/STAT signaling pathway in viral infections: novel insights into virotherapy. *Cell Commun. Signal.* **21**, 272 (2023). 37784164 10.1186/s12964-023-01240-y PMC10544547

[49] Castelo-Soccio, L. et al. Protein kinases: drug targets for immunological disorders. *Nat. Rev. Immunol.* **23**, 787–806 (2023). 37188939 10.1038/s41577-023-00877-7 PMC10184645

[50] Kang, S. et al. Gp130-HIF1alpha axis-induced vascular damage is prevented by the short-term inhibition of IL-6 receptor signaling. *Proc. Natl Acad. Sci. USA* **121**, e2315898120 (2024). 38165930 10.1073/pnas.2315898120 PMC10786312

[51] Shafiey, S. I., Ahmed, K. A., Abo-Saif, A. A., Abo-Youssef, A. M. & Mohamed, W. R. Galantamine mitigates testicular injury and disturbed spermatogenesis in adjuvant arthritic rats via modulating apoptosis, inflammatory signals, and IL-6/JAK/STAT3/SOCS3 signaling. *Inflammopharmacology* **32**, 405–418 (2024). 37429998 10.1007/s10787-023-01268-z PMC10907493

[52] Pradhan, R. et al. Nano formulated Resveratrol inhibits PD-L1 in oral cancer cells by deregulating the association between tumor associated macrophages and cancer associated fibroblasts through IL-6/JAK2/STAT3 signaling axis. *J. Nutr. Biochem* **125**, 109568 (2024). 38185347 10.1016/j.jnutbio.2024.109568

[53] Feghhi-Najafabadi, S. & Shafiee, F. Recombinant production of a mutant form of soluble IL-6 receptor with inhibitory effects against interleukin-6. *Iran. J. Biotechnol.* **20**, e3021 (2022). 35891958 10.30498/ijb.2021.278685.3021 PMC9284238

[54] Rose-John, S., Jenkins, B. J., Garbers, C., Moll, J. M. & Scheller, J. Targeting IL-6 trans-signalling: past, present and future prospects. *Nat. Rev. Immunol.* **23**, 666–681 (2023). 37069261 10.1038/s41577-023-00856-y PMC10108826

[55] Cabrera-Rivera, G. L. et al. Increased TNF-alpha production in response to IL-6 in patients with systemic inflammation without infection. *Clin. Exp. Immunol.* **209**, 225–235 (2022). 35647912 10.1093/cei/uxac055 PMC9390847

[56] Riegler, L. L., Jones, G. P. & Lee, D. W. Current approaches in the grading and management of cytokine release syndrome after chimeric antigen receptor T-cell therapy. *Ther. Clin. Risk Manag* **15**, 323–335 (2019). 30880998 10.2147/TCRM.S150524 PMC6400118

[57] Garbers, C., Heink, S., Korn, T. & Rose-John, S. Interleukin-6: designing specific therapeutics for a complex cytokine. *Nat. Rev. Drug Discov.* **17**, 395–412 (2018). 29725131 10.1038/nrd.2018.45

[58] Kang, S., Tanaka, T., Narazaki, M. & Kishimoto, T. Targeting interleukin-6 signaling in clinic. *Immunity* **50**, 1007–1023 (2019). 30995492 10.1016/j.immuni.2019.03.026

[59] Baran, P. et al. The balance of interleukin (IL)-6, IL-6·soluble IL-6 receptor (sIL-6R), and IL-6·sIL-6R·sgp130 complexes allows simultaneous classic and trans-signaling. *J. Biol. Chem.* **293**, 6762–6775 (2018). 29559558 10.1074/jbc.RA117.001163 PMC5936821

[60] Johnson, D. E., O’Keefe, R. A. & Grandis, J. R. Targeting the IL-6/JAK/STAT3 signalling axis in cancer. *Nat. Rev. Clin. Oncol.* **15**, 234–248 (2018). 29405201 10.1038/nrclinonc.2018.8 PMC5858971

[61] Doherty, G. M. et al. Evidence for IFN-gamma as a mediator of the lethality of endotoxin and tumor necrosis factor-alpha. *J. Immunol.* **149**, 1666–1670 (1992). 1506688

[62] Karki, R. et al. Synergism of TNF-α and IFN-γ triggers inflammatory cell death, tissue damage, and mortality in SARS-CoV-2 Infection and cytokine shock syndromes. *Cell* **184**, 149–168.e117 (2021). 33278357 10.1016/j.cell.2020.11.025 PMC7674074

[63] Setiadi, A., Zoref-Lorenz, A., Lee, C. Y., Jordan, M. B. & Chen, L. Y. C. Malignancy-associated haemophagocytic lymphohistiocytosis. *Lancet Haematol* **9**, e217–e227 (2022). 35101205 10.1016/S2352-3026(21)00366-5

[64] Keenan, C. et al. Differential effects of JAK1 vs. JAK2 inhibition in mouse models of hemophagocytic lymphohistiocytosis. *Blood* **143**, 2386–2400 (2024). 38446698 10.1182/blood.2023021046 PMC11450374

[65] Meyer, L. K. et al. JAK/STAT pathway inhibition sensitizes CD8 T cells to dexamethasone-induced apoptosis in hyperinflammation. *Blood* **136**, 657–668 (2020). 32530039 10.1182/blood.2020006075 PMC7414590

[66] Cook, E. et al. Ruxolitinib pharmacokinetics and pharmacodynamics in children with acute and chronic graft-versus-host disease. *Transpl. Cell. Ther.* **30**, e521–e512 (2024). 528. 10.1016/j.jtct.2024.02.018 38401793

[67] Huarte, E. et al. Itacitinib (INCB039110), a JAK1 inhibitor, reduces cytokines associated with cytokine release syndrome induced by CAR T-cell therapy. *Clin. Cancer Res.* **26**, 6299–6309 (2020). 32998963 10.1158/1078-0432.CCR-20-1739 PMC7895329

[68] Leclercq, G. et al. JAK and mTOR inhibitors prevent cytokine release while retaining T cell bispecific antibody in vivo efficacy. *J. Immunother Cancer* **10**, e003766 (2022). 35064010 10.1136/jitc-2021-003766 PMC8785208

[69] Philips, R. L. et al. The JAK-STAT pathway at 30: much learned, much more to do. *Cell* **185**, 3857–3876 (2022). 36240739 10.1016/j.cell.2022.09.023 PMC9815833

[70] Dougan, M. Weighing antitumor immunity against life-threatening myocarditis from immune-checkpoint inhibitors. *Cancer Discov.* **13**, 1040–1042 (2023). 37139724 10.1158/2159-8290.CD-23-0199

[71] Soy, M., Atagündüz, P., Atagündüz, I. & Sucak, G. T. Hemophagocytic lymphohistiocytosis: a review inspired by the COVID-19 pandemic. *Rheumatol. Int.* **41**, 7–18 (2021). 32588191 10.1007/s00296-020-04636-y PMC7315691

[72] Keenan, C., Nichols, K. E. & Albeituni, S. Use of the JAK inhibitor ruxolitinib in the treatment of hemophagocytic lymphohistiocytosis. *Front. Immunol.* **12**, 614704 (2021). 33664745 10.3389/fimmu.2021.614704 PMC7923355

[73] Lange, A., Lange, J. & Jaskuła, E. Cytokine overproduction and immune system dysregulation in alloHSCT and COVID-19 patients. *Front. Immunol.* **12**, 658896 (2021). 34149697 10.3389/fimmu.2021.658896 PMC8206782

[74] Hill, G. R. & Koyama, M. Cytokines and costimulation in acute graft-versus-host disease. *Blood* **136**, 418–428 (2020). 32526028 10.1182/blood.2019000952 PMC7378458

[75] Abboud, R. et al. Insights into the role of the JAK/STAT signaling pathway in graft-versus-host disease. *Ther. Adv. Hematol.* **11**, 2040620720914489 (2020). 32537114 10.1177/2040620720914489 PMC7268158

[76] Harris, R. & Karimi, M. Dissecting the regulatory network of transcription factors in T cell phenotype/functioning during GVHD and GVT. *Front. Immunol.* **14**, 1194984 (2023). 37441063 10.3389/fimmu.2023.1194984 PMC10333690

[77] Bell, M. et al. Modular chimeric cytokine receptors with leucine zippers enhance the antitumour activity of CAR T cells via JAK/STAT signalling. *Nat. Biomed. Eng.* **8**, 380–396 (2024). 38036617 10.1038/s41551-023-01143-w PMC11587785

[78] Seif, F. et al. JAK inhibition as a new treatment strategy for patients with COVID-19. *Int. Arch. Allergy Immunol.* **181**, 467–475 (2020). 32392562 10.1159/000508247 PMC7270061

[79] Yasukawa, H. et al. The suppressor of cytokine signaling-1 (SOCS1) is a novel therapeutic target for enterovirus-induced cardiac injury. *J. Clin. Investig.* **111**, 469–478 (2003). 12588885 10.1172/JCI16491 PMC151924

[80] Pang, Q. et al. Regulation of the JAK/STAT signaling pathway: the promising targets for cardiovascular disease. *Biochem. Pharm.* **213**, 115587 (2023). 37187275 10.1016/j.bcp.2023.115587

[81] Hilfiker-Kleiner, D. et al. Signal transducer and activator of transcription 3 is required for myocardial capillary growth, control of interstitial matrix deposition, and heart protection from ischemic injury. *Circ. Res.* **95**, 187–195 (2004). 15192020 10.1161/01.RES.0000134921.50377.61

[82] Enomoto, D. et al. Cardiac-specific ablation of the STAT3 gene in the subacute phase of myocardial infarction exacerbated cardiac remodeling. *Am. J. Physiol. Heart Circ. Physiol.* **309**, H471–H480 (2015). 26055795 10.1152/ajpheart.00730.2014

[83] Hilfiker-Kleiner, D. et al. Continuous glycoprotein-130-mediated signal transducer and activator of transcription-3 activation promotes inflammation, left ventricular rupture, and adverse outcome in subacute myocardial infarction. *Circulation* **122**, 145–155 (2010). 20585009 10.1161/CIRCULATIONAHA.109.933127

[84] Li, L., Li, L., Xiao, L. & Shangguan, J. Progranulin ameliorates coxsackievirus-B3-induced viral myocarditis by downregulating Th1 and Th17 cells. *Exp. Cell Res.* **367**, 241–250 (2018). 29625085 10.1016/j.yexcr.2018.04.001

[85] Chang, H. et al. PPARalpha suppresses Th17 cell differentiation through IL-6/STAT3/RORgammat pathway in experimental autoimmune myocarditis. *Exp. Cell Res.* **375**, 22–30 (2019). 30557558 10.1016/j.yexcr.2018.12.005

[86] Tavares, P. S., Rocon-Albuquerque, R. Jr. & Leite-Moreira, A. F. Innate immune receptor activation in viral myocarditis: pathophysiologic implications. *Rev. Port. Cardiol.* **29**, 57–78 (2010). 20391900

[87] Manik, M. & Singh, R. K. Role of toll-like receptors in modulation of cytokine storm signaling in SARS-CoV-2-induced COVID-19. *J. Med. Virol.* **94**, 869–877 (2022). 34672376 10.1002/jmv.27405 PMC8662021

[88] Ciaston, I., Dobosz, E., Potempa, J. & Koziel, J. The subversion of toll-like receptor signaling by bacterial and viral proteases during the development of infectious diseases. *Mol. Asp. Med.* **88**, 101143 (2022). 10.1016/j.mam.2022.101143 PMC9924004 36152458

[89] Shafeghat, M., Kazemian, S., Aminorroaya, A., Aryan, Z. & Rezaei, N. Toll-like receptor 7 regulates cardiovascular diseases. *Int Immunopharmacol.* **113**, 109390 (2022). 36330918 10.1016/j.intimp.2022.109390

[90] Khawaja, A. & Bromage, D. I. The innate immune response in myocarditis. *Int. J. Biochem. Cell Biol.* **134**, 105973 (2021). 33831592 10.1016/j.biocel.2021.105973

[91] Yao, Y. et al. Surface translocation of ACE2 and TMPRSS2 upon TLR4/7/8 activation is required for SARS-CoV-2 infection in circulating monocytes. *Cell Discov.* **8**, 89 (2022). 36085197 10.1038/s41421-022-00453-8 PMC9462622

[92] Zhao, Y. et al. SARS-CoV-2 spike protein interacts with and activates TLR41. *Cell Res.* **31**, 818–820 (2021). 33742149 10.1038/s41422-021-00495-9 PMC7975240

[93] Naqvi, I. et al. DAMPs/PAMPs induce monocytic TLR activation and tolerance in COVID-19 patients; nucleic acid binding scavengers can counteract such TLR agonists. *Biomaterials* **283**, 121393 (2022). 35349874 10.1016/j.biomaterials.2022.121393 PMC8797062

[94] Gorbea, C. et al. A role for Toll-like receptor 3 variants in host susceptibility to enteroviral myocarditis and dilated cardiomyopathy. *J. Biol. Chem.* **285**, 23208–23223 (2010). 20472559 10.1074/jbc.M109.047464 PMC2906314

[95] Yang, Y. et al. The emerging role of Toll-like receptor 4 in myocardial inflammation. *Cell Death Dis.* **7**, e2234 (2016). 27228349 10.1038/cddis.2016.140 PMC4917669

[96] Becher, P. M. et al. Role of Toll-like receptors and interferon regulatory factors in different experimental heart failure models of diverse etiology: IRF7 as novel cardiovascular stress-inducible factor. *PLoS ONE* **13**, e0193844 (2018). 29538462 10.1371/journal.pone.0193844 PMC5851607

[97] Papageorgiou, A. P. & Heymans, S. Interactions between the extracellular matrix and inflammation during viral myocarditis. *Immunobiology* **217**, 503–510 (2012). 21907443 10.1016/j.imbio.2011.07.011

[98] Esfandiarei, M. & McManus, B. M. Molecular biology and pathogenesis of viral myocarditis. *Annu. Rev. Pathol.* **3**, 127–155 (2008). 18039131 10.1146/annurev.pathmechdis.3.121806.151534

[99] Fairweather, D. et al. Mast cells and innate cytokines are associated with susceptibility to autoimmune heart disease following coxsackievirus B3 infection. *Autoimmunity* **37**, 131–145 (2004). 15293883 10.1080/0891693042000196200

[100] Corsten, M. F., Schroen, B. & Heymans, S. Inflammation in viral myocarditis: friend or foe? *Trends Mol. Med.* **18**, 426–437 (2012). 22726657 10.1016/j.molmed.2012.05.005

[101] Zhang, P., Cox, C. J., Alvarez, K. M. & Cunningham, M. W. Cutting edge: cardiac myosin activates innate immune responses through TLRs. *J. Immunol.* **183**, 27–31 (2009). 19535635 10.4049/jimmunol.0800861 PMC2720835

[102] Myers, J. M. et al. Cardiac myosin-Th17 responses promote heart failure in human myocarditis. *JCI insight* **1**, e85851 (2016). 27366791 10.1172/jci.insight.85851 PMC4924810

[103] Tillack, K., Breiden, P., Martin, R. & Sospedra, M. T lymphocyte priming by neutrophil extracellular traps links innate and adaptive immune responses. *J. Immunol.* **188**, 3150–3159 (2012). 22351936 10.4049/jimmunol.1103414

[104] Warnatsch, A., Ioannou, M., Wang, Q. & Papayannopoulos, V. Inflammation. Neutrophil extracellular traps license macrophages for cytokine production in atherosclerosis. *Science* **349**, 316–320 (2015). 26185250 10.1126/science.aaa8064 PMC4854322

[105] Al-Kuraishy, H. M. et al. Neutrophil Extracellular Traps (NETs) and Covid-19: a new frontiers for therapeutic modality. *Int Immunopharmacol.* **104**, 108516 (2022). 35032828 10.1016/j.intimp.2021.108516 PMC8733219

[106] Root-Bernstein, R. From co-infections to autoimmune disease via hyperactivated innate immunity: COVID-19 autoimmune coagulopathies, autoimmune myocarditis and multisystem inflammatory syndrome in children. *Int. J. Mol. Sci.* **24**, 3001 (2023). 36769320 10.3390/ijms24033001 PMC9917907

[107] Weckbach, L. T. et al. Midkine drives cardiac inflammation by promoting neutrophil trafficking and NETosis in myocarditis. *J. Exp. Med.* **216**, 350–368 (2019). 30647120 10.1084/jem.20181102 PMC6363424

[108] Swanson, K. V., Deng, M. & Ting, J. P. The NLRP3 inflammasome: molecular activation and regulation to therapeutics. *Nat. Rev. Immunol.* **19**, 477–489 (2019). 31036962 10.1038/s41577-019-0165-0 PMC7807242

[109] Bawazeer, A. O. et al. Interleukin-1beta exacerbates disease and is a potential therapeutic target to reduce pulmonary inflammation during severe influenza A virus infection. *Immunol. Cell Biol.* **99**, 737–748 (2021). 33834544 10.1111/imcb.12459 PMC8453884

[110] Laghlali, G., Lawlor, K. E. & Tate, M. D. Die another way: interplay between influenza A virus, inflammation and cell death. *Viruses* **12**, 401 (2020). 32260457 10.3390/v12040401 PMC7232208

[111] Clark, J. T. et al. IL-18BP mediates the balance between protective and pathological immune responses to Toxoplasma gondii. *Cell Rep.* **42**, 112147 (2023). 36827187 10.1016/j.celrep.2023.112147 PMC10131179

[112] Gong, Q., Lin, Y., Lu, Z. & Xiao, Z. Microglia-astrocyte cross talk through IL-18/IL-18R signaling modulates migraine-like behavior in experimental models of migraine. *Neuroscience* **451**, 207–215 (2020). 33137409 10.1016/j.neuroscience.2020.10.019

[113] Mangan, M. S. J. et al. Targeting the NLRP3 inflammasome in inflammatory diseases. *Nat. Rev. Drug Discov.* **17**, 688 (2018). 30116046 10.1038/nrd.2018.149

[114] Broderick, L., De Nardo, D., Franklin, B. S., Hoffman, H. M. & Latz, E. The inflammasomes and autoinflammatory syndromes. *Annu Rev. Pathol.* **10**, 395–424 (2015). 25423351 10.1146/annurev-pathol-012414-040431

[115] Sarrauste de Menthière, C. et al. INFEVERS: the registry for FMF and hereditary inflammatory disorders mutations. *Nucleic acids Res.* **31**, 282–285 (2003). 12520003 10.1093/nar/gkg031 PMC165478

[116] Brydges, S. D. et al. Divergence of IL-1, IL-18, and cell death in NLRP3 inflammasomopathies. *J. Clin. Investig.* **123**, 4695–4705 (2013). 24084736 10.1172/JCI71543 PMC3809806

[117] Liu, X. et al. Mitochondrial calpain-1 activates NLRP3 inflammasome by cleaving ATP5A1 and inducing mitochondrial ROS in CVB3-induced myocarditis. *Basic Res. Cardiol.* **117**, 40 (2022). 35997820 10.1007/s00395-022-00948-1 PMC9399059

[118] Pappritz, K. et al. Colchicine prevents disease progression in viral myocarditis via modulating the NLRP3 inflammasome in the cardiosplenic axis. *ESC Heart Fail* **9**, 925–941 (2022). 35178861 10.1002/ehf2.13845 PMC8934990

[119] Ahn, M. et al. Dampened NLRP3-mediated inflammation in bats and implications for a special viral reservoir host. *Nat. Microbiol* **4**, 789–799 (2019). 30804542 10.1038/s41564-019-0371-3 PMC7096966

[120] Nieto-Torres, J. L. et al. Severe acute respiratory syndrome coronavirus E protein transports calcium ions and activates the NLRP3 inflammasome. *Virology* **485**, 330–339 (2015). 26331680 10.1016/j.virol.2015.08.010 PMC4619128

[121] Dutta, D., Liu, J. & Xiong, H. NLRP3 inflammasome activation and SARS-CoV-2-mediated hyperinflammation, cytokine storm and neurological syndromes. *Int. J. Physiol. PathoPhysiol. Pharm.* **14**, 138–160 (2022). PMC9301183 35891930

[122] Kang, Y. & Wang, Q. Potential therapeutic value of necroptosis inhibitor for the treatment of COVID-19. *Eur. J. Med. Res.* **27**, 283 (2022). 36494757 10.1186/s40001-022-00913-7 PMC9733178

[123] Oliva-Martin, M. J. et al. Caspase-8 inhibition represses initial human monocyte activation in septic shock model. *Oncotarget* **7**, 37456–37470 (2016). 27250033 10.18632/oncotarget.9648 PMC5122324

[124] Li, S. et al. SARS-CoV-2 triggers inflammatory responses and cell death through caspase-8 activation. *Signal Transduct. Target. Ther.* **5**, 235 (2020). 33037188 10.1038/s41392-020-00334-0 PMC7545816

[125] Yin, H. et al. Pyroptosis-inducing biomaterials pave the way for transformative antitumor immunotherapy. *Adv. Sci.* **11**, e2410336 (2024). 10.1002/advs.202410336 PMC11653674 39501932

[126] Wan, X. et al. H7N9 virus infection triggers lethal cytokine storm by activating gasdermin E-mediated pyroptosis of lung alveolar epithelial cells. *Natl Sci. Rev.* **9**, nwab137 (2022). 35087672 10.1093/nsr/nwab137 PMC8788236

[127] Wang, M., Chang, W., Zhang, L. & Zhang, Y. Pyroptotic cell death in SARS-CoV-2 infection: revealing its roles during the immunopathogenesis of COVID-19. *Int. J. Biol. Sci.* **18**, 5827–5848 (2022). 36263178 10.7150/ijbs.77561 PMC9576507

[128] Chen, W., Gullett, J. M., Tweedell, R. E. & Kanneganti, T. D. Innate immune inflammatory cell death: PANoptosis and PANoptosomes in host defense and disease. *Eur. J. Immunol.* **53**, e2250235 (2023). 36782083 10.1002/eji.202250235 PMC10423303

[129] Blanco-Melo, D. et al. Imbalanced host response to SARS-CoV-2 drives development of COVID-19. *Cell* **181**, 1036–1045.e1039 (2020). 32416070 10.1016/j.cell.2020.04.026 PMC7227586

[130] Cheung, P. H. et al. PB1-F2 protein of highly pathogenic influenza A (H7N9) virus selectively suppresses RNA-induced NLRP3 inflammasome activation through inhibition of MAVS-NLRP3 interaction. *J. Leukoc. Biol.* **108**, 1655–1663 (2020). 32386456 10.1002/JLB.4AB0420-694R

[131] Koh, C. H., Lee, S., Kwak, M., Kim, B. S. & Chung, Y. CD8 T-cell subsets: heterogeneity, functions, and therapeutic potential. *Exp. Mol. Med.* **55**, 2287–2299 (2023). 37907738 10.1038/s12276-023-01105-x PMC10689838

[132] Wik, J. A. & Skålhegg, B. S. T cell metabolism in infection. *Front. Immunol.* **13**, 840610 (2022). 35359994 10.3389/fimmu.2022.840610 PMC8964062

[133] Beringer, A., Noack, M. & Miossec, P. IL-17 in Chronic Inflammation: From Discovery To Targeting. *Trends Mol. Med.* **22**, 230–241 (2016). 26837266 10.1016/j.molmed.2016.01.001

[134] Schmidt, M. E. & Varga, S. M. The CD8 T cell response to respiratory virus infections. *Front. Immunol.* **9**, 678 (2018). 29686673 10.3389/fimmu.2018.00678 PMC5900024

[135] Sutton, V. R. et al. Initiation of apoptosis by granzyme B requires direct cleavage of bid, but not direct granzyme B-mediated caspase activation. *J. Exp. Med.* **192**, 1403–1414 (2000). 11085743 10.1084/jem.192.10.1403 PMC2193191

[136] Cron, R. Q. & Goyal, G. & Chatham, W. W. Cytokine storm syndrome. *Annu. Rev. Med.* **74**, 321–337 (2023). 36228171 10.1146/annurev-med-042921-112837

[137] Basu, R. et al. Cytotoxic T cells use mechanical force to potentiate target cell killing. *Cell* **165**, 100–110 (2016). 26924577 10.1016/j.cell.2016.01.021 PMC4808403

[138] Jenkins, M. R. et al. Failed CTL/NK cell killing and cytokine hypersecretion are directly linked through prolonged synapse time. *J. Exp. Med.* **212**, 307–317 (2015). 25732304 10.1084/jem.20140964 PMC4354371

[139] Kogawa, K. et al. Perforin expression in cytotoxic lymphocytes from patients with hemophagocytic lymphohistiocytosis and their family members. *Blood* **99**, 61–66 (2002). 11756153 10.1182/blood.v99.1.61

[140] Mackensen, A. et al. Phase I study of adoptive T-cell therapy using antigen-specific CD8+ T cells for the treatment of patients with metastatic melanoma. *J. Clin. Oncol.* **24**, 5060–5069 (2006). 17075125 10.1200/JCO.2006.07.1100

[141] Krishna, S. et al. Stem-like CD8 T cells mediate response of adoptive cell immunotherapy against human cancer. *Science* **370**, 1328–1334 (2020). 33303615 10.1126/science.abb9847 PMC8883579

[142] Gurney, M. et al. Features and factors associated with myeloid neoplasms after chimeric antigen receptor T-Cell therapy. *JAMA Oncol.* **10**, 532–535 (2024). 38386311 10.1001/jamaoncol.2023.7182 PMC10884941

[143] Abel, A. M., Yang, C., Thakar, M. S. & Malarkannan, S. Natural killer cells: development, maturation, and clinical utilization. *Front. Immunol.* **9**, 1869 (2018). 30150991 10.3389/fimmu.2018.01869 PMC6099181

[144] Myers, J. A. & Miller, J. S. Exploring the NK cell platform for cancer immunotherapy. *Nat. Rev. Clin. Oncol.* **18**, 85–100 (2021). 32934330 10.1038/s41571-020-0426-7 PMC8316981

[145] Walzer, T., Dalod, M., Robbins, S. H., Zitvogel, L. & Vivier, E. Natural-killer cells and dendritic cells: “l’union fait la force”. *Blood* **106**, 2252–2258 (2005). 15933055 10.1182/blood-2005-03-1154

[146] Kobayashi, H. et al. Role of trans-cellular IL-15 presentation in the activation of NK cell-mediated killing, which leads to enhanced tumor immunosurveillance. *Blood* **105**, 721–727 (2005). 15367431 10.1182/blood-2003-12-4187

[147] Klebanoff, C. A. et al. IL-15 enhances the in vivo antitumor activity of tumor-reactive CD8+ T cells. *Proc. Natl Acad. Sci. USA* **101**, 1969–1974 (2004). 14762166 10.1073/pnas.0307298101 PMC357036

[148] Grom, A. A., Horne, A. & De Benedetti, F. Macrophage activation syndrome in the era of biologic therapy. *Nat. Rev. Rheumatol.* **12**, 259–268 (2016). 27009539 10.1038/nrrheum.2015.179 PMC5851441

[149] Massilamany, C., Gangaplara, A., Steffen, D. & Reddy, J. Identification of novel mimicry epitopes for cardiac myosin heavy chain-alpha that induce autoimmune myocarditis in A/J mice. *Cell Immunol.* **271**, 438–449 (2011). 21939961 10.1016/j.cellimm.2011.08.013

[150] Epelman, S., Liu, P. P. & Mann, D. L. Role of innate and adaptive immune mechanisms in cardiac injury and repair. *Nat. Rev. Immunol.* **15**, 117–129 (2015). 25614321 10.1038/nri3800 PMC4669103

[151] Barin, J. G., Rose, N. R. & Cihakova, D. Macrophage diversity in cardiac inflammation: a review. *Immunobiology* **217**, 468–475 (2012). 21820754 10.1016/j.imbio.2011.06.009 PMC4292796

[152] Li, M. et al. Metabolism, metabolites, and macrophages in cancer. *J. Hematol. Oncol.* **16**, 80 (2023). 37491279 10.1186/s13045-023-01478-6 PMC10367370

[153] Poon, I. K., Lucas, C. D., Rossi, A. G. & Ravichandran, K. S. Apoptotic cell clearance: basic biology and therapeutic potential. *Nat. Rev. Immunol.* **14**, 166–180 (2014). 24481336 10.1038/nri3607 PMC4040260

[154] Bosurgi, L. et al. Macrophage function in tissue repair and remodeling requires IL-4 or IL-13 with apoptotic cells. *Science* **356**, 1072–1076 (2017). 28495875 10.1126/science.aai8132 PMC5556699

[155] Zhou, Z. et al. Type 2 cytokine signaling in macrophages protects from cellular senescence and organismal aging. *Immunity* **57**, 513–527.e516 (2024). 38262419 10.1016/j.immuni.2024.01.001

[156] Chen, M. M. et al. Polarization of tissue-resident TFH-like cells in human hepatoma bridges innate monocyte inflammation and M2b macrophage polarization. *Cancer Discov.* **6**, 1182–1195 (2016). 27531854 10.1158/2159-8290.CD-16-0329

[157] Shapouri-Moghaddam, A. et al. Macrophage plasticity, polarization, and function in health and disease. *J. Cell Physiol.* **233**, 6425–6440 (2018). 29319160 10.1002/jcp.26429 PMC13482560

[158] Lu, J. et al. Discrete functions of M2a and M2c macrophage subsets determine their relative efficacy in treating chronic kidney disease. *Kidney Int.* **84**, 745–755 (2013). 23636175 10.1038/ki.2013.135

[159] Wang, Q. et al. Fra-1 protooncogene regulates IL-6 expression in macrophages and promotes the generation of M2d macrophages. *Cell Res.* **20**, 701–712 (2010). 20386569 10.1038/cr.2010.52

[160] Sales, D. S. et al. Regulatory T-cell distribution within lung compartments in COPD. *COPD* **14**, 533–542 (2017). 28745532 10.1080/15412555.2017.1346069

[161] Maucourant, C. et al. Zika virus in the eye of the cytokine storm. *Eur. Cytokine Netw.* **30**, 74–81 (2019). 31957701 10.1684/ecn.2019.0433

[162] Chen, H. et al. Management of cytokine release syndrome related to CAR-T cell therapy. *Front. Med.* **13**, 610–617 (2019). 31571160 10.1007/s11684-019-0714-8

[163] Indalao, I. L., Sawabuchi, T., Takahashi, E. & Kido, H. IL-1beta is a key cytokine that induces trypsin upregulation in the influenza virus-cytokine-trypsin cycle. *Arch. Virol.* **162**, 201–211 (2017). 27714503 10.1007/s00705-016-3093-3 PMC5225228

[164] Liew, P. X. & Kubes, P. The neutrophil’s role during health and disease. *Physiol. Rev.* **99**, 1223–1248 (2019). 30758246 10.1152/physrev.00012.2018

[165] Chan, L. et al. The roles of neutrophils in cytokine storms. *Viruses* **13**, 2318 (2021). 34835125 10.3390/v13112318 PMC8624379

[166] Rosales, C. Neutrophil: a cell with many roles in inflammation or several cell types? *Front. Physiol.* **9**, 113 (2018). 29515456 10.3389/fphys.2018.00113 PMC5826082

[167] Rosales, C. Neutrophils at the crossroads of innate and adaptive immunity. *J. Leukoc. Biol.* **108**, 377–396 (2020). 32202340 10.1002/JLB.4MIR0220-574RR

[168] Rungelrath, V., Kobayashi, S. D. & DeLeo, F. R. Neutrophils in innate immunity and systems biology-level approaches. *Wiley Interdiscip. Rev. Syst. Biol. Med.* **12**, e1458 (2020). 31218817 10.1002/wsbm.1458 PMC6898734

[169] Cassatella, M. A., Östberg, N. K., Tamassia, N. & Soehnlein, O. Biological roles of neutrophil-derived granule proteins and cytokines. *Trends Immunol.* **40**, 648–664 (2019). 31155315 10.1016/j.it.2019.05.003

[170] Tamassia, N. et al. Cytokine production by human neutrophils: revisiting the “dark side of the moon”. *Eur. J. Clin. Investig.* **48**, e12952 (2018). 29772063 10.1111/eci.12952

[171] Carai, P. et al. Neutrophil inhibition improves acute inflammation in a murine model of viral myocarditis. *Cardiovasc. Res.* **118**, 3331–3345 (2023). 35426438 10.1093/cvr/cvac052 PMC9847559

[172] Jung, Y. S. et al. Wnt5a stimulates chemotactic migration and chemokine production in human neutrophils. *Exp. Mol. Med.* **45**, e27 (2013). 23764954 10.1038/emm.2013.48 PMC3701286

[173] Soehnlein, O. et al. Neutrophil primary granule proteins HBP and HNP1-3 boost bacterial phagocytosis by human and murine macrophages. *J. Clin. Investig.* **118**, 3491–3502 (2008). 18787642 10.1172/JCI35740 PMC2532980

[174] Ichikawa, A. et al. CXCL10-CXCR3 enhances the development of neutrophil-mediated fulminant lung injury of viral and nonviral origin. *Am. J. Respir. Crit. Care Med.* **187**, 65–77 (2013). 23144331 10.1164/rccm.201203-0508OC PMC3927876

[175] Scannell, M. et al. Annexin-1 and peptide derivatives are released by apoptotic cells and stimulate phagocytosis of apoptotic neutrophils by macrophages. *J. Immunol.* **178**, 4595–4605 (2007). 17372018 10.4049/jimmunol.178.7.4595

[176] Soehnlein, O. & Lindbom, L. Phagocyte partnership during the onset and resolution of inflammation. *Nat. Rev. Immunol.* **10**, 427–439 (2010). 20498669 10.1038/nri2779

[177] Gschwandtner, M., Derler, R. & Midwood, K. S. More than just attractive: how CCL2 influences myeloid cell behavior beyond chemotaxis. *Front. Immunol.* **10**, 2759 (2019). 31921102 10.3389/fimmu.2019.02759 PMC6923224

[178] Kreisel, D. et al. In vivo two-photon imaging reveals monocyte-dependent neutrophil extravasation during pulmonary inflammation. *Proc. Natl Acad. Sci. USA* **107**, 18073–18078 (2010). 20923880 10.1073/pnas.1008737107 PMC2964224

[179] Zhang, R. et al. Neutrophil autophagy and NETosis in COVID-19: perspectives. *Autophagy* **19**, 758–767 (2023). 35951555 10.1080/15548627.2022.2099206 PMC9980466

[180] Barnes, B. J. et al. Targeting potential drivers of COVID-19: neutrophil extracellular traps. *J. Exp. Med.* **217**, e20200652 (2020). 32302401 10.1084/jem.20200652 PMC7161085

[181] Zhang, B. et al. Immune phenotyping based on the neutrophil-to-lymphocyte ratio and IgG level predicts disease severity and outcome for patients With COVID-19. *Front. Mol. Biosci.* **7**, 157 (2020). 32719810 10.3389/fmolb.2020.00157 PMC7350507

[182] Shi, H. et al. Neutrophil calprotectin identifies severe pulmonary disease in COVID-19. *J. Leukoc. Biol.* **109**, 67–72 (2021). 32869342 10.1002/JLB.3COVCRA0720-359R PMC7902293

[183] Obermayer, A. et al. Neutrophil extracellular traps in fatal COVID-19-associated lung injury. *Dis. Markers* **2021**, 5566826 (2021). 34367376 10.1155/2021/5566826 PMC8337148

[184] Zhao, L. & Fu, Z. Roles of host immunity in viral myocarditis and dilated cardiomyopathy. *J. Immunol. Res.* **2018**, 5301548 (2018). 29854842 10.1155/2018/5301548 PMC5964556

[185] Cen, Z. et al. The role of B cells in regulation of Th cell differentiation in coxsackievirus B3-induced acute myocarditis. *Inflammation* **44**, 1949–1960 (2021). 33961174 10.1007/s10753-021-01472-5

[186] Luo, Y. et al. Stem cell factor/mast cell/CCL2/monocyte/macrophage axis promotes Coxsackievirus B3 myocarditis and cardiac fibrosis by increasing Ly6C(high) monocyte influx and fibrogenic mediators production. *Immunology* **167**, 590–605 (2022). 36054617 10.1111/imm.13556

[187] Gilles, S., Zahler, S., Welsch, U., Sommerhoff, C. P. & Becker, B. F. Release of TNF-alpha during myocardial reperfusion depends on oxidative stress and is prevented by mast cell stabilizers. *Cardiovasc. Res.* **60**, 608–616 (2003). 14659806 10.1016/j.cardiores.2003.08.016

[188] He, A. et al. Mast cell-deficiency protects mice from streptozotocin-induced diabetic cardiomyopathy. *Transl. Res.* **208**, 1–14 (2019). 30738862 10.1016/j.trsl.2019.01.005 PMC6527494

[189] O’Sullivan, J. A. & Bochner, B. S. Eosinophils and eosinophil-associated diseases: an update. *J. Allergy Clin. Immunol.* **141**, 505–517 (2018). 29045815 10.1016/j.jaci.2017.09.022 PMC5803328

[190] Acharya, K. R. & Ackerman, S. J. Eosinophil granule proteins: form and function. *J. Biol. Chem.* **289**, 17406–17415 (2014). 24802755 10.1074/jbc.R113.546218 PMC4067173

[191] Abdala-Valencia, H. et al. Shaping eosinophil identity in the tissue contexts of development, homeostasis, and disease. *J. Leukoc. Biol.* **104**, 95–108 (2018). 29656559 10.1002/JLB.1MR1117-442RR PMC6013365

[192] McBrien, C. N. & Menzies-Gow, A. The biology of eosinophils and their role in asthma. *Front. Med.* **4**, 93 (2017). 10.3389/fmed.2017.00093 PMC5491677 28713812

[193] Robinson, D. et al. Revisiting Type 2-high and Type 2-low airway inflammation in asthma: current knowledge and therapeutic implications. *Clin. Exp. Allergy* **47**, 161–175 (2017). 28036144 10.1111/cea.12880

[194] Wechsler, M. E. et al. Eosinophils in health and disease: a state-of-the-art review. *Mayo Clin. Proc.* **96**, 2694–2707 (2021). 34538424 10.1016/j.mayocp.2021.04.025

[195] Kang, S. & Kishimoto, T. Interplay between interleukin-6 signaling and the vascular endothelium in cytokine storms. *Exp. Mol. Med.* **53**, 1116–1123 (2021). 34253862 10.1038/s12276-021-00649-0 PMC8273570

[196] Bonaventura, A. et al. Endothelial dysfunction and immunothrombosis as key pathogenic mechanisms in COVID-19. *Nat. Rev. Immunol.* **21**, 319–329 (2021). 33824483 10.1038/s41577-021-00536-9 PMC8023349

[197] Escher, R., Breakey, N. & Lämmle, B. Severe COVID-19 infection associated with endothelial activation. *Thromb. Res.* **190**, 62 (2020). 32305740 10.1016/j.thromres.2020.04.014 PMC7156948

[198] Conway, E. M. et al. Understanding COVID-19-associated coagulopathy. *Nat. Rev. Immunol.* **22**, 639–649 (2022). 35931818 10.1038/s41577-022-00762-9 PMC9362465

[199] Ramlall, V. et al. Immune complement and coagulation dysfunction in adverse outcomes of SARS-CoV-2 infection. *Nat. Med.* **26**, 1609–1615 (2020). 32747830 10.1038/s41591-020-1021-2 PMC7809634

[200] Middleton, E. A. et al. Neutrophil extracellular traps contribute to immunothrombosis in COVID-19 acute respiratory distress syndrome. *Blood* **136**, 1169–1179 (2020). 32597954 10.1182/blood.2020007008 PMC7472714

[201] Kazanski, V., Mitrokhin, V. M., Mladenov, M. I. & Kamkin, A. G. Cytokine effects on mechano-induced electrical activity in atrial myocardium. *Immunol. Investig.* **46**, 22–37 (2017). 27617892 10.1080/08820139.2016.1208220

[202] Cavalli, G. et al. Treating life-threatening myocarditis by blocking interleukin-1. *Crit. Care Med.* **44**, e751–e754 (2016). 27031379 10.1097/CCM.0000000000001654

[203] Meneghel, A. et al. Case report: life-threatening macrophage activation syndrome with fulminant myocarditis successfully rescued by high dose intravenous anakinra. *Front. Pediatr.* **8**, 635080 (2020). 33537271 10.3389/fped.2020.635080 PMC7848179

[204] Cavalli, G. et al. Interleukin-1 receptor blockade rescues myocarditis-associated end-stage heart failure. *Front. Immunol.* **8**, 131 (2017). 28232838 10.3389/fimmu.2017.00131 PMC5298961

[205] Dorn, G. W. 2nd Inflame on!: mitochondrial escape provokes cytokine storms that doom the heart. *Circ. Res.* **111**, 271–273 (2012). 22821906 10.1161/CIRCRESAHA.112.275867

[206] Keshavarz-Bahaghighat, H., Darwesh, A. M., Sosnowski, D. K. & Seubert, J. M. Mitochondrial dysfunction and inflammaging in heart failure: novel roles of CYP-derived epoxylipids. *Cells* **9**, 1565 (2020). 32604981 10.3390/cells9071565 PMC7408578

[207] Nie, J. et al. Activation of CaMKII via ER-stress mediates coxsackievirus B3-induced cardiomyocyte apoptosis. *Cell Biol. Int.* **44**, 488–498 (2020). 31631456 10.1002/cbin.11249

[208] Peretto, G. et al. Arrhythmias in myocarditis: state of the art. *Heart Rhythm* **16**, 793–801 (2019). 30476544 10.1016/j.hrthm.2018.11.024

[209] Hang, W., Chen, C., Seubert, J. M. & Wang, D. W. Fulminant myocarditis: a comprehensive review from etiology to treatments and outcomes. *Signal Transduct. Target. Ther.* **5**, 287 (2020). 33303763 10.1038/s41392-020-00360-y PMC7730152

[210] Tian, C. J., Zhang, J. H., Liu, J., Ma, Z. & Zhen, Z. Ryanodine receptor and immune-related molecules in diabetic cardiomyopathy. *ESC Heart Fail* **8**, 2637–2646 (2021). 34013670 10.1002/ehf2.13431 PMC8318495

[211] Zhang, A., Zhang, H. & Wu, S. Immunomodulation by atorvastatin upregulates expression of gap junction proteins in coxsackievirus B3 (CVB3)-induced myocarditis. *Inflamm. Res.* **59**, 255–262 (2010). 19774449 10.1007/s00011-009-0093-8

[212] Casella, M. et al. Diagnostic yield of electroanatomic voltage mapping in guiding endomyocardial biopsies. *Circulation* **142**, 1249–1260 (2020). 32791857 10.1161/CIRCULATIONAHA.120.046900

[213] Sawamura, A. et al. Prognostic Value of electrocardiography in patients with fulminant myocarditis supported by percutaneous venoarterial extracorporeal membrane oxygenation- analysis from the CHANGE PUMP study. *Circ. J.* **82**, 2089–2095 (2018). 29863096 10.1253/circj.CJ-18-0136

[214] Manosalva, C. et al. Role of lactate in inflammatory processes: friend or foe. *Front. Immunol.* **12**, 808799 (2021). 35095895 10.3389/fimmu.2021.808799 PMC8795514

[215] Kaur, J. et al. Protective effect of olopatadine hydrochloride against LPS-induced acute lung injury: via targeting NF-kappaB signaling pathway. *Inflammopharmacology* **32**, 603–627 (2024). 37847473 10.1007/s10787-023-01353-3

[216] Tanaka, T., Narazaki, M. & Kishimoto, T. Immunotherapeutic implications of IL-6 blockade for cytokine storm. *Immunotherapy* **8**, 959–970 (2016). 27381687 10.2217/imt-2016-0020

[217] Shieh, J. M., Tseng, H. Y., Jung, F., Yang, S. H. & Lin, J. C. Elevation of IL-6 and IL-33 levels in serum associated with lung fibrosis and skeletal muscle wasting in a bleomycin-induced lung injury mouse model. *Mediat. Inflamm.* **2019**, 7947596 (2019). 10.1155/2019/7947596 PMC6458868 31049028

[218] Sakaguchi, R. et al. Innate-like function of memory Th17 cells for enhancing endotoxin-induced acute lung inflammation through IL-22. *Int. Immunol.* **28**, 233–243 (2016). 26647405 10.1093/intimm/dxv070 PMC4888348

[219] Rejeski, K., Jain, M. D., Shah, N. N., Perales, M. A. & Subklewe, M. Immune effector cell-associated haematotoxicity after CAR T-cell therapy: from mechanism to management. *Lancet Haematol* **11**, e459–e470 (2024). 38734026 10.1016/S2352-3026(24)00077-2 PMC12413773

[220] Juluri, K. R. et al. Severe cytokine release syndrome is associated with hematologic toxicity following CD19 CAR T-cell therapy. *Blood Adv.* **6**, 2055–2068 (2022). 34666344 10.1182/bloodadvances.2020004142 PMC9006285

[221] Najafi, S., Ghanavat, M., Shahrabi, S., Gatavizadeh, Z. & Saki, N. The effect of inflammatory factors and their inhibitors on the hematopoietic stem cells fate. *Cell Biol. Int.* **45**, 900–912 (2021). 33386770 10.1002/cbin.11545

[222] Jahandideh, B. et al. The pro-Inflammatory cytokines effects on mobilization, self-renewal and differentiation of hematopoietic stem cells. *Hum. Immunol.* **81**, 206–217 (2020). 32139091 10.1016/j.humimm.2020.01.004

[223] He, H. et al. Aging-induced IL27Ra signaling impairs hematopoietic stem cells. *Blood* **136**, 183–198 (2020). 32305041 10.1182/blood.2019003910

[224] Yang, L. et al. IFN-gamma negatively modulates self-renewal of repopulating human hemopoietic stem cells. *J. Immunol.* **174**, 752–757 (2005). 15634895 10.4049/jimmunol.174.2.752

[225] Bogeska, R. et al. Inflammatory exposure drives long-lived impairment of hematopoietic stem cell self-renewal activity and accelerated aging. *Cell Stem Cell* **29**, 1273–1284.e1278 (2022). 35858618 10.1016/j.stem.2022.06.012 PMC9357150

[226] Rejeski, K. et al. Severe hematotoxicity after CD19 CAR-T therapy is associated with suppressive immune dysregulation and limited CAR-T expansion. *Sci. Adv.* **9**, eadg3919 (2023). 37738350 10.1126/sciadv.adg3919 PMC10516499

[227] Kitamura, W. et al. Bone marrow microenvironment disruption and sustained inflammation with prolonged haematologic toxicity after CAR T-cell therapy. *Br. J. Haematol.* **202**, 294–307 (2023). 36890790 10.1111/bjh.18747

[228] Mitchell, C. A. et al. Stromal niche inflammation mediated by IL-1 signalling is a targetable driver of haematopoietic ageing. *Nat. Cell Biol.* **25**, 30–41 (2023). 36650381 10.1038/s41556-022-01053-0 PMC7614279

[229] Malard, F., Holler, E., Sandmaier, B. M., Huang, H. & Mohty, M. Acute graft-versus-host disease. *Nat. Rev. Dis. Prim.* **9**, 27 (2023). 37291149 10.1038/s41572-023-00438-1

[230] Shono, Y. et al. Bone marrow graft-versus-host disease: early destruction of hematopoietic niche after MHC-mismatched hematopoietic stem cell transplantation. *Blood* **115**, 5401–5411 (2010). 20354171 10.1182/blood-2009-11-253559

[231] Yao, Y. et al. Dysfunction of bone marrow vascular niche in acute graft-versus-host disease after MHC-haploidentical bone marrow transplantation. *PLoS ONE* **9**, e104607 (2014). 25119573 10.1371/journal.pone.0104607 PMC4131885

[232] Paolino, J., Berliner, N. & Degar, B. Hemophagocytic lymphohistiocytosis as an etiology of bone marrow failure. *Front. Oncol.* **12**, 1016318 (2022). 36387094 10.3389/fonc.2022.1016318 PMC9647152

[233] Shimabukuro-Vornhagen, A. et al. Cytokine release syndrome. *J. Immunother. Cancer* **6**, 56 (2018). 29907163 10.1186/s40425-018-0343-9 PMC6003181

[234] Alonso, J., Sánchez de Miguel, L., Montón, M., Casado, S. & López-Farré, A. Endothelial cytosolic proteins bind to the 3’ untranslated region of endothelial nitric oxide synthase mRNA: regulation by tumor necrosis factor alpha. *Mol. Cell Biol.* **17**, 5719–5726 (1997). 9315630 10.1128/mcb.17.10.5719 PMC232420

[235] Fleming, I., Fisslthaler, B., Dimmeler, S., Kemp, B. E. & Busse, R. Phosphorylation of Thr(495) regulates Ca(2+)/calmodulin-dependent endothelial nitric oxide synthase activity. *Circ. Res.* **88**, E68–E75 (2001). 11397791 10.1161/hh1101.092677

[236] Neumann, P., Gertzberg, N. & Johnson, A. TNF-alpha induces a decrease in eNOS promoter activity. *Am. J. Physiol. Lung Cell Mol. Physiol.* **286**, L452–L459 (2004). 14555463 10.1152/ajplung.00378.2002

[237] Jain, S. et al. Upregulation of human angiotensinogen (AGT) gene transcription by interferon-gamma: involvement of the STAT1-binding motif in the AGT promoter. *Biochim. Biophys. Acta* **1759**, 340–347 (2006). 16949687 10.1016/j.bbaexp.2006.07.003

[238] McMaster, W. G., Kirabo, A., Madhur, M. S. & Harrison, D. G. Inflammation, immunity, and hypertensive end-organ damage. *Circ. Res.* **116**, 1022–1033 (2015). 25767287 10.1161/CIRCRESAHA.116.303697 PMC4535695

[239] Koskela, S. et al. Coagulopathy in acute Puumala hantavirus infection. *Viruses* **13**, 1553 (2021). 34452419 10.3390/v13081553 PMC8402851

[240] Ronco, C. & Reis, T. Kidney involvement in COVID-19 and rationale for extracorporeal therapies. *Nat. Rev. Nephrol.* **16**, 308–310 (2020). 32273593 10.1038/s41581-020-0284-7 PMC7144544

[241] Shibabaw, T., Molla, M. D., Teferi, B. & Ayelign, B. Role of IFN and complements system: innate immunity in SARS-CoV-2. *J. Inflamm. Res.* **13**, 507–518 (2020). 32982366 10.2147/JIR.S267280 PMC7490109

[242] Petr, V. & Thurman, J. M. The role of complement in kidney disease. *Nat. Rev. Nephrol.* **19**, 771–787 (2023). 37735215 10.1038/s41581-023-00766-1

[243] Heinrich, P. C., Castell, J. V. & Andus, T. Interleukin-6 and the acute phase response. *Biochem. J.* **265**, 621–636 (1990). 1689567 10.1042/bj2650621 PMC1133681

[244] Okuda, Y. A. A amyloidosis—benefits and prospects of IL-6 inhibitors. *Mod. Rheumatol.* **29**, 268–274 (2019). 30132351 10.1080/14397595.2018.1515145

[245] Schmidt-Arras, D. & Rose-John, S. IL-6 pathway in the liver: from physiopathology to therapy. *J. Hepatol.* **64**, 1403–1415 (2016). 26867490 10.1016/j.jhep.2016.02.004

[246] Terpos, E. et al. Hematological findings and complications of COVID-19. *Am. J. Hematol.* **95**, 834–847 (2020). 32282949 10.1002/ajh.25829 PMC7262337

[247] Merad, M. & Martin, J. C. Pathological inflammation in patients with COVID-19: a key role for monocytes and macrophages. *Nat. Rev. Immunol.* **20**, 355–362 (2020). 32376901 10.1038/s41577-020-0331-4 PMC7201395

[248] Szabo, G. & Petrasek, J. Inflammasome activation and function in liver disease. *Nat. Rev. Gastroenterol. Hepatol.* **12**, 387–400 (2015). 26055245 10.1038/nrgastro.2015.94

[249] Sun, R. et al. STAT1 contributes to dsRNA inhibition of liver regeneration after partial hepatectomy in mice. *Hepatology* **44**, 955–966 (2006). 17006930 10.1002/hep.21344

[250] Schwabe, R. F. & Brenner, D. A. Mechanisms of liver injury. I. TNF-alpha-induced liver injury: role of IKK, JNK, and ROS pathways. *Am. J. Physiol. Gastrointest. Liver Physiol.* **290**, G583–G589 (2006). 16537970 10.1152/ajpgi.00422.2005

[251] Gust, J., Ponce, R., Liles, W. C., Garden, G. A. & Turtle, C. J. Cytokines in CAR T cell-associated neurotoxicity. *Front. Immunol.* **11**, 577027 (2020). 33391257 10.3389/fimmu.2020.577027 PMC7772425

[252] Iacobone, E. et al. Sepsis-associated encephalopathy and its differential diagnosis. *Crit. Care Med.* **37**, S331–S336 (2009). 20046118 10.1097/CCM.0b013e3181b6ed58

[253] Ichiyama, T. et al. Cerebrospinal fluid and serum levels of cytokines and soluble tumor necrosis factor receptor in influenza virus-associated encephalopathy. *Scand. J. Infect. Dis.* **35**, 59–61 (2003). 12685886 10.1080/0036554021000026986

[254] Mahdi, J. et al. Tumor inflammation-associated neurotoxicity. *Nat. Med.* **29**, 803–810 (2023). 37024595 10.1038/s41591-023-02276-w PMC10166099

[255] Ichiyama, T. et al. Analysis of cytokine levels and NF-kappaB activation in peripheral blood mononuclear cells in influenza virus-associated encephalopathy. *Cytokine* **27**, 31–37 (2004). 15207249 10.1016/j.cyto.2004.03.012

[256] Winter, P. M. et al. Proinflammatory cytokines and chemokines in humans with Japanese encephalitis. *J. Infect. Dis.* **190**, 1618–1626 (2004). 15478067 10.1086/423328

[257] Brudno, J. N. & Kochenderfer, J. N. Recent advances in CAR T-cell toxicity: mechanisms, manifestations and management. *Blood Rev.* **34**, 45–55 (2019). 30528964 10.1016/j.blre.2018.11.002 PMC6628697

[258] Srikiatkhachorn, A., Mathew, A. & Rothman, A. L. Immune-mediated cytokine storm and its role in severe dengue. *Semin Immunopathol.* **39**, 563–574 (2017). 28401256 10.1007/s00281-017-0625-1 PMC5496927

[259] Gust, J. et al. Endothelial activation and blood-brain barrier disruption in neurotoxicity after adoptive immunotherapy with CD19 CAR-T cells. *Cancer Discov.* **7**, 1404–1419 (2017). 29025771 10.1158/2159-8290.CD-17-0698 PMC5718945

[260] Rochfort, K. D. & Cummins, P. M. The blood-brain barrier endothelium: a target for pro-inflammatory cytokines. *Biochem. Soc. Trans.* **43**, 702–706 (2015). 26551716 10.1042/BST20140319

[261] Rothaug, M., Becker-Pauly, C. & Rose-John, S. The role of interleukin-6 signaling in nervous tissue. *Biochim. Biophys. Acta* **1863**, 1218–1227 (2016). 27016501 10.1016/j.bbamcr.2016.03.018

[262] Kettenmann, H., Hanisch, U. K., Noda, M. & Verkhratsky, A. Physiology of microglia. *Physiol. Rev.* **91**, 461–553 (2011). 21527731 10.1152/physrev.00011.2010

[263] Argaw, A. T., Gurfein, B. T., Zhang, Y., Zameer, A. & John, G. R. VEGF-mediated disruption of endothelial CLN-5 promotes blood-brain barrier breakdown. *Proc. Natl Acad. Sci. USA* **106**, 1977–1982 (2009). 19174516 10.1073/pnas.0808698106 PMC2644149

[264] Karschnia, P. et al. Neurologic toxicities following adoptive immunotherapy with BCMA-directed CAR T cells. *Blood* **142**, 1243–1248 (2023). 37471607 10.1182/blood.2023020571

[265] Smith, D. E. et al. A central nervous system-restricted isoform of the interleukin-1 receptor accessory protein modulates neuronal responses to interleukin-1. *Immunity* **30**, 817–831 (2009). 19481478 10.1016/j.immuni.2009.03.020 PMC4103746

[266] Lin, F. Y. et al. Phase I trial of GD2.CART cells augmented with constitutive interleukin-7 receptor for treatment of high-grade pediatric CNS tumors. *J. Clin. Oncol.* **42**, 2769–2779 (2024). 38771986 10.1200/JCO.23.02019 PMC11305939

[267] Sharma, A. N., Stultz, J. R., Bellamkonda, N. & Amsterdam, E. A. Fulminant myocarditis: epidemiology, pathogenesis, diagnosis, and management. *Am. J. Cardiol.* **124**, 1954–1960 (2019). 31679645 10.1016/j.amjcard.2019.09.017

[268] Ammirati, E. et al. Update on acute myocarditis. *Trends Cardiovasc. Med.* **31**, 370–379 (2021). 32497572 10.1016/j.tcm.2020.05.008 PMC7263216

[269] Barhoum, P. et al. Phenotypic heterogeneity of fulminant COVID-19-related myocarditis in adults. *J. Am. Coll. Cardiol.* **80**, 299–312 (2022). 35863846 10.1016/j.jacc.2022.04.056 PMC9291241

[270] Jiang, J. et al. Chinese Society of Cardiology guidelines on the diagnosis and treatment of adult fulminant myocarditis. *Sci. China Life Sci.* **67**, 913–939 (2024). 38332216 10.1007/s11427-023-2421-0

[271] Okuni, M., Yamada, T., Mochizuki, S. & Sakurai, I. Studies on myocarditis in childhood, with special reference to the possible role of immunological process and the thymus in the chronicity of the disease. *Jpn Circ. J.* **39**, 463–470 (1975). 47406 10.1253/jcj.39.463

[272] Kociol, R. D. et al. Recognition and initial management of fulminant myocarditis: a scientific statement from the American Heart Association. *Circulation* **141**, e69–e92 (2020). 31902242 10.1161/CIR.0000000000000745

[273] McCarthy, R. E. 3rd et al. Long-term outcome of fulminant myocarditis as compared with acute (nonfulminant) myocarditis. *N. Engl. J. Med.* **342**, 690–695 (2000). 10706898 10.1056/NEJM200003093421003

[274] Huang, F. et al. Fulminant myocarditis proven by early biopsy and outcomes. *Eur. Heart J.* **44**, 5110–5124 (2023). 37941449 10.1093/eurheartj/ehad707

[275] Gupta, S., Markham, D. W., Drazner, M. H. & Mammen, P. P. Fulminant myocarditis. *Nat. Clin. Pr. Cardiovasc. Med.* **5**, 693–706 (2008). 10.1038/ncpcardio1331 18797433

[276] Abdelnabi, M., Eshak, N., Saleh, Y. & Almaghraby, A. Coronavirus disease 2019 myocarditis: insights into pathophysiology and management. *Eur. Cardiol.* **15**, e51 (2020). 32617120 10.15420/ecr.2020.16 PMC7325211

[277] Akhmerov, A. & Marbán, E. COVID-19 and the heart. *Circ. Res.* **126**, 1443–1455 (2020). 32252591 10.1161/CIRCRESAHA.120.317055

[278] Herrmann, J. Adverse cardiac effects of cancer therapies: cardiotoxicity and arrhythmia. *Nat. Rev. Cardiol.* **17**, 474–502 (2020). 32231332 10.1038/s41569-020-0348-1 PMC8782611

[279] Knowlton, K. U. & Yajima, T. Interleukin-10: biomarker or pathologic cytokine in fulminant myocarditis? *J. Am. Coll. Cardiol.* **44**, 1298–1300 (2004). 15364335 10.1016/j.jacc.2004.06.026

[280] Nishii, M. et al. Serum levels of interleukin-10 on admission as a prognostic predictor of human fulminant myocarditis. *J. Am. Coll. Cardiol.* **44**, 1292–1297 (2004). 15364334 10.1016/j.jacc.2004.01.055

[281] Liu, W. et al. Thioredoxin-1 ameliorates myosin-induced autoimmune myocarditis by suppressing chemokine expressions and leukocyte chemotaxis in mice. *Circulation* **110**, 1276–1283 (2004). 15337697 10.1161/01.CIR.0000141803.41217.B6

[282] Ma, P. et al. Expansion of pathogenic cardiac macrophages in immune checkpoint inhibitor myocarditis. *Circulation* **149**, 48–66 (2024). 37746718 10.1161/CIRCULATIONAHA.122.062551 PMC11323830

[283] Kanaoka, K. et al. Features and outcomes of histologically proven myocarditis with fulminant presentation. *Circulation* **146**, 1425–1433 (2022). 36164974 10.1161/CIRCULATIONAHA.121.058869

[284] Siripanthong, B. et al. Recognizing COVID-19-related myocarditis: the possible pathophysiology and proposed guideline for diagnosis and management. *Heart Rhythm* **17**, 1463–1471 (2020). 32387246 10.1016/j.hrthm.2020.05.001 PMC7199677

[285] Moslehi, J., Lichtman, A. H., Sharpe, A. H., Galluzzi, L. & Kitsis, R. N. Immune checkpoint inhibitor-associated myocarditis: manifestations and mechanisms. *J. Clin. Investig.* **131**, e145186 (2021). 33645548 10.1172/JCI145186 PMC7919710

[286] Shioji, K., Kishimoto, C. & Sasayama, S. Fc receptor-mediated inhibitory effect of immunoglobulin therapy on autoimmune giant cell myocarditis: concomitant suppression of the expression of dendritic cells. *Circ. Res.* **89**, 540–546 (2001). 11557742 10.1161/hh1801.096263

[287] Wei, X., Fang, Y. & Hu, H. Glucocorticoid and immunoglobulin to treat viral fulminant myocarditis. *Eur. Heart J.* **41**, 2122 (2020). 32338741 10.1093/eurheartj/ehaa357 PMC7279514

[288] Hu, H., Ma, F., Wei, X. & Fang, Y. Coronavirus fulminant myocarditis treated with glucocorticoid and human immunoglobulin. *Eur. Heart J.* **42**, 206 (2021). 32176300 10.1093/eurheartj/ehaa190 PMC7184348

[289] Ammirati, E. et al. Fulminant versus acute nonfulminant myocarditis in patients with left ventricular systolic dysfunction. *J. Am. Coll. Cardiol.* **74**, 299–311 (2019). 31319912 10.1016/j.jacc.2019.04.063

[290] Yamamoto, M., Tajiri, K., Ayuzawa, S. & Ieda, M. Pathological findings of clinically suspected myocarditis temporally associated with COVID-19 vaccination. *Eur. J. Heart Fail* **24**, 1132–1138 (2022). 35488842 10.1002/ejhf.2523 PMC9348161

[291] Pollack, A., Kontorovich, A. R., Fuster, V. & Dec, G. W. Viral myocarditis–diagnosis, treatment options, and current controversies. *Nat. Rev. Cardiol.* **12**, 670–680 (2015). 26194549 10.1038/nrcardio.2015.108

[292] Caforio, A. L. et al. Current state of knowledge on aetiology, diagnosis, management, and therapy of myocarditis: a position statement of the European Society of Cardiology Working Group on Myocardial and Pericardial Diseases. *Eur. Heart J.* **34**, 2636–2648 (2013). 23824828 10.1093/eurheartj/eht210

[293] Maisch, B., Ruppert, V. & Pankuweit, S. Management of fulminant myocarditis: a diagnosis in search of its etiology but with therapeutic options. *Curr. Heart Fail Rep.* **11**, 166–177 (2014). 24723087 10.1007/s11897-014-0196-6

[294] Zheng, S. Y. & Dong, J. Z. Role of toll-like receptors and th responses in viral myocarditis. *Front. Immunol.* **13**, 843891 (2022). 35514979 10.3389/fimmu.2022.843891 PMC9062100

[295] Pannucci, P. et al. COVID-19-induced myocarditis: pathophysiological roles of ACE2 and toll-like receptors. *Int. J. Mol. Sci.* **24**, 5374 (2023). 36982447 10.3390/ijms24065374 PMC10049267

[296] Oka, T. et al. Mitochondrial DNA that escapes from autophagy causes inflammation and heart failure. *Nature* **485**, 251–255 (2012). 22535248 10.1038/nature10992 PMC3378041

[297] Jenke, A. et al. Adiponectin protects against Toll-like receptor 4-mediated cardiac inflammation and injury. *Cardiovasc. Res.* **99**, 422–431 (2013). 23674516 10.1093/cvr/cvt118

[298] Lafferty, E. I., Wiltshire, S. A., Angers, I., Vidal, S. M. & Qureshi, S. T. Unc93b1 -dependent endosomal toll-like receptor signaling regulates inflammation and mortality during coxsackievirus B3 infection. *J. Innate Immun.* **7**, 315–330 (2015). 25675947 10.1159/000369342 PMC6738833

[299] Li, B. et al. Interleukin-37 alleviates myocardial injury induced by coxsackievirus B3 via inhibiting neutrophil extracellular traps formation. *Int. Immunopharmacol.* **113**, 109343 (2022). 36308891 10.1016/j.intimp.2022.109343

[300] Chen, Y. F. et al. Investigate the role of neutrophil extracellular traps in immune checkpoint inhibitor-associated myocarditis with programmed death protein-1 inhibitors involvement. *Zhonghua Yi Xue Za Zhi* **103**, 3384–3393 (2023). 37963736 10.3760/cma.j.cn112137-20230901-00357

[301] Yu, Y. et al. Inhibition of calpain alleviates coxsackievirus B3-induced myocarditis through suppressing the canonical NLRP3 inflammasome/caspase-1-mediated and noncanonical caspase-11-mediated pyroptosis pathways. *Am. J. Transl. Res.* **12**, 1954–1964 (2020). 32509190 PMC7270028

[302] Xu, J. et al. Roles of inflammasomes in viral myocarditis. *Front. Cell. Infect. Microbiol.* **13**, 1149911 (2023). 37256114 10.3389/fcimb.2023.1149911 PMC10225676

[303] Xu, D. et al. Gr-1+ cells other than Ly6G+ neutrophils limit virus replication and promote myocardial inflammation and fibrosis following coxsackievirus B3 infection of mice. *Front. Cell. Infect. Microbiol.* **8**, 157 (2018). 29868513 10.3389/fcimb.2018.00157 PMC5962688

[304] Rivadeneyra, L. et al. Role of neutrophils in CVB3 infection and viral myocarditis. *J. Mol. Cell Cardiol.* **125**, 149–161 (2018). 30393107 10.1016/j.yjmcc.2018.08.029

[305] Bracamonte-Baran, W. & Čiháková, D. Cardiac autoimmunity: myocarditis. *Adv. Exp. Med. Biol.* **1003**, 187–221 (2017). 28667560 10.1007/978-3-319-57613-8_10 PMC5706653

[306] Barin, J. G. & Cihakova, D. Control of inflammatory heart disease by CD4+ T cells. *Ann. N. Y. Acad. Sci.* **1285**, 80–96 (2013). 23692566 10.1111/nyas.12134

[307] Kishimoto, C., Kawamata, H., Sakai, S., Shinohara, H. & Ochiai, H. Role of MIP-2 in coxsackievirus B3 myocarditis. *J. Mol. Cell Cardiol.* **32**, 631–638 (2000). 10756119 10.1006/jmcc.2000.1102

[308] Göser, S. et al. Critical role for monocyte chemoattractant protein-1 and macrophage inflammatory protein-1alpha in induction of experimental autoimmune myocarditis and effective anti-monocyte chemoattractant protein-1 gene therapy. *Circulation* **112**, 3400–3407 (2005). 16316965 10.1161/CIRCULATIONAHA.105.572396

[309] Zhang, Y., Zhou, X., Chen, S., Sun, X. & Zhou, C. Immune mechanisms of group B coxsackievirus induced viral myocarditis. *Virulence* **14**, 2180951 (2023). 36827455 10.1080/21505594.2023.2180951 PMC9980623

[310] Imanaka-Yoshida, K. Inflammation in myocardial disease: From myocarditis to dilated cardiomyopathy. *Pathol. Int.* **70**, 1–11 (2020). 31691489 10.1111/pin.12868

[311] Lee, J. Y., Lee, S. H. & Kim, W. H. Fulminant eosinophilic myocarditis without peripheral eosinophilia. *Tex. Heart Inst. J.* **50**, e217818 (2023). 37044058 10.14503/THIJ-21-7818 PMC10178658

[312] Brambatti, M. et al. Eosinophilic myocarditis: characteristics, treatment, and outcomes. *J. Am. Coll. Cardiol.* **70**, 2363–2375 (2017). 29096807 10.1016/j.jacc.2017.09.023

[313] Barin, J. G. et al. Fatal eosinophilic myocarditis develops in the absence of IFN-γ and IL-17A. *J. Immunol.* **191**, 4038–4047 (2013). 24048893 10.4049/jimmunol.1301282 PMC3927983

[314] Zhu, H. et al. Identification of pathogenic immune cell subsets associated with checkpoint inhibitor-induced myocarditis. *Circulation* **146**, 316–335 (2022). 35762356 10.1161/CIRCULATIONAHA.121.056730 PMC9397491

[315] Tarrio, M. L., Grabie, N., Bu, D. X., Sharpe, A. H. & Lichtman, A. H. PD-1 protects against inflammation and myocyte damage in T cell-mediated myocarditis. *J. Immunol.* **188**, 4876–4884 (2012). 22491251 10.4049/jimmunol.1200389 PMC3345066

[316] Wang, J. et al. PD-1 deficiency results in the development of fatal myocarditis in MRL mice. *Int. Immunol.* **22**, 443–452 (2010). 20410257 10.1093/intimm/dxq026

[317] Bockstahler, M. et al. Heart-specific immune responses in an animal model of autoimmune-related myocarditis mitigated by an immunoproteasome inhibitor and genetic ablation. *Circulation* **141**, 1885–1902 (2020). 32160764 10.1161/CIRCULATIONAHA.119.043171

[318] Grabie, N., Lichtman, A. H. & Padera, R. T cell checkpoint regulators in the heart. *Cardiovasc. Res.* **115**, 869–877 (2019). 30721928 10.1093/cvr/cvz025 PMC6452292

[319] Johnson, D. B. et al. Fulminant myocarditis with combination immune checkpoint blockade. *N. Engl. J. Med.* **375**, 1749–1755 (2016). 27806233 10.1056/NEJMoa1609214 PMC5247797

[320] Kusama, Y. et al. Variant angina and coronary artery spasm: the clinical spectrum, pathophysiology, and management. *J. Nippon Med. Sch.* **78**, 4–12 (2011). 21389642 10.1272/jnms.78.4

[321] Choi, B. G. et al. Five-year clinical outcomes in patients with significant coronary artery spasm: a propensity score-matched analysis. *Int J. Cardiol.* **184**, 533–539 (2015). 25767010 10.1016/j.ijcard.2015.02.021

[322] Kitulwatte, I., Gangahawatte, S., Perera, U. & Edirisinghe, P. Death following ceftazidime-induced Kounis syndrome. *Med. Leg. J.* **85**, 215–218 (2017). 29210337 10.1177/0025817217695904

[323] Xu, X. et al. Variant angina is associated with myocarditis. *J. Inflamm. Res.* **15**, 4939–4949 (2022). 36060213 10.2147/JIR.S378152 PMC9439647

[324] Li, S. et al. A life support-based comprehensive treatment regimen dramatically lowers the in-hospital mortality of patients with fulminant myocarditis: a multiple center study. *Sci. China Life Sci.* **62**, 369–380 (2019). 30850929 10.1007/s11427-018-9501-9

[325] Kowalewski, M. et al. COVID-19 and ECMO: the interplay between coagulation and inflammation-a narrative review. *Crit. Care* **24**, 205 (2020). 32384917 10.1186/s13054-020-02925-3 PMC7209766

[326] Millar, J. E., Fanning, J. P., McDonald, C. I., McAuley, D. F. & Fraser, J. F. The inflammatory response to extracorporeal membrane oxygenation (ECMO): a review of the pathophysiology. *Crit. Care* **20**, 387 (2016). 27890016 10.1186/s13054-016-1570-4 PMC5125043

[327] Tschope, C. et al. Mechanical unloading by fulminant myocarditis: LV-IMPELLA, ECMELLA, BI-PELLA, and PROPELLA concepts. *J. Cardiovasc. Transl. Res.* **12**, 116–123 (2019). 30084076 10.1007/s12265-018-9820-2 PMC6497621

[328] Auger, J. P. et al. Metabolic rewiring promotes anti-inflammatory effects of glucocorticoids. *Nature* **629**, 184–192 (2024). 38600378 10.1038/s41586-024-07282-7

[329] Cain, D. W. & Cidlowski, J. A. Immune regulation by glucocorticoids. *Nat. Rev. Immunol.* **17**, 233–247 (2017). 28192415 10.1038/nri.2017.1 PMC9761406

[330] Zhang, Z. et al. Itaconate is a lysosomal inducer that promotes antibacterial innate immunity. *Mol. Cell* **82**, 2844–2857.e2810 (2022). 35662396 10.1016/j.molcel.2022.05.009

[331] Olagnier, D. et al. SARS-CoV2-mediated suppression of NRF2-signaling reveals potent antiviral and anti-inflammatory activity of 4-octyl-itaconate and dimethyl fumarate. *Nat. Commun.* **11**, 4938 (2020). 33009401 10.1038/s41467-020-18764-3 PMC7532469

[332] Nakamura, H., Kunitsugu, I., Fukuda, K., Matsuzaki, M. & Sano, M. Diverse stage-dependent effects of glucocorticoids in a murine model of viral myocarditis. *J. Cardiol.* **61**, 237–242 (2013). 23415923 10.1016/j.jjcc.2012.11.006

[333] Blagova, O., Nedostup, A., Kogan, E., Zaitsev, A. & Fomin, V. Immunosuppressive therapy of biopsy proved immune-mediated lymphocytic myocarditis in the virus-negative and virus-positive patients. *Cardiovasc. Pathol.* **49**, 107260 (2020). 32683240 10.1016/j.carpath.2020.107260

[334] Shi, Z., He, Z. & Wang, D. W. CYP450 epoxygenase metabolites, epoxyeicosatrienoic acids, as novel anti-inflammatory mediators. *Molecules* **27**, 3873 (2022). 35744996 10.3390/molecules27123873 PMC9230517

[335] Zhou, Z. et al. Epoxyeicosatrienoic acids prevent cardiac dysfunction in viral myocarditis via interferon type I signaling. *Circ. Res.* **133**, 772–788 (2023). 37681352 10.1161/CIRCRESAHA.123.322619

[336] Drucker, N. A. et al. Gamma-globulin treatment of acute myocarditis in the pediatric population. *Circulation* **89**, 252–257 (1994). 8281654 10.1161/01.cir.89.1.252

[337] Goland, S. et al. Intravenous immunoglobulin treatment for acute fulminant inflammatory cardiomyopathy: series of six patients and review of literature. *Can. J. Cardiol.* **24**, 571–574 (2008). 18612500 10.1016/s0828-282x(08)70638-x PMC2640335

[338] Kishimoto, C. et al. Therapy with immunoglobulin in patients with acute myocarditis and cardiomyopathy: analysis of leukocyte balance. *Heart Vessels* **29**, 336–342 (2014). 23702697 10.1007/s00380-013-0368-4

[339] Mason, J. W. et al. A clinical trial of immunosuppressive therapy for myocarditis. The myocarditis treatment trial investigators. *N. Engl. J. Med.* **333**, 269–275 (1995). 7596370 10.1056/NEJM199508033330501

[340] Zhang, L. et al. Functional metabolomics characterizes a key role for N-acetylneuraminic acid in coronary artery diseases. *Circulation* **137**, 1374–1390 (2018). 29212895 10.1161/CIRCULATIONAHA.117.031139

[341] Antonucci, E. et al. Incidence of acute kidney injury and attributive mortality in acute respiratory distress syndrome randomized trials. *Intensive Care Med.* **50**, 1240–1250 (2024). 38864911 10.1007/s00134-024-07485-6 PMC11306535

[342] Radermacher, P., Maggiore, S. M. & Mercat, A. Fifty years of research in ARDS. Gas exchange in acute respiratory distress syndrome. *Am. J. Respir. Crit. Care Med.* **196**, 964–984 (2017). 28406724 10.1164/rccm.201610-2156SO

[343] Moore, J. B. & June, C. H. Cytokine release syndrome in severe COVID-19. *Science* **368**, 473–474 (2020). 32303591 10.1126/science.abb8925

[344] Ackermann, M. et al. Pulmonary vascular endothelialitis, thrombosis, and angiogenesis in Covid-19. *N. Engl. J. Med.* **383**, 120–128 (2020). 32437596 10.1056/NEJMoa2015432 PMC7412750

[345] Delorey, T. M. et al. COVID-19 tissue atlases reveal SARS-CoV-2 pathology and cellular targets. *Nature* **595**, 107–113 (2021). 33915569 10.1038/s41586-021-03570-8 PMC8919505

[346] Brun-Buisson, C., Richard, J. C., Mercat, A., Thiébaut, A. C. & Brochard, L. Early corticosteroids in severe influenza A/H1N1 pneumonia and acute respiratory distress syndrome. *Am. J. Respir. Crit. Care Med.* **183**, 1200–1206 (2011). 21471082 10.1164/rccm.201101-0135OC

[347] Short, K. R., Kroeze, E., Fouchier, R. A. M. & Kuiken, T. Pathogenesis of influenza-induced acute respiratory distress syndrome. *Lancet Infect. Dis.* **14**, 57–69 (2014). 24239327 10.1016/S1473-3099(13)70286-X

[348] Chan, L. L. Y. et al. Host DNA released by NETosis in neutrophils exposed to seasonal H1N1 and highly pathogenic H5N1 influenza viruses. *Respir. Res.* **21**, 160 (2020). 32576265 10.1186/s12931-020-01425-w PMC7310290

[349] Kim, J., Hickerson, B. T. & Ilyushina, N. A. Coinfection of influenza A and B and human OC43 coronavirus in normal human bronchial epithelial cells. *Influenza Other Respir. Viruses* **18**, e13279 (2024). 38556468 10.1111/irv.13279 PMC10982074

[350] Huang, W. et al. Protection effects of mice liver and lung injury induced by coronavirus infection of Qingfei Paidu decoction involve inhibition of the NLRP3 signaling pathway. *J. Ethnopharmacol.* **321**, 117512 (2024). 38040130 10.1016/j.jep.2023.117512

[351] Lau, K. Y. et al. Estimating the epidemic size of superspreading coronavirus outbreaks in real time: quantitative study. *JMIR Public Health Surveill.* **10**, e46687 (2024). 38345850 10.2196/46687 PMC10863650

[352] Fu, S. et al. Targeted amplicon sequencing facilitated a novel risk assessment framework for assessing the prevalence of broad spectrum bacterial and coronavirus diseases. *Sci. Total Environ.* **912**, 168797 (2024). 38007133 10.1016/j.scitotenv.2023.168797

[353] Catanzaro, M. et al. Immune response in COVID-19: addressing a pharmacological challenge by targeting pathways triggered by SARS-CoV-2. *Signal Transduct. Target. Ther.* **5**, 84 (2020). 32467561 10.1038/s41392-020-0191-1 PMC7255975

[354] Mahallawi, W. H., Khabour, O. F., Zhang, Q., Makhdoum, H. M. & Suliman, B. A. MERS-CoV infection in humans is associated with a pro-inflammatory Th1 and Th17 cytokine profile. *Cytokine* **104**, 8–13 (2018). 29414327 10.1016/j.cyto.2018.01.025 PMC7129230

[355] Wong, C. K. et al. Plasma inflammatory cytokines and chemokines in severe acute respiratory syndrome. *Clin. Exp. Immunol.* **136**, 95–103 (2004). 15030519 10.1111/j.1365-2249.2004.02415.x PMC1808997

[356] Tian, M. et al. HIF-1alpha promotes SARS-CoV-2 infection and aggravates inflammatory responses to COVID-19. *Signal Transduct. Target. Ther.* **6**, 308 (2021). 34408131 10.1038/s41392-021-00726-w PMC8371950

[357] Luo, W. et al. Targeting JAK-STAT signaling to control cytokine release syndrome in COVID-19. *Trends Pharm. Sci.* **41**, 531–543 (2020). 32580895 10.1016/j.tips.2020.06.007 PMC7298494

[358] Baek, Y. B. et al. Therapeutic strategy targeting host lipolysis limits infection by SARS-CoV-2 and influenza A virus. *Signal Transduct. Target. Ther.* **7**, 367 (2022). 36253361 10.1038/s41392-022-01223-4 PMC9575645

[359] Liu, Q., Liu, D. Y. & Yang, Z. Q. Characteristics of human infection with avian influenza viruses and development of new antiviral agents. *Acta Pharm. Sin.* **34**, 1257–1269 (2013). 10.1038/aps.2013.121 PMC3791557 24096642

[360] Oshansky, C. M. et al. Mucosal immune responses predict clinical outcomes during influenza infection independently of age and viral load. *Am. J. Respir. Crit. Care Med.* **189**, 449–462 (2014). 24308446 10.1164/rccm.201309-1616OC PMC3977720

[361] Coates, B. M. et al. Inhibition of the NOD-like receptor protein 3 inflammasome is protective in juvenile influenza A virus infection. *Front. Immunol.* **8**, 782 (2017). 28740490 10.3389/fimmu.2017.00782 PMC5502347

[362] Guo, X. J. & Thomas, P. G. New fronts emerge in the influenza cytokine storm. *Semin. Immunopathol.* **39**, 541–550 (2017). 28555383 10.1007/s00281-017-0636-y PMC5580809

[363] Liu, Q., Zhou, Y. H. & Yang, Z. Q. The cytokine storm of severe influenza and development of immunomodulatory therapy. *Cell Mol. Immunol.* **13**, 3–10 (2016). 26189369 10.1038/cmi.2015.74 PMC4711683

[364] Teijaro, J. R. The role of cytokine responses during influenza virus pathogenesis and potential therapeutic options. *Curr. Top. Microbiol. Immunol.* **386**, 3–22 (2015). 25267464 10.1007/82_2014_411

[365] Taubenberger, J. K. & Morens, D. M. The pathology of influenza virus infections. *Annu Rev. Pathol.* **3**, 499–522 (2008). 18039138 10.1146/annurev.pathmechdis.3.121806.154316 PMC2504709

[366] Le, V. B. et al. Platelet activation and aggregation promote lung inflammation and influenza virus pathogenesis. *Am. J. Respir. Crit. Care Med.* **191**, 804–819 (2015). 25664391 10.1164/rccm.201406-1031OC

[367] Pujhari, S., Paul, S., Ahluwalia, J. & Rasgon, J. L. Clotting disorder in severe acute respiratory syndrome coronavirus 2. *Rev. Med. Virol.* **31**, e2177 (2021). 33022790 10.1002/rmv.2177 PMC7646030

[368] Kumar, V. Toll-like receptors in sepsis-associated cytokine storm and their endogenous negative regulators as future immunomodulatory targets. *Int. Immunopharmacol.* **89**, 107087 (2020). 33075714 10.1016/j.intimp.2020.107087 PMC7550173

[369] Rossman, J. S. & Lamb, R. A. Influenza virus assembly and budding. *Virology* **411**, 229–236 (2011). 21237476 10.1016/j.virol.2010.12.003 PMC3086653

[370] Iwasaki, A. & Pillai, P. S. Innate immunity to influenza virus infection. *Nat. Rev. Immunol.* **14**, 315–328 (2014). 24762827 10.1038/nri3665 PMC4104278

[371] Durbin, R. K., Kotenko, S. V. & Durbin, J. E. Interferon induction and function at the mucosal surface. *Immunol. Rev.* **255**, 25–39 (2013). 23947345 10.1111/imr.12101 PMC5972370

[372] Pothlichet, J. et al. Type I IFN triggers RIG-I/TLR3/NLRP3-dependent inflammasome activation in influenza A virus infected cells. *PLoS Pathog.* **9**, e1003256 (2013). 23592984 10.1371/journal.ppat.1003256 PMC3623797

[373] Ichinohe, T., Yamazaki, T., Koshiba, T. & Yanagi, Y. Mitochondrial protein mitofusin 2 is required for NLRP3 inflammasome activation after RNA virus infection. *Proc. Natl Acad. Sci. USA* **110**, 17963–17968 (2013). 24127597 10.1073/pnas.1312571110 PMC3816452

[374] Herold, S., Becker, C., Ridge, K. M. & Budinger, G. R. Influenza virus-induced lung injury: pathogenesis and implications for treatment. *Eur. Respir. J.* **45**, 1463–1478 (2015). 25792631 10.1183/09031936.00186214

[375] Davidson, S., Crotta, S., McCabe, T. M. & Wack, A. Pathogenic potential of interferon alphabeta in acute influenza infection. *Nat. Commun.* **5**, 3864 (2014). 24844667 10.1038/ncomms4864 PMC4033792

[376] Tate, M. D. et al. Reassessing the role of the NLRP3 inflammasome during pathogenic influenza A virus infection via temporal inhibition. *Sci. Rep.* **6**, 27912 (2016). 27283237 10.1038/srep27912 PMC4901306

[377] Ren, R. et al. The H7N9 influenza A virus infection results in lethal inflammation in the mammalian host via the NLRP3-caspase-1 inflammasome. *Sci. Rep.* **7**, 7625 (2017). 28790324 10.1038/s41598-017-07384-5 PMC5548739

[378] Hagau, N. et al. Clinical aspects and cytokine response in severe H1N1 influenza A virus infection. *Crit. Care* **14**, R203 (2010). 21062445 10.1186/cc9324 PMC3220006

[379] Wang, J. et al. Soluble interleukin-6 receptor is elevated during influenza A virus infection and mediates the IL-6 and IL-32 inflammatory cytokine burst. *Cell Mol. Immunol.* **12**, 633–644 (2015). 25176527 10.1038/cmi.2014.80 PMC4579649

[380] Cimini, E. & Agrati, C. γδ T cells in emerging viral infection: an overview. *Viruses* **14**, 1166 (2022). 35746638 10.3390/v14061166 PMC9230790

[381] Stout-Delgado, H. W., Du, W., Shirali, A. C., Booth, C. J. & Goldstein, D. R. Aging promotes neutrophil-induced mortality by augmenting IL-17 production during viral infection. *Cell Host Microbe* **6**, 446–456 (2009). 19917499 10.1016/j.chom.2009.09.011 PMC2779161

[382] Bermejo-Martin, J. F. et al. Th1 and Th17 hypercytokinemia as early host response signature in severe pandemic influenza. *Crit. Care* **13**, R201 (2009). 20003352 10.1186/cc8208 PMC2811892

[383] To, K. K. et al. Delayed clearance of viral load and marked cytokine activation in severe cases of pandemic H1N1 2009 influenza virus infection. *Clin. Infect. Dis.* **50**, 850–859 (2010). 20136415 10.1086/650581 PMC7107930

[384] Crowe, C. R. et al. Critical role of IL-17RA in immunopathology of influenza infection. *J. Immunol.* **183**, 5301–5310 (2009). 19783685 10.4049/jimmunol.0900995 PMC3638739

[385] Wang, D. et al. Clinical characteristics of 138 hospitalized patients with 2019 novel coronavirus-infected pneumonia in Wuhan, China. *JAMA* **323**, 1061–1069 (2020). 32031570 10.1001/jama.2020.1585 PMC7042881

[386] Yazar, S. et al. Single-cell eQTL mapping identifies cell type-specific genetic control of autoimmune disease. *Science* **376**, eabf3041 (2022). 35389779 10.1126/science.abf3041

[387] Aquino, Y. et al. Dissecting human population variation in single-cell responses to SARS-CoV-2. *Nature* **621**, 120–128 (2023). 37558883 10.1038/s41586-023-06422-9 PMC10482701

[388] Lakshmikanth, T. et al. Immune system adaptation during gender-affirming testosterone treatment. *Nature* **633**, 155–164 (2024). 39232147 10.1038/s41586-024-07789-z PMC11374716

[389] Russell, C. D., Millar, J. E. & Baillie, J. K. Clinical evidence does not support corticosteroid treatment for 2019-nCoV lung injury. *Lancet* **395**, 473–475 (2020). 32043983 10.1016/S0140-6736(20)30317-2 PMC7134694

[390] Engel, J. J. et al. Dexamethasone attenuates interferon-related cytokine hyperresponsiveness in COVID-19 patients. *Front. Immunol.* **14**, 1233318 (2023). 37614228 10.3389/fimmu.2023.1233318 PMC10442808

[391] Group, R. C. et al. Dexamethasone in hospitalized patients with Covid-19. *N. Engl. J. Med.* **384**, 693–704 (2021). 32678530 10.1056/NEJMoa2021436 PMC7383595

[392] Tomazini, B. M. et al. Effect of dexamethasone on days alive and ventilator-free in patients with moderate or severe acute respiratory distress syndrome and COVID-19: the CoDEX randomized clinical trial. *JAMA* **324**, 1307–1316 (2020). 32876695 10.1001/jama.2020.17021 PMC7489411

[393] Li, G., Hilgenfeld, R., Whitley, R. & De Clercq, E. Therapeutic strategies for COVID-19: progress and lessons learned. *Nat. Rev. Drug Discov.* **22**, 449–475 (2023). 37076602 10.1038/s41573-023-00672-y PMC10113999

[394] Luo, M. et al. IL-6 and CD8+ T cell counts combined are an early predictor of in-hospital mortality of patients with COVID-19. *JCI Insight* **5**, e139024 (2020). 32544099 10.1172/jci.insight.139024 PMC7406244

[395] Investigators, R.-C. et al. Interleukin-6 receptor antagonists in critically Ill patients with Covid-19. *N. Engl. J. Med.* **384**, 1491–1502 (2021). 33631065 10.1056/NEJMoa2100433 PMC7953461

[396] Soin, A. S. et al. Tocilizumab plus standard care versus standard care in patients in India with moderate to severe COVID-19-associated cytokine release syndrome (COVINTOC): an open-label, multicentre, randomised, controlled, phase 3 trial. *Lancet Respir. Med.* **9**, 511–521 (2021). 33676589 10.1016/S2213-2600(21)00081-3 PMC8078880

[397] Salama, C. et al. Tocilizumab in patients hospitalized with Covid-19 pneumonia. *N. Engl. J. Med.* **384**, 20–30 (2021). 33332779 10.1056/NEJMoa2030340 PMC7781101

[398] RECOVERY Collaborative Group. Tocilizumab in patients admitted to hospital with COVID-19 (RECOVERY): a randomised, controlled, open-label, platform trial. *Lancet* **397**, 1637–1645 (2021). 33933206 10.1016/S0140-6736(21)00676-0 PMC8084355

[399] McLornan, D. P., Pope, J. E., Gotlib, J. & Harrison, C. N. Current and future status of JAK inhibitors. *Lancet* **398**, 803–816 (2021). 34454676 10.1016/S0140-6736(21)00438-4

[400] Hung, I. F. et al. Convalescent plasma treatment reduced mortality in patients with severe pandemic influenza A (H1N1) 2009 virus infection. *Clin. Infect. Dis.* **52**, 447–456 (2011). 21248066 10.1093/cid/ciq106 PMC7531589

[401] RECOVERY Collaborative Group. Convalescent plasma in patients admitted to hospital with COVID-19 (RECOVERY): a randomised controlled, open-label, platform trial. *Lancet* **397**, 2049–2059 (2021). 34000257 10.1016/S0140-6736(21)00897-7 PMC8121538

[402] Bone, R. C., Sibbald, W. J. & Sprung, C. L. The ACCP-SCCM consensus conference on sepsis and organ failure. *Chest* **101**, 1481–1483 (1992). 1600757 10.1378/chest.101.6.1481

[403] Singer, M. et al. The third international consensus definitions for sepsis and septic shock (Sepsis-3). *JAMA* **315**, 801–810 (2016). 26903338 10.1001/jama.2016.0287 PMC4968574

[404] Xie, S. et al. Astragaloside IV attenuates sepsis-induced intestinal barrier dysfunction via suppressing RhoA/NLRP3 inflammasome signaling. *Int. Immunopharmacol.* **78**, 106066 (2020). 31835087 10.1016/j.intimp.2019.106066

[405] Chousterman, B. G., Swirski, F. K. & Weber, G. F. Cytokine storm and sepsis disease pathogenesis. *Semin. Immunopathol.* **39**, 517–528 (2017). 28555385 10.1007/s00281-017-0639-8

[406] Laudes, I. J. et al. Expression and function of C5a receptor in mouse microvascular endothelial cells. *J. Immunol.* **169**, 5962–5970 (2002). 12421982 10.4049/jimmunol.169.10.5962

[407] Huang, M., Cai, S. & Su, J. The pathogenesis of sepsis and potential therapeutic targets. *Int. J. Mol. Sci.* **20**, 5376 (2019). 31671729 10.3390/ijms20215376 PMC6862039

[408] Cheng, L. et al. Risk factors for systemic inflammatory response syndrome after percutaneous transhepatic cholangioscopic lithotripsy. *J. Inflamm. Res.* **17**, 2575–2587 (2024). 38686361 10.2147/JIR.S453653 PMC11057514

[409] Ashayeripanah, M. et al. Systemic inflammatory response syndrome triggered by blood-borne pathogens induces prolonged dendritic cell paralysis and immunosuppression. *Cell Rep.* **43**, 113754 (2024). 38354086 10.1016/j.celrep.2024.113754

[410] Rajam, G. et al. Development and validation of a sensitive and robust multiplex antigen capture assay to quantify streptococcus pneumoniae serotype-specific capsular polysaccharides in urine. *mSphere* **7**, e0011422 (2022). 35913133 10.1128/msphere.00114-22 PMC9429912

[411] Yang, X. et al. MBPD: a multiple bacterial pathogen detection pipeline for One Health practices. *Imeta* **2**, e82 (2023). 38868336 10.1002/imt2.82 PMC10989770

[412] Torrey, H. et al. A novel TNFR2 agonist antibody expands highly potent regulatory T cells. *Sci. Signal.* **13**, eaba9600 (2020). 33293464 10.1126/scisignal.aba9600

[413] Kargar, M., Torabizadeh, M., Purrahman, D., Zayeri, Z. D. & Saki, N. Regulatory factors involved in Th17/Treg cell balance of immune thrombocytopenia. *Curr. Res. Transl. Med.* **71**, 103389 (2023). 37062251 10.1016/j.retram.2023.103389

[414] Ma, R., Su, H., Jiao, K. & Liu, J. Role of Th17 cells, Treg cells, and Th17/Treg imbalance in immune homeostasis disorders in patients with chronic obstructive pulmonary disease. *Immun. Inflamm. Dis.* **11**, e784 (2023). 36840492 10.1002/iid3.784 PMC9950879

[415] Vignon, P., Laterre, P. F., Daix, T. & François, B. New agents in development for sepsis: any reason for hope? *Drugs* **80**, 1751–1761 (2020). 32951149 10.1007/s40265-020-01402-z PMC7502152

[416] Jin, Z., Suolitiken, D., Wang, Y. & Wang, Z. The diagnostic importance of multiple cytokines in adult hemophagocytic lymphohistiocytosis. *J. Clin. Lab. Anal.* **37**, e24669 (2023). 36036769 10.1002/jcla.24669 PMC10156101

[417] Yao, S. et al. Improved hemophagocytic lymphohistiocytosis index predicts prognosis of adult Epstein-Barr virus-associated HLH patients. *Ann. Med.* **55**, 89–100 (2023). 36533966 10.1080/07853890.2022.2149850 PMC9766494

[418] Zhang, J. et al. Genotype characteristics and immunological indicator evaluation of 311 hemophagocytic lymphohistiocytosis cases in China. *Orphanet J. Rare Dis.* **15**, 112 (2020). 32375849 10.1186/s13023-020-01390-z PMC7201972

[419] He, L. et al. Macrophage activation syndrome in adults: characteristics, outcomes, and therapeutic effectiveness of etoposide-based regimen. *Front. Immunol.* **13**, 955523 (2022). 36189240 10.3389/fimmu.2022.955523 PMC9520258

[420] Rosado, F. G. & Kim, A. S. Hemophagocytic lymphohistiocytosis: an update on diagnosis and pathogenesis. *Am. J. Clin. Pathol.* **139**, 713–727 (2013). 23690113 10.1309/AJCP4ZDKJ4ICOUAT

[421] Canna, S. W. & Marsh, R. A. Pediatric hemophagocytic lymphohistiocytosis. *Blood* **135**, 1332–1343 (2020). 32107531 10.1182/blood.2019000936 PMC8212354

[422] Al-Samkari, H. & Berliner, N. Hemophagocytic lymphohistiocytosis. *Annu. Rev. Pathol.* **13**, 27–49 (2018). 28934563 10.1146/annurev-pathol-020117-043625

[423] Jordan, M. B., Hildeman, D., Kappler, J. & Marrack, P. An animal model of hemophagocytic lymphohistiocytosis (HLH): CD8+ T cells and interferon gamma are essential for the disorder. *Blood* **104**, 735–743 (2004). 15069016 10.1182/blood-2003-10-3413

[424] Kögl, T. et al. Hemophagocytic lymphohistiocytosis in syntaxin-11-deficient mice: T-cell exhaustion limits fatal disease. *Blood* **121**, 604–613 (2013). 23190531 10.1182/blood-2012-07-441139

[425] Suolitiken, D., Wang, Y., Jin, Z. & Wang, Z. EBV protection- and susceptibility-related HLA alleles and EBV status in the Chinese population: a single-center study. *Immun. Inflamm. Dis.* **10**, e666 (2022). 35759244 10.1002/iid3.666 PMC9210551

[426] Zhang, K. et al. Hypomorphic mutations in PRF1, MUNC13-4, and STXBP2 are associated with adult-onset familial HLH. *Blood* **118**, 5794–5798 (2011). 21881043 10.1182/blood-2011-07-370148 PMC3228496

[427] Ishii, K. et al. Perforin-deficient CAR T cells recapitulate late-onset inflammatory toxicities observed in patients. *J. Clin. Investig.* **130**, 5425–5443 (2020). 32925169 10.1172/JCI130059 PMC7524496

[428] Ueda, I. et al. Correlation between phenotypic heterogeneity and gene mutational characteristics in familial hemophagocytic lymphohistiocytosis (FHL). *Pediatr. Blood Cancer* **46**, 482–488 (2006). 16365863 10.1002/pbc.20511

[429] Pagel, J. et al. Distinct mutations in STXBP2 are associated with variable clinical presentations in patients with familial hemophagocytic lymphohistiocytosis type 5 (FHL5). *Blood* **119**, 6016–6024 (2012). 22451424 10.1182/blood-2011-12-398958

[430] Zhang, K. et al. Synergistic defects of different molecules in the cytotoxic pathway lead to clinical familial hemophagocytic lymphohistiocytosis. *Blood* **124**, 1331–1334 (2014). 24916509 10.1182/blood-2014-05-573105 PMC4141517

[431] Ponnatt, T. S., Lilley, C. M. & Mirza, K. M. Hemophagocytic lymphohistiocytosis. *Arch. Pathol. Lab. Med.* **146**, 507–519 (2022). 34347856 10.5858/arpa.2020-0802-RA

[432] Yao, S. et al. Clinical features and prognostic risk prediction of non-Hodgkin lymphoma-associated hemophagocytic syndrome. *Front. Oncol.* **11**, 788056 (2021). 34938663 10.3389/fonc.2021.788056 PMC8685250

[433] Song, Y., Yin, Q., Wang, J. & Wang, Z. Autologous hematopoietic stem cell transplantation for patients with lymphoma-associated hemophagocytic lymphohistiocytosis. *Cell Transpl.* **30**, 9636897211057077 (2021). 10.1177/09636897211057077 PMC8579341 34743574

[434] Wang, J. et al. Chronic active Epstein-Barr virus disease originates from infected hematopoietic stem cells. *Blood* **143**, 32–41 (2024). 37824804 10.1182/blood.2023021074

[435] Ai, J. & Xie, Z. Epstein-Barr virus-positive T/NK-cell lymphoproliferative diseases in Chinese mainland. *Front. Pediatr.* **6**, 289 (2018). 30356785 10.3389/fped.2018.00289 PMC6189562

[436] Guan, Y. Q. et al. Inherited genetic susceptibility to nonimmunosuppressed Epstein-Barr virus-associated T/NK-cell lymphoproliferative diseases in Chinese patients. *Curr. Med. Sci.* **41**, 482–490 (2021). 34170459 10.1007/s11596-021-2375-5

[437] Kawada, J. I. et al. Updated guidelines for chronic active Epstein-Barr virus disease. *Int. J. Hematol.* **118**, 568–576 (2023). 37728704 10.1007/s12185-023-03660-5 PMC10615970

[438] Kimura, H. EBV in T-/NK-cell tumorigenesis. *Adv. Exp. Med. Biol.* **1045**, 459–475 (2018). 29896680 10.1007/978-981-10-7230-7_21

[439] Yao, S. et al. Epidemiological investigation of hemophagocytic lymphohistiocytosis in China. *Orphanet J. Rare Dis.* **16**, 342 (2021). 34344437 10.1186/s13023-021-01976-1 PMC8336372

[440] Ishii, E. Hemophagocytic lymphohistiocytosis in children: pathogenesis and treatment. *Front. Pediatr.* **4**, 47 (2016). 27242976 10.3389/fped.2016.00047 PMC4865497

[441] Khanna, R. & Gandhi, M. K. EBV-infected hematopoietic stem cells drive CAEBV. *Blood* **143**, 2–4 (2024). 38175680 10.1182/blood.2023022739

[442] Nikiforow, S. & Berliner, N. The unique aspects of presentation and diagnosis of hemophagocytic lymphohistiocytosis in adults. *Hematol. Am. Soc. Hematol. Educ. Program* **2015**, 183–189 (2015). 10.1182/asheducation-2015.1.183 26637719

[443] Nanno, S. et al. Diagnostic value of serum ferritin and the risk factors and cytokine profiles of hemophagocytic syndrome following allogeneic hematopoietic cell transplantation. *Leuk. Lymphoma* **58**, 1664–1672 (2017). 27919187 10.1080/10428194.2016.1262034

[444] Lichtenstein, D. A. et al. Characterization of HLH-like manifestations as a CRS variant in patients receiving CD22 CAR T cells. *Blood* **138**, 2469–2484 (2021). 34525183 10.1182/blood.2021011898 PMC8832442

[445] Sandler, R. D. et al. Diagnosis and management of secondary HLH/MAS following HSCT and CAR-T cell therapy in adults; a review of the literature and a survey of practice within EBMT centres on behalf of the Autoimmune Diseases Working Party (ADWP) and Transplant Complications Working Party (TCWP). *Front. Immunol.* **11**, 524 (2020). 32296434 10.3389/fimmu.2020.00524 PMC7137396

[446] Shakoory, B. et al. The 2022 EULAR/ACR points to consider at the early stages of diagnosis and management of suspected haemophagocytic lymphohistiocytosis/macrophage activation syndrome (HLH/MAS). *Ann. Rheum. Dis.* **82**, 1271–1285 (2023). 37487610 10.1136/ard-2023-224123 PMC11017727

[447] Xiao, L. et al. Low total cholesterol predicts early death in children with hemophagocytic lymphohistiocytosis. *Front. Pediatr.* **10**, 1006817 (2022). 36699307 10.3389/fped.2022.1006817 PMC9869152

[448] Pan, H. et al. Treatment outcomes and prognostic factors for non- malignancy associated secondary hemophagocytic lymphohistiocytosis in children. *BMC Pediatr.* **20**, 288 (2020). 32517812 10.1186/s12887-020-02178-7 PMC7281941

[449] Zoref-Lorenz, A. et al. An improved index for diagnosis and mortality prediction in malignancy-associated hemophagocytic lymphohistiocytosis. *Blood* **139**, 1098–1110 (2022). 34780598 10.1182/blood.2021012764 PMC8854682

[450] Feng, C. et al. A convenient and practical index for predicting the induction response in adult patients with hemophagocytic lymphohistiocytosis: ferritin/platelet ratio. *Ann. Hematol.* **103**, 715–723 (2024). 38197929 10.1007/s00277-023-05606-7 PMC10867095

[451] Cheng, W. et al. Prognostic value of the albumin-bilirubin score in patients with non-Hodgkin lymphoma-associated hemophagocytic lymphohistiocytosis. *Front. Immunol.* **14**, 1162320 (2023). 37266439 10.3389/fimmu.2023.1162320 PMC10229876

[452] Zhang, R. et al. A study on early death prognosis model in adult patients with secondary hemophagocytic lymphohistiocytosis. *J. Health. Eng.* **2022**, 6704859 (2022). 10.1155/2022/6704859 PMC9270127 35812895

[453] Cui, T., Wang, J. & Wang, Z. The outcome of induction therapy for EBV-related hemophagocytic lymphohistiocytosis: a model for risk stratification. *Front. Immunol.* **13**, 876415 (2022). 35860246 10.3389/fimmu.2022.876415 PMC9289144

[454] Xiao, L. et al. Predictive model for early death risk in pediatric hemophagocytic lymphohistiocytosis patients based on machine learning. *Heliyon* **9**, e22202 (2023). 38045172 10.1016/j.heliyon.2023.e22202 PMC10692822

[455] Bergsten, E. et al. Confirmed efficacy of etoposide and dexamethasone in HLH treatment: long-term results of the cooperative HLH-2004 study. *Blood* **130**, 2728–2738 (2017). 28935695 10.1182/blood-2017-06-788349 PMC5785801

[456] Hughes, A. D., Teachey, D. T. & Diorio, C. Riding the storm: managing cytokine-related toxicities in CAR-T cell therapy. *Semin. Immunopathol.* **46**, 5 (2024). 39012374 10.1007/s00281-024-01013-w PMC11252192

[457] Chen, L., Wang, J. & Wang, Z. L-DEP regimen is effective as an initial therapy for adult EBV-HLH. *Ann. Hematol.* **101**, 2461–2470 (2022). 36094533 10.1007/s00277-022-04946-0

[458] Wang, Y. et al. Multicenter study of combination DEP regimen as a salvage therapy for adult refractory hemophagocytic lymphohistiocytosis. *Blood* **126**, 2186–2192 (2015). 26289641 10.1182/blood-2015-05-644914 PMC4635114

[459] Wang, J. et al. PEG-aspargase and DEP regimen combination therapy for refractory Epstein-Barr virus-associated hemophagocytic lymphohistiocytosis. *J. Hematol. Oncol.* **9**, 84 (2016). 27613189 10.1186/s13045-016-0317-7 PMC5017041

[460] Al-Salama, Z. T. Emapalumab: first global approval. *Drugs* **79**, 99–103 (2019). 30623346 10.1007/s40265-018-1046-8

[461] Locatelli, F. et al. Emapalumab in children with primary hemophagocytic lymphohistiocytosis. *N. Engl. J. Med.* **382**, 1811–1822 (2020). 32374962 10.1056/NEJMoa1911326

[462] Rivat, C. et al. SAP gene transfer restores cellular and humoral immune function in a murine model of X-linked lymphoproliferative disease. *Blood* **121**, 1073–1076 (2013). 23223356 10.1182/blood-2012-07-445858 PMC3779401

[463] Carmo, M. et al. Perforin gene transfer into hematopoietic stem cells improves immune dysregulation in murine models of perforin deficiency. *Mol. Ther.* **23**, 737–745 (2015). 25523759 10.1038/mt.2014.242 PMC4395774

[464] Soheili, T. et al. Gene transfer into hematopoietic stem cells reduces HLH manifestations in a murine model of Munc13-4 deficiency. *Blood Adv.* **1**, 2781–2789 (2017). 29296930 10.1182/bloodadvances.2017012088 PMC5745141

[465] Benmebarek, M. R. et al. Killing mechanisms of chimeric antigen receptor (CAR) T cells. *Int. J. Mol. Sci.* **20**, 1283 (2019). 30875739 10.3390/ijms20061283 PMC6470706

[466] Feins, S., Kong, W., Williams, E. F., Milone, M. C. & Fraietta, J. A. An introduction to chimeric antigen receptor (CAR) T-cell immunotherapy for human cancer. *Am. J. Hematol.* **94**, S3–s9 (2019). 30680780 10.1002/ajh.25418

[467] Gödel, P., Shimabukuro-Vornhagen, A. & von Bergwelt-Baildon, M. Understanding cytokine release syndrome. *Intensive Care Med.* **44**, 371–373 (2018). 28956093 10.1007/s00134-017-4943-5

[468] Hay, K. A. et al. Kinetics and biomarkers of severe cytokine release syndrome after CD19 chimeric antigen receptor-modified T-cell therapy. *Blood* **130**, 2295–2306 (2017). 28924019 10.1182/blood-2017-06-793141 PMC5701525

[469] Guo, H., Qian, L. & Cui, J. Focused evaluation of the roles of macrophages in chimeric antigen receptor (CAR) T cell therapy associated cytokine release syndrome. *Cancer Biol. Med.* **19**, 333–342 (2021). 34570442 10.20892/j.issn.2095-3941.2021.0087 PMC8958886

[470] Giavridis, T. et al. CAR T cell-induced cytokine release syndrome is mediated by macrophages and abated by IL-1 blockade. *Nat. Med.* **24**, 731–738 (2018). 29808005 10.1038/s41591-018-0041-7 PMC6410714

[471] Maude, S. L., Barrett, D., Teachey, D. T. & Grupp, S. A. Managing cytokine release syndrome associated with novel T cell-engaging therapies. *Cancer J.* **20**, 119–122 (2014). 24667956 10.1097/PPO.0000000000000035 PMC4119809

[472] Davila, M. L. et al. Efficacy and toxicity management of 19-28z CAR T cell therapy in B cell acute lymphoblastic leukemia. *Sci. Transl. Med.* **6**, 224ra225 (2014). 10.1126/scitranslmed.3008226 PMC4684949 24553386

[473] Obstfeld, A. E. et al. Cytokine release syndrome associated with chimeric-antigen receptor T-cell therapy: clinicopathological insights. *Blood* **130**, 2569–2572 (2017). 29074500 10.1182/blood-2017-08-802413

[474] Singh, N. et al. Monocyte lineage-derived IL-6 does not affect chimeric antigen receptor T-cell function. *Cytotherapy* **19**, 867–880 (2017). 28506444 10.1016/j.jcyt.2017.04.001 PMC6676485

[475] Dinarello, C. A. Immunological and inflammatory functions of the interleukin-1 family. *Annu. Rev. Immunol.* **27**, 519–550 (2009). 19302047 10.1146/annurev.immunol.021908.132612

[476] Hamilton, J. A., Cook, A. D. & Tak, P. P. Anti-colony-stimulating factor therapies for inflammatory and autoimmune diseases. *Nat. Rev. Drug Discov.* **16**, 53–70 (2016). 28031576 10.1038/nrd.2016.231

[477] Sachdeva, M., Duchateau, P., Depil, S., Poirot, L. & Valton, J. Granulocyte-macrophage colony-stimulating factor inactivation in CAR T-cells prevents monocyte-dependent release of key cytokine release syndrome mediators. *J. Biol. Chem.* **294**, 5430–5437 (2019). 30804212 10.1074/jbc.AC119.007558 PMC6462525

[478] Sterner, R. M. et al. GM-CSF inhibition reduces cytokine release syndrome and neuroinflammation but enhances CAR-T cell function in xenografts. *Blood* **133**, 697–709 (2019). 30463995 10.1182/blood-2018-10-881722 PMC6376281

[479] Vogel, D. Y. et al. GM-CSF promotes migration of human monocytes across the blood brain barrier. *Eur. J. Immunol.* **45**, 1808–1819 (2015). 25756873 10.1002/eji.201444960

[480] Johnson, J. D. et al. Catecholamines mediate stress-induced increases in peripheral and central inflammatory cytokines. *Neuroscience* **135**, 1295–1307 (2005). 16165282 10.1016/j.neuroscience.2005.06.090

[481] Staedtke, V. et al. Disruption of a self-amplifying catecholamine loop reduces cytokine release syndrome. *Nature* **564**, 273–277 (2018). 30542164 10.1038/s41586-018-0774-y PMC6512810

[482] Brudno, J. N. & Kochenderfer, J. N. Current understanding and management of CAR T cell-associated toxicities. *Nat. Rev. Clin. Oncol.* **21**, 501–521 (2024). 38769449 10.1038/s41571-024-00903-0 PMC11529341

[483] Lee, D. W. et al. ASTCT consensus grading for cytokine release syndrome and neurologic toxicity associated with immune effector cells. *Biol. Blood Marrow Transpl.* **25**, 625–638 (2019). 10.1016/j.bbmt.2018.12.758 PMC12180426 30592986

[484] Zhou, L. et al. Derivation and validation of a novel score for early prediction of severe CRS after CAR-T therapy in haematological malignancy patients: a multi-centre study. *Br. J. Haematol.* **202**, 517–524 (2023). 37192741 10.1111/bjh.18873

[485] Wei, Z. et al. Prediction of severe CRS and determination of biomarkers in B cell-acute lymphoblastic leukemia treated with CAR-T cells. *Front. Immunol.* **14**, 1273507 (2023). 37854590 10.3389/fimmu.2023.1273507 PMC10579557

[486] Moreno-Castaño, A. B. et al. Characterization of the endotheliopathy, innate-immune activation and hemostatic imbalance underlying CAR-T cell toxicities: laboratory tools for an early and differential diagnosis. *J. Immunother. Cancer* **11**, e006365 (2023). 37045474 10.1136/jitc-2022-006365 PMC10106034

[487] Hong, F. et al. Predictive role of endothelial cell activation in cytokine release syndrome after chimeric antigen receptor T cell therapy for acute lymphoblastic leukaemia. *J. Cell Mol. Med.* **25**, 11063–11074 (2021). 34734474 10.1111/jcmm.17029 PMC8650023

[488] Korell, F. et al. EASIX and severe endothelial complications after CD19-directed CAR-T cell therapy—a cohort study. *Front. Immunol.* **13**, 877477 (2022). 35464403 10.3389/fimmu.2022.877477 PMC9033201

[489] Greenbaum, U. et al. CRP and ferritin in addition to the EASIX score predict CAR-T-related toxicity. *Blood Adv.* **5**, 2799–2806 (2021). 34264268 10.1182/bloodadvances.2021004575 PMC8341350

[490] Pennisi, M. et al. Modified EASIX predicts severe cytokine release syndrome and neurotoxicity after chimeric antigen receptor T cells. *Blood Adv.* **5**, 3397–3406 (2021). 34432870 10.1182/bloodadvances.2020003885 PMC8525234

[491] Xiao, X. et al. Mechanisms of cytokine release syndrome and neurotoxicity of CAR T-cell therapy and associated prevention and management strategies. *J. Exp. Clin. Cancer Res.* **40**, 367 (2021). 34794490 10.1186/s13046-021-02148-6 PMC8600921

[492] Norelli, M. et al. Monocyte-derived IL-1 and IL-6 are differentially required for cytokine-release syndrome and neurotoxicity due to CAR T cells. *Nat. Med.* **24**, 739–748 (2018). 29808007 10.1038/s41591-018-0036-4

[493] Frey, N. & Porter, D. Cytokine release syndrome with chimeric antigen receptor T cell therapy. *Biol. Blood Marrow Transpl.* **25**, e123–e127 (2019). 10.1016/j.bbmt.2018.12.756 30586620

[494] Wang, X. et al. Impact of tocilizumab on anti-CD19 chimeric antigen receptor T-cell therapy in B-cell acute lymphoblastic leukemia. *Cancer* **130**, 2660–2669 (2024). 38578977 10.1002/cncr.35316

[495] Leclercq, G. et al. Novel strategies for the mitigation of cytokine release syndrome induced by T cell engaging therapies with a focus on the use of kinase inhibitors. *Oncoimmunology* **11**, 2083479 (2022). 35694193 10.1080/2162402X.2022.2083479 PMC9176235

[496] Kadauke, S. et al. Risk-adapted preemptive tocilizumab to prevent severe cytokine release syndrome after CTL019 for pediatric B-Cell acute lymphoblastic leukemia: a prospective clinical trial. *J. Clin. Oncol.* **39**, 920–930 (2021). 33417474 10.1200/JCO.20.02477 PMC8462622

[497] Yakoub-Agha, I. et al. Management of adults and children undergoing chimeric antigen receptor T-cell therapy: best practice recommendations of the European Society for Blood and Marrow Transplantation (EBMT) and the Joint Accreditation Committee of ISCT and EBMT (JACIE). *Haematologica* **105**, 297–316 (2020). 31753925 10.3324/haematol.2019.229781 PMC7012497

[498] Strati, P. et al. Clinical efficacy of anakinra to mitigate CAR T-cell therapy-associated toxicity in large B-cell lymphoma. *Blood Adv.* **4**, 3123–3127 (2020). 32645136 10.1182/bloodadvances.2020002328 PMC7362369

[499] Diorio, C. et al. Anakinra utilization in refractory pediatric CAR T-cell associated toxicities. *Blood Adv.* **6**, 3398–3403 (2022). 35395068 10.1182/bloodadvances.2022006983 PMC9198909

[500] Harris, A. C. et al. International, multicenter standardization of acute graft-versus-host disease clinical data collection: a report from the Mount Sinai Acute GVHD International Consortium. *Biol. Blood Marrow Transpl.* **22**, 4–10 (2016). 10.1016/j.bbmt.2015.09.001 PMC4706482 26386318

[501] Ghimire, S. et al. Pathophysiology of GvHD and other HSCT-related major complications. *Front. Immunol.* **8**, 79 (2017). 28373870 10.3389/fimmu.2017.00079 PMC5357769

[502] Blazar, B. R., Murphy, W. J. & Abedi, M. Advances in graft-versus-host disease biology and therapy. *Nat. Rev. Immunol.* **12**, 443–458 (2012). 22576252 10.1038/nri3212 PMC3552454

[503] Malard, F., Huang, X. J. & Sim, J. P. Y. Treatment and unmet needs in steroid-refractory acute graft-versus-host disease. *Leukemia* **34**, 1229–1240 (2020). 32242050 10.1038/s41375-020-0804-2 PMC7192843

[504] Zeiser, R. & Blazar, B. R. Acute graft-versus-host disease—biologic process, prevention, and therapy. *N. Engl. J. Med.* **377**, 2167–2179 (2017). 29171820 10.1056/NEJMra1609337 PMC6034180

[505] Koyama, M. & Hill, G. R. The primacy of gastrointestinal tract antigen-presenting cells in lethal graft-versus-host disease. *Blood* **134**, 2139–2148 (2019). 31697827 10.1182/blood.2019000823 PMC6908833

[506] Tugues, S. et al. Graft-versus-host disease, but not graft-versus-leukemia immunity, is mediated by GM-CSF-licensed myeloid cells. *Sci. Transl. Med.* **10**, eaat8410 (2018). 30487251 10.1126/scitranslmed.aat8410

[507] Wen, Q. et al. Glucocorticoid and glycolysis inhibitors cooperatively abrogate acute graft-versus-host disease. *Sci. China Life Sci.* **66**, 528–544 (2023). 36166182 10.1007/s11427-022-2170-2

[508] Wu, X. et al. Prediction of acute GVHD and relapse by metabolic biomarkers after allogeneic hematopoietic stem cell transplantation. *JCI Insight* **3**, e99672 (2018). 29720575 10.1172/jci.insight.99672 PMC6012513

[509] Han, L. et al. A gut microbiota score predicting acute graft-versus-host disease following myeloablative allogeneic hematopoietic stem cell transplantation. *Am. J. Transpl.* **20**, 1014–1027 (2020). 10.1111/ajt.15654 PMC7154648 31605563

[510] Sorror, M. L. et al. Pretransplant comorbidities predict severity of acute graft-versus-host disease and subsequent mortality. *Blood* **124**, 287–295 (2014). 24797298 10.1182/blood-2014-01-550566 PMC4093684

[511] Patel, D. A., Crain, M., Pusic, I. & Schroeder, M. A. Acute graft-versus-host disease: an update on new treatment options. *Drugs* **83**, 893–907 (2023). 37247105 10.1007/s40265-023-01889-2

[512] McDonald, G. B. et al. Plasma biomarkers of acute GVHD and nonrelapse mortality: predictive value of measurements before GVHD onset and treatment. *Blood* **126**, 113–120 (2015). 25987657 10.1182/blood-2015-03-636753 PMC4492194

[513] Hartwell, M. J. et al. An early-biomarker algorithm predicts lethal graft-versus-host disease and survival. *JCI Insight* **2**, e89798 (2017). 28194439 10.1172/jci.insight.89798 PMC5291735

[514] Aziz, M. D. et al. Disease risk and GVHD biomarkers can stratify patients for risk of relapse and nonrelapse mortality post hematopoietic cell transplant. *Leukemia* **34**, 1898–1906 (2020). 32020045 10.1038/s41375-020-0726-z PMC7332389

[515] Luft, T. et al. Steroid-refractory GVHD: T-cell attack within a vulnerable endothelial system. *Blood* **118**, 1685–1692 (2011). 21636856 10.1182/blood-2011-02-334821

[516] Ueda, N. et al. Predictive value of circulating angiopoietin-2 for endothelial damage-related complications in allogeneic hematopoietic stem cell transplantation. *Biol. Blood Marrow Transpl.* **20**, 1335–1340 (2014). 10.1016/j.bbmt.2014.04.030 24796281

[517] Jo, T. et al. Author Correction: a convolutional neural network-based model that predicts acute graft-versus-host disease after allogeneic hematopoietic stem cell transplantation. *Commun. Med.* **3**, 89 (2023). 37349499 10.1038/s43856-023-00323-8 PMC10287704

[518] Martínez-Laperche, C. et al. A novel predictive approach for GVHD after allogeneic SCT based on clinical variables and cytokine gene polymorphisms. *Blood Adv.* **2**, 1719–1737 (2018). 30030270 10.1182/bloodadvances.2017011502 PMC6058238

[519] Hess, N. J. et al. Inflammatory CD4/CD8 double-positive human T cells arise from reactive CD8 T cells and are sufficient to mediate GVHD pathology. *Sci. Adv.* **9**, eadf0567 (2023). 36961891 10.1126/sciadv.adf0567 PMC10038349

[520] Penack, O. et al. Prophylaxis and management of graft-versus-host disease after stem-cell transplantation for haematological malignancies: updated consensus recommendations of the European Society for Blood and Marrow Transplantation. *Lancet Haematol.* **11**, e147–e159 (2024). 38184001 10.1016/S2352-3026(23)00342-3

[521] Xu, L. et al. The consensus on indications, conditioning regimen, and donor selection of allogeneic hematopoietic cell transplantation for hematological diseases in China-recommendations from the Chinese Society of Hematology. *J. Hematol. Oncol.* **11**, 33 (2018). 29495966 10.1186/s13045-018-0564-x PMC5833104

[522] Huang, X. J. et al. The superiority of haploidentical related stem cell transplantation over chemotherapy alone as postremission treatment for patients with intermediate- or high-risk acute myeloid leukemia in first complete remission. *Blood* **119**, 5584–5590 (2012). 22535659 10.1182/blood-2011-11-389809

[523] Huang, X. J. et al. A novel approach to human leukocyte antigen-mismatched transplantation in patients with malignant hematological disease. *Chin. Med. J.* **117**, 1778–1785 (2004). 15603704

[524] Luo, Y. et al. T-cell-replete haploidentical HSCT with low-dose anti-T-lymphocyte globulin compared with matched sibling HSCT and unrelated HSCT. *Blood* **124**, 2735–2743 (2014). 25214441 10.1182/blood-2014-04-571570 PMC4208287

[525] Danylesko, I. et al. Anti-α4β7 integrin monoclonal antibody (vedolizumab) for the treatment of steroid-resistant severe intestinal acute graft-versus-host disease. *Bone Marrow Transpl.* **54**, 987–993 (2019). 10.1038/s41409-018-0364-5 30356163

[526] Khuat, L. T. et al. Increased efficacy of dual proinflammatory cytokine blockade on acute GVHD while maintaining GVT effects. *Blood* **138**, 2583–2588 (2021). 34424962 10.1182/blood.2021011216 PMC8678998

[527] Wang, R. et al. USP11 plays a critical role in the onset and progression of acute graft-versus-host disease: Novel target for precision therapeutics. *Pharm. Res.* **189**, 106707 (2023). 10.1016/j.phrs.2023.106707 36822452

[528] Wehrli, M. et al. Single-center experience using anakinra for steroid-refractory immune effector cell-associated neurotoxicity syndrome (ICANS). *J. Immunother. Cancer* **10**, e003847 (2022). 34996813 10.1136/jitc-2021-003847 PMC8744112

[529] Nordström, D. et al. Beneficial effect of interleukin 1 inhibition with anakinra in adult-onset Still’s disease. An open, randomized, multicenter study. *J. Rheumatol.* **39**, 2008–2011 (2012). 22859346 10.3899/jrheum.111549

[530] Quartier, P. et al. A multicentre, randomised, double-blind, placebo-controlled trial with the interleukin-1 receptor antagonist anakinra in patients with systemic-onset juvenile idiopathic arthritis (ANAJIS trial). *Ann. Rheum. Dis.* **70**, 747–754 (2011). 21173013 10.1136/ard.2010.134254 PMC3070271

[531] Kraft, L., Erdenesukh, T., Sauter, M., Tschöpe, C. & Klingel, K. Blocking the IL-1β signalling pathway prevents chronic viral myocarditis and cardiac remodeling. *Basic Res. Cardiol.* **114**, 11 (2019). 30673858 10.1007/s00395-019-0719-0

[532] Kotch, C., Barrett, D. & Teachey, D. T. Tocilizumab for the treatment of chimeric antigen receptor T cell-induced cytokine release syndrome. *Expert Rev. Clin. Immunol.* **15**, 813–822 (2019). 31219357 10.1080/1744666X.2019.1629904 PMC7936577

[533] Stone, J. H. et al. Trial of tocilizumab in giant-cell arteritis. *N. Engl. J. Med.* **377**, 317–328 (2017). 28745999 10.1056/NEJMoa1613849

[534] Casper, C. et al. Analysis of inflammatory and anemia-related biomarkers in a randomized, double-blind, placebo-controlled study of siltuximab (Anti-IL6 Monoclonal Antibody) in patients with multicentric castleman disease. *Clin. Cancer Res.* **21**, 4294–4304 (2015). 26124203 10.1158/1078-0432.CCR-15-0134

[535] Gauthier, J. & Turtle, C. J. Insights into cytokine release syndrome and neurotoxicity after CD19-specific CAR-T cell therapy. *Curr. Res. Transl. Med.* **66**, 50–52 (2018). 29625831 10.1016/j.retram.2018.03.003 PMC5967886

[536] Albeituni, S. et al. Mechanisms of action of ruxolitinib in murine models of hemophagocytic lymphohistiocytosis. *Blood* **134**, 147–159 (2019). 31015190 10.1182/blood.2019000761 PMC6624972

[537] Kalil, A. C. et al. Baricitinib plus remdesivir for hospitalized adults with Covid-19. *N. Engl. J. Med.* **384**, 795–807 (2021). 33306283 10.1056/NEJMoa2031994 PMC7745180

[538] Wang, S. et al. Cellular nanosponges for biological neutralization. *Adv. Mater.* **34**, e2107719 (2022). 34783078 10.1002/adma.202107719

[539] Wang, H. et al. Cytokine nanosponges suppressing overactive macrophages and dampening systematic cytokine storm for the treatment of hemophagocytic lymphohistiocytosis. *Bioact. Mater.* **21**, 531–546 (2023). 36185750 10.1016/j.bioactmat.2022.09.012 PMC9508173

[540] Zhang, Q. et al. Neutrophil membrane-coated nanoparticles inhibit synovial inflammation and alleviate joint damage in inflammatory arthritis. *Nat. Nanotechnol.* **13**, 1182–1190 (2018). 30177807 10.1038/s41565-018-0254-4

[541] Dominici, M. et al. Minimal criteria for defining multipotent mesenchymal stromal cells. The International Society for Cellular Therapy position statement. *Cytotherapy* **8**, 315–317 (2006). 16923606 10.1080/14653240600855905

[542] Uccelli, A., Moretta, L. & Pistoia, V. Mesenchymal stem cells in health and disease. *Nat. Rev. Immunol.* **8**, 726–736 (2008). 19172693 10.1038/nri2395

[543] Chen, P. M., Yen, M. L., Liu, K. J., Sytwu, H. K. & Yen, B. L. Immunomodulatory properties of human adult and fetal multipotent mesenchymal stem cells. *J. Biomed. Sci.* **18**, 49 (2011). 21762539 10.1186/1423-0127-18-49 PMC3156728

[544] Wang, L. T., Liu, K. J., Sytwu, H. K., Yen, M. L. & Yen, B. L. Advances in mesenchymal stem cell therapy for immune and inflammatory diseases: Use of cell-free products and human pluripotent stem cell-derived mesenchymal stem cells. *Stem Cells Transl. Med.* **10**, 1288–1303 (2021). 34008922 10.1002/sctm.21-0021 PMC8380447

[545] Sioud, M. New insights into mesenchymal stromal cell-mediated T-cell suppression through galectins. *Scand. J. Immunol.* **73**, 79–84 (2011). 21198747 10.1111/j.1365-3083.2010.02491.x

[546] Wang, L. T. et al. Human mesenchymal stem cells (MSCs) for treatment towards immune- and inflammation-mediated diseases: review of current clinical trials. *J. Biomed. Sci.* **23**, 76 (2016). 27809910 10.1186/s12929-016-0289-5 PMC5095977

[547] Le Blanc, K. et al. Treatment of severe acute graft-versus-host disease with third party haploidentical mesenchymal stem cells. *Lancet* **363**, 1439–1441 (2004). 15121408 10.1016/S0140-6736(04)16104-7

[548] Lu, W. et al. Efficacy and safety of mesenchymal stem cells therapy in COVID-19 patients: a systematic review and meta-analysis of randomized controlled trials. *J. Transl. Med.* **22**, 550 (2024). 38851730 10.1186/s12967-024-05358-6 PMC11162060

[549] Shi, L. et al. Effect of human umbilical cord-derived mesenchymal stem cells on lung damage in severe COVID-19 patients: a randomized, double-blind, placebo-controlled phase 2 trial. *Signal Transduct. Target. Ther.* **6**, 58 (2021). 33568628 10.1038/s41392-021-00488-5 PMC7873662

[550] Monsel, A. et al. Treatment of COVID-19-associated ARDS with mesenchymal stromal cells: a multicenter randomized double-blind trial. *Crit. Care* **26**, 48 (2022). 35189925 10.1186/s13054-022-03930-4 PMC8860258

[551] Tsuchida, K., Yoshimura, R., Nakatani, T. & Takemoto, Y. Blood purification for critical illness: cytokines adsorption therapy. *Ther. Apher. Dial.* **10**, 25–31 (2006). 16556133 10.1111/j.1744-9987.2006.00342.x

[552] Sekiya, Y. et al. Evaluation of a simultaneous adsorption device for cytokines and platelet-neutrophil complexes in vitro and in a rabbit acute lung injury model. *Intensive Care Med. Exp.* **9**, 49 (2021). 34568985 10.1186/s40635-021-00414-7 PMC8473513

[553] Wu, Y. et al. Hemophagocytic lymphohistiocytosis: current treatment advances, emerging targeted therapy and underlying mechanisms. *J. Hematol. Oncol.* **17**, 106 (2024). 39511607 10.1186/s13045-024-01621-x PMC11542428

[554] Bode, S. F. et al. Recent advances in the diagnosis and treatment of hemophagocytic lymphohistiocytosis. *Arthritis Res. Ther.* **14**, 213 (2012). 22682420 10.1186/ar3843 PMC3446494

[555] Meyer, N. J., Gattinoni, L. & Calfee, C. S. Acute respiratory distress syndrome. *Lancet* **398**, 622–637 (2021). 34217425 10.1016/S0140-6736(21)00439-6 PMC8248927

[556] Neelapu, S. S. et al. Chimeric antigen receptor T-cell therapy—assessment and management of toxicities. *Nat. Rev. Clin. Oncol.* **15**, 47–62 (2018). 28925994 10.1038/nrclinonc.2017.148 PMC6733403

[557] Goldman, A. et al. Adverse cardiovascular and pulmonary events associated with chimeric antigen receptor T-cell therapy. *J. Am. Coll. Cardiol.* **78**, 1800–1813 (2021). 34711339 10.1016/j.jacc.2021.08.044 PMC8562317

[558] Baker, R., Liew, J. W., Simonson, P. D., Soma, L. A. & Starkebaum, G. Macrophage activation syndrome in a patient with axial spondyloarthritis on adalimumab. *Clin. Rheumatol.* **38**, 603–608 (2019). 30535729 10.1007/s10067-018-4387-5 PMC7087649

[559] Bonelli, M. et al. Selectivity, efficacy and safety of JAKinibs: new evidence for a still evolving story. *Ann. Rheum. Dis.* **83**, 139–160 (2024). 37923366 10.1136/ard-2023-223850 PMC10850682

[560] Adas, M. A. et al. The infection risks of JAK inhibition. *Expert Rev. Clin. Immunol.* **18**, 253–261 (2022). 34860621 10.1080/1744666X.2022.2014323 PMC8935945

[561] Chaturvedi, V., Lakes, N., Tran, M., Castillo, N. & Jordan, M. B. JAK inhibition for murine HLH requires complete blockade of IFN-γ signaling and is limited by toxicity of JAK2 inhibition. *Blood* **138**, 1034–1039 (2021). 34232994 10.1182/blood.2020007930

[562] Griffith, B. P. et al. Genetically modified porcine-to-human cardiac xenotransplantation. *N. Engl. J. Med.* **387**, 35–44 (2022). 35731912 10.1056/NEJMoa2201422 PMC10361070

[563] Dinarello, C. A. Interleukin-1 in the pathogenesis and treatment of inflammatory diseases. *Blood* **117**, 3720–3732 (2011). 21304099 10.1182/blood-2010-07-273417 PMC3083294

[564] Abbas, A. K., Trotta, E., Simeonov, D. R., Marson, A. & Bluestone, J. A. Revisiting IL-2: biology and therapeutic prospects. *Sci. Immunol.* **3**, eaat1482 (2018). 29980618 10.1126/sciimmunol.aat1482

[565] Calabrese, L. H. & Rose-John, S. IL-6 biology: implications for clinical targeting in rheumatic disease. *Nat. Rev. Rheumatol.* **10**, 720–727 (2014). 25136784 10.1038/nrrheum.2014.127

[566] Rauber, S. et al. Resolution of inflammation by interleukin-9-producing type 2 innate lymphoid cells. *Nat. Med.* **23**, 938–944 (2017). 28714991 10.1038/nm.4373 PMC5575995

[567] York, A. G. et al. IL-10 constrains sphingolipid metabolism to limit inflammation. *Nature* **627**, 628–635 (2024). 38383790 10.1038/s41586-024-07098-5 PMC10954550

[568] Teng, M. W. et al. IL-12 and IL-23 cytokines: from discovery to targeted therapies for immune-mediated inflammatory diseases. *Nat. Med.* **21**, 719–729 (2015). 26121196 10.1038/nm.3895

[569] Melo-Cardenas, J. et al. IL-13/IL-4 signaling contributes to fibrotic progression of the myeloproliferative neoplasms. *Blood* **140**, 2805–2817 (2022). 36283106 10.1182/blood.2022017326 PMC9813832

[570] Huangfu, L., Li, R., Huang, Y. & Wang, S. The IL-17 family in diseases: from bench to bedside. *Signal Transduct. Target. Ther.* **8**, 402 (2023). 37816755 10.1038/s41392-023-01620-3 PMC10564932

[571] Landy, E., Carol, H., Ring, A. & Canna, S. Biological and clinical roles of IL-18 in inflammatory diseases. *Nat. Rev. Rheumatol.* **20**, 33–47 (2024). 38081945 10.1038/s41584-023-01053-w PMC12337794

[572] Zander, R. et al. Tfh-cell-derived interleukin 21 sustains effector CD8(+) T cell responses during chronic viral infection. *Immunity* **55**, 475–493.e475 (2022). 35216666 10.1016/j.immuni.2022.01.018 PMC8916994

[573] Keir, M., Yi, Y., Lu, T. & Ghilardi, N. The role of IL-22 in intestinal health and disease. *J. Exp. Med.* **217**, e20192195 (2020). 32997932 10.1084/jem.20192195 PMC7062536

[574] Fassett, M. S. et al. IL-31-dependent neurogenic inflammation restrains cutaneous type 2 immune response in allergic dermatitis. *Sci. Immunol.* **8**, eabi6887 (2023). 37831760 10.1126/sciimmunol.abi6887 PMC10890830

[575] Liew, F. Y., Girard, J. P. & Turnquist, H. R. Interleukin-33 in health and disease. *Nat. Rev. Immunol.* **16**, 676–689 (2016). 27640624 10.1038/nri.2016.95

[576] Banchereau, J., Pascual, V. & O’Garra, A. From IL-2 to IL-37: the expanding spectrum of anti-inflammatory cytokines. *Nat. Immunol.* **13**, 925–931 (2012). 22990890 10.1038/ni.2406 PMC3609707

[577] Ivashkiv, L. B. & Donlin, L. T. Regulation of type I interferon responses. *Nat. Rev. Immunol.* **14**, 36–49 (2014). 24362405 10.1038/nri3581 PMC4084561

[578] Deczkowska, A., Baruch, K. & Schwartz, M. Type I/II interferon balance in the regulation of brain physiology and pathology. *Trends Immunol.* **37**, 181–192 (2016). 26877243 10.1016/j.it.2016.01.006

[579] Deng, Z. et al. TGF-β signaling in health, disease, and therapeutics. *Signal Transduct. Target. Ther.* **9**, 61 (2024). 38514615 10.1038/s41392-024-01764-w PMC10958066

[580] Kalliolias, G. D. & Ivashkiv, L. B. TNF biology, pathogenic mechanisms and emerging therapeutic strategies. *Nat. Rev. Rheumatol.* **12**, 49–62 (2016). 26656660 10.1038/nrrheum.2015.169 PMC4809675

[581] Yoshimura, T. The chemokine MCP-1 (CCL2) in the host interaction with cancer: a foe or ally? *Cell. Mol. Immunol.* **15**, 335–345 (2018). 29375123 10.1038/cmi.2017.135 PMC6052833

[582] Jia, S. N., Han, Y. B., Yang, R. & Yang, Z. C. Chemokines in colon cancer progression. *Semin. Cancer Biol.* **86**, 400–407 (2022). 35183412 10.1016/j.semcancer.2022.02.007

[583] Zhao, N. et al. Intratumoral γδ T-cell infiltrates, chemokine (C-C Motif) ligand 4/chemokine (C-C Motif) ligand 5 protein expression and survival in patients with hepatocellular carcinoma. *Hepatology* **73**, 1045–1060 (2021). 32502310 10.1002/hep.31412 PMC9175512

[584] Ha, H., Debnath, B. & Neamati, N. Role of the CXCL8-CXCR1/2 axis in cancer and inflammatory diseases. *Theranostics* **7**, 1543–1588 (2017). 28529637 10.7150/thno.15625 PMC5436513

[585] Tokunaga, R. et al. CXCL9, CXCL10, CXCL11/CXCR3 axis for immune activation—a target for novel cancer therapy. *Cancer Treat. Rev.* **63**, 40–47 (2018). 29207310 10.1016/j.ctrv.2017.11.007 PMC5801162

[586] Wang, B., Wang, M., Ao, D. & Wei, X. CXCL13-CXCR5 axis: regulation in inflammatory diseases and cancer. *Biochim. Biophys. Acta Rev. Cancer* **188799**, 2022 (**1877**). 10.1016/j.bbcan.2022.188799 36103908

[587] Goyal, R. et al. Comparative highlights on MERS-CoV, SARS-CoV-1, SARS-CoV-2, and NEO-CoV. *EXCLI J.* **21**, 1245–1272 (2022). 36483910 10.17179/excli2022-5355 PMC9727256

[588] Xu, R. et al. Role of cytokine storm in coronavirus infections: culprit or accomplice? *Front. Biosci.* **27**, 102 (2022). 10.31083/j.fbl2703102 35345334

[589] Petrone, L. et al. A whole blood test to measure SARS-CoV-2-specific response in COVID-19 patients. *Clin. Microbiol. Infect.* **27**, 286.e287–e213 (2021). 10.1016/j.cmi.2020.09.051 PMC7547312 33045370

[590] Pandey, P. & Karupiah, G. Targeting tumour necrosis factor to ameliorate viral pneumonia. *FEBS J.* **289**, 883–900 (2022). 33624419 10.1111/febs.15782

[591] Castro, C. N. et al. NCKAP1L defects lead to a novel syndrome combining immunodeficiency, lymphoproliferation, and hyperinflammation. *J. Exp. Med.* **217**, e20192275 (2020). 32766723 10.1084/jem.20192275 PMC7526481

[592] Tavernier, S. J. et al. A human immune dysregulation syndrome characterized by severe hyperinflammation with a homozygous nonsense Roquin-1 mutation. *Nat. Commun.* **10**, 4779 (2019). 31636267 10.1038/s41467-019-12704-6 PMC6803705

[593] Kalinichenko, A. et al. RhoG deficiency abrogates cytotoxicity of human lymphocytes and causes hemophagocytic lymphohistiocytosis. *Blood* **137**, 2033–2045 (2021). 33513601 10.1182/blood.2020008738 PMC8057258

[594] Drago, E. et al. Case report: susceptibility to viral infections and secondary hemophagocytic lymphohistiocytosis responsive to intravenous immunoglobulin as primary manifestations of adenosine deaminase 2 deficiency. *Front. Immunol.* **13**, 937108 (2022). 36159847 10.3389/fimmu.2022.937108 PMC9503826

[595] Arduini, A. et al. An unusual presentation of purine nucleoside phosphorylase deficiency mimicking systemic juvenile idiopathic arthritis complicated by macrophage activation syndrome. *Pediatr. Rheumatol. Online J.* **17**, 25 (2019). 31118063 10.1186/s12969-019-0328-3 PMC6532153

[596] Wu, S., Gonzalez-Gomez, I., Coates, T. & Yano, S. Cobalamin C disease presenting with hemophagocytic lymphohistiocytosis. *Pediatr. Hematol. Oncol.* **22**, 717–721 (2005). 16251179 10.1080/08880010500278871

[597] Vignesh, P. et al. Features of hemophagocytic lymphohistiocytosis in infants with severe combined immunodeficiency: our experience from Chandigarh, North India. *Front. Immunol.* **13**, 867753 (2022). 35812426 10.3389/fimmu.2022.867753 PMC9260510

[598] Han, S. P., Lin, Y. F., Weng, H. Y., Tsai, S. F. & Fu, L. S. A novel BTK gene mutation in a child with atypical X-linked agammaglobulinemia and recurrent hemophagocytosis: a case report. *Front. Immunol.* **10**, 1953 (2019). 31481959 10.3389/fimmu.2019.01953 PMC6711359

[599] Nittner-Marszalska, M. et al. Hemophagocytic lymphohistiocytosis (HLH) triggered by wasp venom immunotherapy is rare but noteworthy. *J. Investig. Allergol. Clin. Immunol.* **29**, 49–51 (2019). 30785100 10.18176/jiaci.0322

[600] Amayiri, N., Al-Zaben, A., Ghatasheh, L., Frangoul, H. & Hussein, A. A. Hematopoietic stem cell transplantation for children with primary immunodeficiency diseases: single center experience in Jordan. *Pediatr. Transpl.* **17**, 394–402 (2013). 10.1111/petr.12081 23692601

[601] Tanaka, T. et al. National survey of Japanese patients with mevalonate kinase deficiency reveals distinctive genetic and clinical characteristics. *Mod. Rheumatol.* **29**, 181–187 (2019). 29451047 10.1080/14397595.2018.1442639

[602] Gokce, M. et al. Secondary hemophagocytosis in 3 patients with organic acidemia involving propionate metabolism. *Pediatr. Hematol. Oncol.* **29**, 92–98 (2012). 21970506 10.3109/08880018.2011.601402

[603] Chau, A. S. et al. Heme oxygenase-1 deficiency presenting with interstitial lung disease and hemophagocytic flares. *Pediatr. Rheumatol. Online J.* **18**, 80 (2020). 33066778 10.1186/s12969-020-00474-1 PMC7565350

[604] Sesques, P. et al. Novel prognostic scoring systems for severe CRS and ICANS after anti-CD19 CAR T cells in large B-cell lymphoma. *J. Hematol. Oncol.* **17**, 61 (2024). 39107847 10.1186/s13045-024-01579-w PMC11305039

[605] Zhang, M. et al. Assessment and predictive ability of the absolute neutrophil count in peripheral blood for in vivo CAR T cells expansion and CRS. *J. Immunother. Cancer* **11**, e007790 (2023). 38016717 10.1136/jitc-2023-007790 PMC10685953

[606] Liu, Y. et al. A combination of pre-infusion serum ferritin, CRP and IL-6 predicts outcome in relapsed/refractory multiple myeloma patients treated with CAR-T cells. *Front. Immunol.* **14**, 1169071 (2023). 37153543 10.3389/fimmu.2023.1169071 PMC10154462

[607] Berg, A. F. et al. Exclusive inhibition of IL-6 trans-signaling by soluble gp130(FlyR)Fc. *Cytokine. X* **3**, 100058 (2021). 34927050 10.1016/j.cytox.2021.100058 PMC8649222

[608] Liu, X. et al. Dynamic forecasting of severe acute graft-versus-host disease after transplantation. *Nat. Comput. Sci.* **2**, 153–159 (2022). 38177449 10.1038/s43588-022-00213-4 PMC10766514

[609] MacMillan, M. L. et al. Validation of Minnesota acute graft-versus-host disease Risk Score. *Haematologica* **105**, 519–524 (2020). 31320554 10.3324/haematol.2019.220970 PMC7012472

[610] Golob, J. L. et al. Stool microbiota at neutrophil recovery is predictive for severe acute graft vs host disease after hematopoietic cell transplantation. *Clin. Infect. Dis.* **65**, 1984–1991 (2017). 29020185 10.1093/cid/cix699 PMC5850019

[611] Levine, J. E. et al. A prognostic score for acute graft-versus-host disease based on biomarkers: a multicentre study. *Lancet Haematol.* **2**, e21–e29 (2015). 26687425 10.1016/S2352-3026(14)00035-0 PMC4340092

